# Supplementary material for: Spatiotemporal profiling of cytosolic signaling complexes in living cells by selective proximity proteomics
Source: Nat Commun. 2021 Jan 4;12:71. doi: 10.1038/s41467-020-20367-x (PMC7782698; doi:10.1038/s41467-020-20367-x)
Supplement: Supplementary file 1 — Supplementary Information [file 41467_2020_20367_MOESM1_ESM.pdf]

Supplementary information

Spatiotemporal Profiling of Cytosolic Signaling Complexes in Living Cells by Highly Selective Proximity Proteomics

Mi Ke[1,6], Xiao Yuan[1,6], An He[1], Peiyuan Yu[1], Wendong Chen[1], Yu Shi[2], Peng Zou[3], Tony Hunter[2], Ruijun Tian[1,4,5*]

[1] Department of Chemistry, School of Science, Southern University of Science and Technology, Shenzhen, China

[2] Molecular and Cell Biology Laboratory, Salk Institute for Biological Studies, La Jolla CA, USA

[3] College of Chemistry and Molecular Engineering, Peking University, Beijing, China

[4] Guangdong Provincial Key of Cell Microenvironment and Disease Research, Southern University of Science and Technology, Shenzhen, China

[5] Shenzhen Grubbs Institute, Southern University of Science and Technology, Shenzhen 518055, China

[6] The authors contributed equally.

[*] To whom correspondence should be addressed. Email: tianrj@sustech.edu.cn

Supplementary information includes:

1. Supplementary Methods (page 2-26)

2. Supplementary Figures 1-13 (page 27-48)

3. Supplementary References (page 48-50)

Supplementary Methods

(1) MS sample preparation

The streptavidin beads or FLAG M2 gel pull-down and wash conditions were described in **Methods.** After beads washing, the samples were reduced with 10 mM dithiothreitol (in 50 mM ABC) at 55 ^{o}C for 30 min and then alkylated by adding 30 mM of iodoacetamide (in 50 mM ABC) in dark at room temperature for 15 min. After that, 1.5 µg of trypsin in 50 mM ABC was added for overnight digestion at 37 ^{o}C. After trypsin digestion, the samples were desalted and dried in Speed Vac.

(2) Biotinylated peptide enrichment

Proximity labeling has been described in **Methods**. Peptide preparation and biotinylated peptide enrichment were performed according to a reported work with minor modification[1]. In detail, protein extracts were precipitated by methanol-chloroform protein precipitation. The obtained protein precipitate was dissolved in 8M urea and 50 mM Tris-HCl, pH 8.5, reduced with 10 mM dithiothreitol (SIGMA, 43816) at 55 ^{o}C for 25 min, alkylated with 30 mM iodoacetamide (SIGMA, I1149) in dark at room temperature for 30 min. Additional 20 mM of dithiothreitol was added to stop the reaction. The sample was then diluted by 7× volumes of 50 mM Tris-HCl, pH 8.5. Trypsin (SIGMA, T1426) was added according to the protein amount (1:20, w/w), and digestion was processed at 37 ^{o}C for 14h. The obtained peptides were acidified to pH 2-3 by adding 10% (v/v) TFA, and desalted with C18 SPE column (WAT054925). The desalted peptides were dried in SpeedVac and redissolved in affinity purification (AP) buffer (50 mM Tris-HCl, 10 mM Na_2HPO_4, 50 mM NaCl, pH 7.8) and further adjusted to pH 7~8 if required by 500 mM Tris-HCl, pH 8.5. For biotinylated peptide enrichment, 10 mg of tryptic peptides were incubated with 100 µL streptavidin agarose beads at 4 ^{o}C for overnight. When 2 mg of tryptic peptide was used for enrichment, the beads volume was 50 µL. After that, the beads were washed by the following steps: once with 1 M KCl in 50 mM Tris-HCl, pH 8.0; once with 2 M urea in 50 mM Tris-HCl, pH 8.0; twice with 5% (v/v) ACN in Dulbecco's Phosphate-Buffered Saline (DPBS); and four times with water. The enriched biotinylated peptides were eluted as the following steps: the beads were heated at 96 ^{o}C for 5 min after adding 200 µL of 80% (v/v) ACN, 0.2%

(v/v) TFA, and 0.1% (v/v) FA. This elution step was repeated for four additional times. After centrifuged at 16,612 ×g for 1 min, the eluted biotinylated peptides were combined and then dried for storage.

(3) MS analysis

Peptides were redissolved and analyzed with an Orbitrap Fusion mass spectrometer or Q Exactive HF-X (for comparison between BN2 and BP5) equipped with Easy-nanoLC (Thermo Fisher Scientific). The home-made capillary tip column (100 μm i.d. × 20 cm) was packed with ~0.5 cm of C4 (3 μm / 120 Å, Dr. Maisch GmbH) and ~20 cm of C18 resin (1.9 μm / 120 Å, Dr. Maisch GmbH). The mobile phase used for peptide separation were 0.1% (v/v) FA (solvent A) and 0.1% (v/v) FA in ACN (solvent B). The flow rate for the separation was set as 250 nL/min. LC gradient for solvent B was programmed as follows: 0 min, 3%; 2 min, 7%; 77 min, 22%; 92 min, 35%; 94 min, 90%; 100 min, 90%; 102 min, 3%; 110 min, 3%. For MS analysis for bait protein ILK, RSU1, PINCH, FUS, SHC1 and GRB2 (BP5 v.s. BN2, BP5 v.s. BP8), the following LC gradient was programmed: 0 min, 3%; 2 min, 7%; 52 min, 22%; 62 min, 35%; 64 min, 90%; 70 min, 90%; 72 min, 90%; 80 min, 3%. The LC gradient for biotinylated peptide enrichment is: 0 min, 3%; 2 min, 7%; 52 min, 28%; 62 min, 45%; 64 min, 90%; 80 min, 90%. The full MS scan range were set as 350−1550 and the mass resolution is 120000. Tandem MS was performed in a quadrupole mass analyzer with 1.6 Da isolation window and peptides were fragmented with collision energy of 30 (Orbitrap Fusion) or 27 (Q Exactive HF-X).

(4) Data analysis

Raw MS data were searched in MaxQuant software with label-free quantification function. The key settings were: digestion enzyme: trypsin/p and tolerated missed cleavage: 2. The human database from Uniprot was used (downloaded at 2017-08-19 with 70941 entries). Carbamidomethyl (C, +57 Da) was set as fix modification and Oxidation (M, +16 Da) modification was included as variable modification. APEX2-FLAG sequence was assembled with the bait protein sequences. “Match between runs” was set for label-free quantification. The false discovery rate (FDR) was evaluated by searching a reverse database and was set to 0.01 for proteins and peptides.

“Proteingroups.txt” table was used for generating volcano plot in Perseus software. The proteins were firstly filtered with “only identified by site”, “potential contaminant” and “reverse”. Proteins identified with at least 2 unique peptides were taken into consideration for further data analysis. Samples were then grouped into experimental and control groups, and only proteins with 3 valid values in at least one group were kept. The invalid values were randomly replaced against the total matrix with width 0.3 and down shift 1.8. Student’s T-test was operated between experimental and control groups, with FDR<0.05 and S0=0.5.

For biotinylated peptide related data analysis, the raw data files were searched in ProteinDiscover software (Thermo Fisher Scientific, PD1.4.1.14). The precursor and fragment mass tolerance were set as 10 ppm and 0.02 Da, respectively. The maximal missed cleavage was 2. Carbamidomethyl (C, +57.021 Da) was set as fix modification, Oxidation (M, +15.995 Da), Deamidation (N/Q, +0.984 Da) and BP5 (Y, +334.114 Da) modifications were included as variable modification. APEX2-FLAG peptide sequence was included into the N-terminal of GRB2 sequence. The FDR cutoff for the peptide spectrum matches (PSMs) and identified peptides were 0.05. The threshold for the searching results were: peptide confidence “high”; peptide number per protein >=1; and protein percolator score>=30.

(5) Computational methods

Density functional theory (DFT) computations were performed with *Gaussian 16* (**Supplementary Data 2**)[2]. Geometry optimizations were carried out using the hybrid functional B3LYP[3, 4] with the 6-311+G(d,p) basis set. Frequency calculations were carried out at the same level of theory to characterize the stationary points as local minima (no imaginary frequency) or transition states (one imaginary frequency), and to obtain thermal corrections at 298 K. Intrinsic reaction coordinate (IRC) calculations were performed to ensure all transition states connect the appropriate reactants and products. Various conformations were considered, with the lowest energy one reported for each structure. More accurate single-point energy calculations were calculated with the meta-hybrid GGA functional M06-2X[5] with the 6-311+G(d,p) basis set. All energies reported are enthalpies or Gibbs free energies obtained from single point calculations

at the M06-2X/6-311+G(d,p) level plus the thermodynamic corrections at the B3LYP/6-311+G(d,p) level of theory.

(6) General procedures for chemical synthesis, compound purification and characterization

Reagents were purchased from commercial sources and were used without further purification, unless otherwise noted. Analytical thin layer chromatography (TLC) was carried out on Silica Gel 60 Å F254 plates and visualized by UV light (254 nm) or treated with 10% 4- (Dimethylamino) cinnamaldehyde in ethanol (w/v) with gentle heating. Probes were purified by semi-preparative high performance liquid chromatography. Mass spectra of probes were collected with Q-Exactive Orbitrap mass spectrometer (Thermo Fisher). NMR spectra were recorded on Bruker Avance III 500 MHz with CryoProbe Prodigy at 500 MHz for ^{1}H NMR, and 126 MHz for ^{13}C NMR, respectively. Chemical shifts were reported in ppm with tetramethylsilane (TMS) as internal standard, and coupling constants (J) were given in Hertz. Signals for 1H NMR were recorded as follows: s (singlet), d (doublet), t (triplet) or m (multiplet). BP1, BP2, BP3, BP8, BP9, BN1 and BN2 were synthesized according to previous reports[6, 7].

(7) Chemical sysnthesis of biotin phenol derivatives

1) Synthesis of BP1

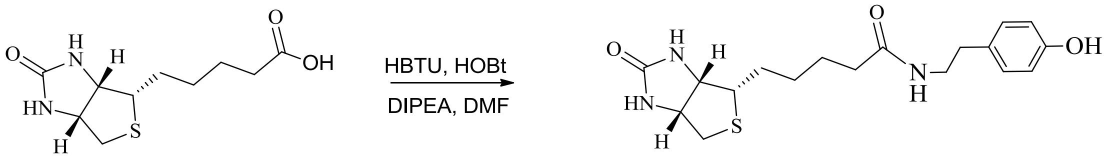

Biotin BP1

A mixture of biotin (2.44 g, 0.01 mol), O-Benzotriazole-N, N, N', N'-tetramethyl-uronium-hexafluorophosphate (HBTU, 4.17 g, 0.011 mol), 1-Hydroxybenzotriazole (HOBt, 1.49 g, 0.011 mol), and N, N-Diisopropylethylamine (DIPEA, 1.55 g, 0.012 mol) in N, N-Dimethylformamide (DMF, 20 ml) was stirred at room temperature. After stirring for 40min, 4-(2-aminoethyl) phenol (1.5 g, 0.011 mol) was added into the reaction pool, and the mixture was incubated overnight. Then the mixture was dried by evaporation. The crude product was separated on silica gel (dichloromethane (DCM):

methanol (MeOH) = 50:1~10:1) and recrystallized with MeOH to get the purified product BP1 (1.5 g, yield 41.3%). ^{1}H NMR (500 MHz, DMSO-d6) δ 9.18 (s, 1H), 7.82 (t, J = 5.5 Hz, 1H), 6.98 (d, J = 8.4 Hz, 2H), 6.67 (d, J= 8.4Hz, 2H), 6.44 (s, 1H), 6.37 (s, 1H), 4.31 (m, 1H), 4.12 (m, 1H), 3.20 - 3.16 (m, 2H), 3.08 (m, 1H), 2.84 - 2.81 (dd, J= 12.5 Hz, 5.1 Hz, 1H), 2.59 - 2.56 (m, 3H), 2.03 (t, J= 7.4 Hz, 2H), 1.63 - 1.41 (m, 4H), 1.34 - 1.22 (m, 2H). ^{13}C NMR (126 MHz, DMSO-d6) 172.37, 163.21, 156.03, 130.02, 129.93, 115.50, 61.50, 59.66, 55.91, 40.89, 35.67, 34.87, 28.66, 28.50, 25.78. HRMS (*m/z*): $[M+H]^+$ calculated for $C_{18}H_{26}N_3O_3S$ 364.1689, found 364.1683.

2) Synthesis of BP2

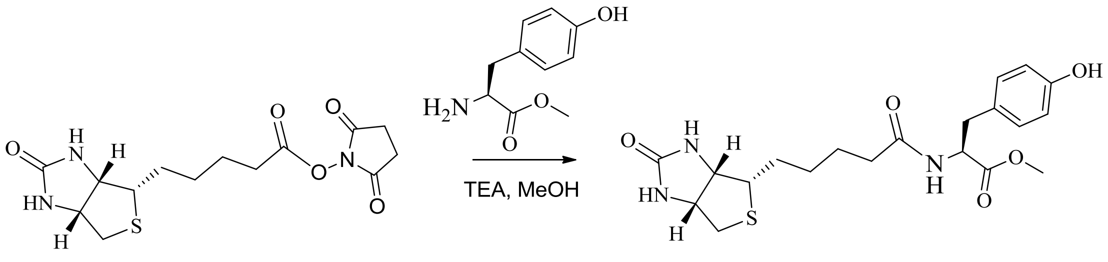

A mixture of biotin-osu (100 mg, 0.29 mmol), tyrosine methyl ester (68 mg, 0.35 mmol), Triethylamine (TEA, 35 mg, 0.35 mmol) in MeOH was stirred for 3 hours at room temperature. Then the mixture was evaporated to dryness. The crude product was chromatographed on silica gel (DCM:MeOH= 50:1~10:1) and was separated by semi-preparative column to get the purified product BP2 (40 mg, 32.5%). ^{1}H NMR (500 MHz, DMSO-d6) δ 9.25 (s, 1H), 8.23 (d, J = 7.8 Hz, 1H), 6.99 (d, J = 8.5 Hz, 2H), 6.66 (d, J = 8.5Hz, 2H), 6.41 (s, 1H),6.37 (s, 1H), 4.37 (m, 1H), 4.31 (m, 1H), 4.11 (m, 1H), 3.59 (s, 3H), 3.06 (m, 1H), 2.88 (m, 2H), 2.75 (m, 1H), 2.60 - 2.57 (d, J= 12.4 Hz, 1H), 2.06 (m, 2H), 1.61 - 1.39 (m, 4H), 1.28 – 1.18 (m, 2H). ^{13}C NMR (126 MHz, DMSO-d6) 172.84, 172.70, 163.19, 156.37, 130.41, 127.74, 115.45, 61.47, 59.65, 55.88, 54.24, 52.22, 36.42, 35.17, 28.44, 25.61. HRMS (*m/z*): $[M+H]^+$ calculated for $C_{20}H_{28}N_3O_5S$ 422.1744, found 422.1742.

3) Synthesis of BP3

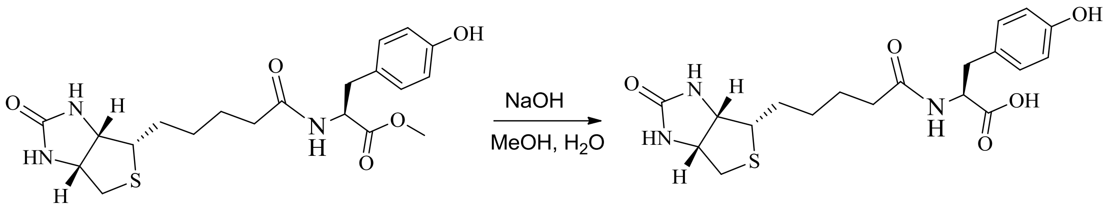

BP2 BP3

A solution of BP2 (96 mg, 0.23 mmol) in MeOH/ water (9 ml, 2:1, v/v) was added to sodium hydroxide (27.4 mg, 0.69 mmol) at 0 ℃. After stirring for 6 h, dilute HCl (1 M) was added to adjust pH equal to 7. The mixture was filtered, and the filter cake was separated by semi-preparative column to get the purified BP3 (50 mg, 53.8%). ^{1}H NMR (500 MHz, DMSO-d6) δ 9.22 (s, 1H), 8.06 (d, J= 8.1 Hz, 1H), 7.01 (d, J = 8.4 Hz, 2H), 6.66 (d, J= 8.4 Hz, 2H), 6.41 (s, 1H), 6.437 (s, 1H), 4.33 (m, 2H), 4.11 (m, 1H), 3.05 (m, 1H), 2.94 - 2.90 (dd, J= 13.9 Hz, 4.8 Hz, 1H), 2.85 - 2.82 (dd, J= 12.5Hz, 5.1 Hz, 1H), 2.74 - 2.69 (dd, J= 13.9 Hz, 9.7 Hz, 1H), 2.59 (d, J= 12.4 Hz, 1H), 2.06 (m, 2H), 1.62 – 1.38 (m, 4H), 1.28 - 1.17 (m, 2H). ^{13}C NMR (126 MHz, DMSO-d6), 173.84, 172.56, 163.20, 156.27, 130.43, 128.21, 115.38, 61.46, 59.65, 55.88, 54.10, 36.46, 35.27, 28.44, 25.64. HRMS (*m/z*): $[M+H]^+$ calculated for $C_{19}H_{26}N_3O_5S$ 408.1588, found 408.1586.

4) Synthesis of BP4

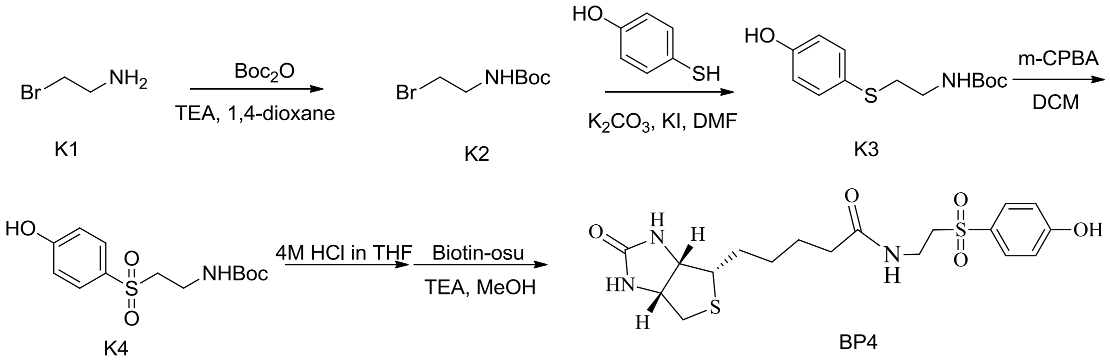

K1 K2 K3 K4 BP4

Synthesis of K2:

A mixture of 2-bromoethanamine hydrobromide (2.25 g, 0.011 mol), triethylamine (1.51 g, 0.015 mol) in 1, 4-dioxane (20 ml) was stirred at 0 ℃. Di-tert-butyl pyrocarbonate (2.62g, 0.012mol) was added to the mixture with several portions. 12 h later, the mixture was evaporated to dryness, and dissolved in 50 ml EA. The organic layer was washed with saturated Ammonium chloride solution (50 ml), saturated

sodium bicarbonate (50 ml), saturated sodium chloride solution (50 ml). Then the organic layer was dried under vacuum to get the compound K2 (2.1 g, 93.7%). K2 was used in the next step without further purification.

Synthesis of K3:

A mixture of 4-mercaptophenol (1.42 g, 11.24 mmol), K_2CO_3 (1.5 g, 11.24 mmol), KI (1.86 g, 11.24 mmol) in DMF (10 ml) was stirred at 0 ℃ for 6 h. Then, K2 (2.1 g, 9.37 mmol) was added to the mixture. After 12 h, the mixture was evaporated to dryness, and dissolved in DCM (50 ml). The organic layer was washed with saturated sodium chloride solution (50 ml), and dried over Na_2SO_4. The crude product was chromatographed on silica gel (petroleum ether (PE): ethyl acetate (EA) = 30:1~ 6:1) to get the compound K3 (1.7 g, 67.4%).

Synthesis of K4:

A solution of K3 (1.71 g, 6.36 mmol) in DCM (30 ml) was added to 3-Chloroperbenzoic acid (5.8 g, 28.6 mmol) at 0 ℃. After stirring for 12 h, the mixture was filtered, and the filter cake was used in the next step without further purification (2.13 g, 67.4%).

Synthesis of BP4:

K4 (2.13 g, 7.08 mmol) was dissolved in THF (20 ml), and diluted HCl (4 mol/L, 18 ml, 70.8 mmol) was added. After stirring for 12 h, the mixture was evaporated to dryness, and dissolved in MeOH (5 ml). A solution of Biotin-osu (2.9 g, 8.5 mmol) in MeOH (5 ml) was added to the mixture. After stirring for 6 h, the mixture was evaporated to dryness, and was chromatographed by silica gel (DCM:MeOH = 50:1~5:1). The crude product was recrystallized with MeOH to get the product BP4 (500 mg, 16.5%). ^{1}H NMR (500 MHz, DMSO-d6) δ 10.68 (s, 1H), 7.87 (t, J = 5.4 Hz, 1H), 7.71 - 7.69 (m, 2H), 6.97 - 6.95 (m, 2H), 6.42 (s, 1H), 6.37 (s, 1H), 4.30 (m, 1H), 4.12 (m, 1H), 3.32 - 3.25 (m, 4H), 3.08 (m, 1H), 2.81 (dd, J= 12.5 Hz, 5 Hz, 1H), 2.58 - 2.56 (d, J= 12.4 Hz, 1H), 1.95 (t, J = 7.3 Hz, 2H), 1.61 - 1.36 (m, 4H), 1.32 - 1.18 (m, 2H). ^{13}C NMR (126 MHz, DMSO-d6) δ 172.66, 163.19, 162.68, 130.64, 129.26, 116.29, 61.47, 59.65, 55.83, 54.73, 35.44, 33.66, 28.60, 28.45, 25.44. HRMS (*m/z*): $[M+H]^+$ calculated for $C_{18}H_{26}N_3O_5S_2$ 428.1308, found 428.1304.

5) Synthesis of BP5

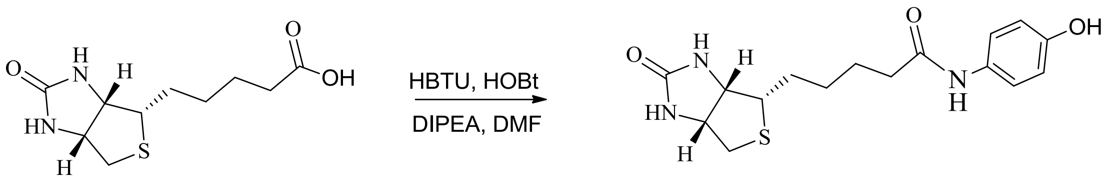

Biotin BP5

A mixture of biotin (2.44 g, 0.01 mol), HBTU (4.17g, 0.011 mol), HOBT (1.49g, 0.011 mol), DIPEA (1.55 g, 0.012 mol) in DMF (20 ml) was stirred at room temperature. After 40 min, 4-aminophenol (1.5 g, 0.011 mol) was added, and the mixture was stirred overnight. Then the mixture was evaporated to dryness. The crude product was chromatographed on silica gel (DCM:MeOH = 50:1~10:1) and recrystallized with MeOH to get the purified product BP5 (0.7 g, yield 20.9%). ^{1}H NMR (500 MHz, DMSO-d6) δ 9.60 (s, 1H), 9.13 (s, 1H), 7.35 (d, J = 8.8 Hz, 2H), 6.67 (d, J = 8.8 Hz, 2H), 6.45 (s, 1H), 6.37 (s, 1H), 4.31 (m, 1H), 4.14 (m, 1H), 3.14 (m, 1H), 2.84 (dd, J = 17.5 Hz, 5.1Hz, 1H), 2.59 (d, J = 12.4 Hz, 1H), 2.25 (t, J = 14.8 Hz, 2H), 1.69 - 1.46 (m, 4H), 1.42 - 1.27 (m, 2H). ^{13}C NMR (126 MHz, DMSO-d6) 170.91, 163.22, 153.55, 131.487, 121.33, 115.44, 61.53, 59.67, 55.90, 36.52, 28.75, 28.56, 25.73. HRMS (m/z): $[M+H]^+$ calculated for $C_{16}H_{22}N_3O_3S$ 336.1376, found 336.1375.

6) Synthesis of BP6

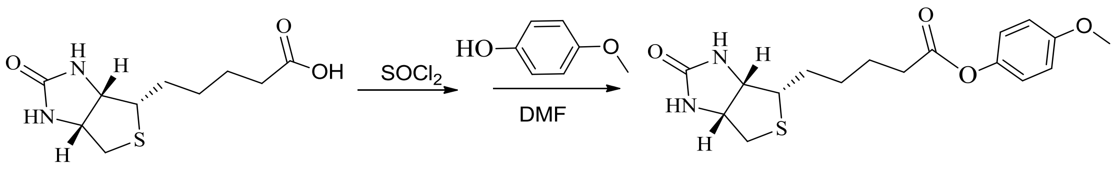

Biotin L1

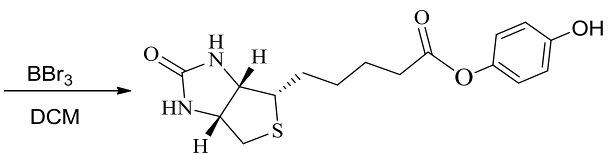

BP6

Synthesis of L1:

Biotin (2.44 g, 0.01 mol) was added to the solvent sulfur dichloride (50 ml) with several portions at 0 ℃. After stirring for 6 h, the mixture was evaporated to dryness, and dissolved in 50 ml acetonitrile (MeCN). DIPEA (2.58 g, 0.02 mol) was added to the mixture, followed by a solution of 4-methoxyphenol (3.3g, 0.03 mol) in MeCN (10 ml).

After stirring for 6 h, the mixture was evaporated to dryness. The crude product was chromatographed on silica gel (DCM:MeOH = 30:1~20:1) to get the purified product L1 (1.44 g, yield 41.1%).

Synthesis of BP6:

L1 (1.44 g, 4.11 mmol) was dissolved in DCM (50 ml). BBr3 (1.9 ml, 20.1 mmol) was added to the mixture dropwise at 0 ℃. After stirring for 12 h, water (100 ml) was added to the mixture at 0 ℃. The mixture was filtered, and the filter cake was separated by semi-preparative column to get the purified product BP6 (0.8 g, yield 58.0%). ^{1}H NMR (500 MHz, DMSO-d6) δ 9.44 (s, 1H), 6.90 – 6.88 (m, 2H), 6.76 – 6.74 (m, 2H), 6.47 (s, 1H), 6.38 (s, 1H), 4.31 (m, 1H), 4.15 (m, 1H), 3.13 (m, 1H), 2.83 (dd, J = 12.4, 5.1 Hz, 1H), 2.58 (d, J = 12.4 Hz, 1H), 2.53 (m, 2H), 1.71 - 1.47 (m, 4H), 1.45 - 1.34 (m, 2H). ^{13}C NMR (126 MHz, DMSO-d6) δ 172.58, 163.19, 155.39, 143.05, 122.88, 116.00, 61.49, 59.65, 55.79, 33.72, 28.46, 28.41, 24.91. HRMS (m/z): $[M+H]^+$ calculated for $C_{16}H_{21}N_2O_4S$ 337.1217, found 337.1207.

7) Synthesis of BP7

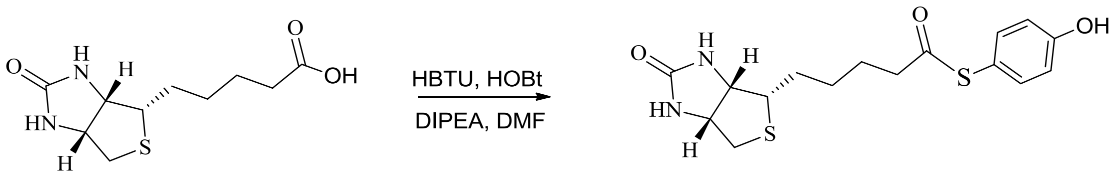

Biotin BP7

A mixture of biotin (2.44 g, 0.01 mol), HBTU (4.17g, 0.011 mol), HOBT (1.49g, 0.011 mol), DIPEA (1.55 g, 0.012 mol) in DMF (20 ml) was stirred at room temperature. After 40 min, 4-mercaptophenol (1.39 g, 0.011 mol) was added, and the mixture was incubated overnight. Then the mixture was evaporated to dryness. The crude product was chromatographed on silica gel (DCM:MeOH = 50:1~10:1) and recrystallized with MeOH to give the purified product BP7(2 g, yield 56.8%). ^{1}H NMR (500 MHz, DMSO-d6) δ 9.94 (s, 1H), 7.20 – 7.18 (m, 2H), 6.84 – 6.82 (m, 2H), 6.45 (s, 1H), 6.37 (s, 1H), 4.31 (m, 1H), 4.14 (m, 1H), 3.10 (m, 1H), 2.82 (dd, J = 12.4, 5.1 Hz, 1H), 2.64 (t, J = 7.4 Hz, 2H), 2.59 (d, J = 12.4 Hz, 1H), 1.66 – 1.43 (m, 4H), 1.41 – 1.29 (m, 2H). ^{13}C NMR (126 MHz, DMSO-d6) δ 198.51, 163.18, 159.21, 136.68, 116.78, 116.40, 61.47,

59.64, 55.75, 42.88, 28.42, 28.26, 25.49. HRMS (m/z): $[M+H]^+$ calculated for $C_{16}H_{21}N_2O_3S_2$ 353.0988, found 353.0986.

8) Synthesis of BP8

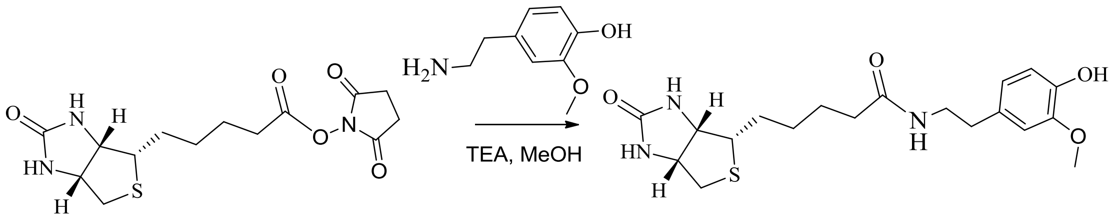

Biotin-osu BP8

A mixture of biotin-osu (100 mg, 0.29 mmol), TEA (35 mg, 0.35 mmol), 4-(2-aminoethyl)-2-methoxyphenol (58.4 mg, 0.35 mmol) in MeOH (5 ml) was stirred for 3 hours at room temperature. Then the mixture was evaporated to dryness. The crude product was chromatographed on silica gel (DCM:MeOH = 50:1~10:1) and was separated by semi-preparative column to get the purified product BP8 (40 mg, 33.5%). ^1H NMR (500 MHz, DMSO-d6) δ 8.73 (s, 1H), 7.83 (t, J = 5.6 Hz, 1H), 6.75 (m, 1H), 6.67 (m, 1H), 6.57 (m, 1H), 6.44 (s, 1H), 6.38 (s, 1H), 4.32 (m, 1H), 4.13 (m, 1H), 3.75 (s, 3H), 3.22 (m, 2H), 3.08 (m, 1H), 2.83 (dd, J = 12.4, 5.1 Hz, 1H), 2.59 (m, 3H), 2.05 (t, J = 7.3 Hz, 2H), 1.65 – 1.41 (m, 4H), 1.37 – 1.21 (m, 2H). ^{13}C NMR (126 MHz, DMSO-d6) δ 172.39, 163.21, 147.79, 145.17, 130.73, 121.17, 115.73, 113.14, 61.50, 59.66, 55.94, 55.90, 40.80, 35.69, 35.27, 28.66, 28.51, 25.81. HRMS (m/z): $[M+H]^+$ calculated for $C_{19}H_{28}N_3O_4S$ 394.1795, found 394.1792.

9) Synthesis of BP9

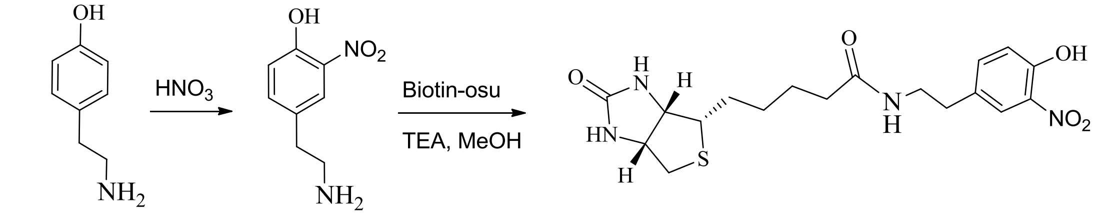

4-(2-aminoethyl)phenol M1 BP9

Synthesis of M1:

A concentrated nitric acid (60% conc, 10g, 0.096 mol) was added dropwise to a powder

of 4-(2-aminoethyl)phenol (2.74g, 0.02 mol) in an ice bath. After stirring for 6h, the mixture was then diluted with 20ml water. The resulted solution was separated with a cation exchange resin (eluted with 2mol/L HCl(aq)) to give M1 (1.2g, yield 27.5%). The product was used directly in the next step without further purification.

Synthesis of BP9:

A mixture of biotin-osu (100 mg, 0.29 mmol), 4-(2-aminoethyl)-2-nitrophenol (76 mg, 0.35 mmol), TEA (35 mg, 0.35 mmol) in MeOH was stirred for 3h at room temperature. Then the mixture was evaporated to dryness. The crude product was chromatographed on silica gel (DCM:MeOH = 50:1~10:1) and was separated by semi-preparative column to get the purified prodcut BP9(60 mg, 50.8%). ^{1}H NMR (500 MHz, DMSO-d6) δ 7.84 (t, J = 5.7 Hz, 1H), 7.71 (m, 1H), 7.37 (m, 1H), 7.04 (m, 1H), 6.43 (s, 1H), 6.37 (s, 1H), 4.31 (m, 1H), 4.11 (m, 1H), 3.25 (m, 3H), 3.07 (m, 1H), 2.82 (dd, J = 12.4, 5.1 Hz, 1H), 2.67 (t, J = 7.0 Hz, 2H), 2.57 (d, J = 12.4 Hz, 1H), 2.02 (t, J = 7.4 Hz, 2H), 1.61 – 1.39 (m, 4H), 1.30 – 1.20 (m, 2H). ^{13}C NMR (126 MHz, DMSO-d6) δ 172.50, 163.20, 151.23, 136.70, 136.36, 131.07, 125.19, 119.56, 61.47, 59.65, 55.87, 35.65, 34.05, 28.60, 28.45, 25.75. HRMS (m/z): $[M+H]^+$ calculated for $C_{18}H_{25}N_4O_5S$ 409.1540, found 409.1536.

10) Synthesis of BP10

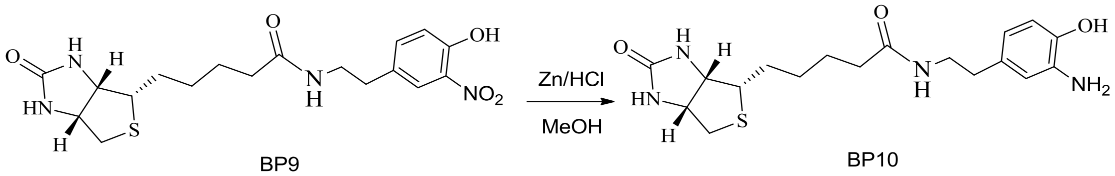

A mixture of BP9 (120 mg, 0.29 mmol) and zinc powder (286 mg, 4.35 mmol) in MeOH (5 ml) was incubated at 0 ℃. Diluted hydrochloric acid (3ml, 1 mol/L, 2.9 mmol) was added to the mixture dropwise. After stirring for 6 h, the mixture was filtered. The filtrate was evaporated to dryness, and was separated by semi-preparative column to get the purified product BP10 (50 mg, yield 45%). ^{1}H NMR (500 MHz, DMSO-d6) δ 8.80 (s, 1H), 7.82 (s, 1H), 6.53 (m, 1H), 6.42 (m, 2H), 6.37 (m, 1H), 6.20 (m, 1H), 4.45 (s, 2H), 4.31 (m, 1H), 4.13 (m, 1H), 3.12 (m, 3H), 2.82 (dd, J = 12.5, 5.1 Hz, 1H), 2.56 (d, J = 12.5 Hz, 1H), 2.45 (t, J = 7.6 Hz, 2H), 2.04 (t, J = 7.4 Hz, 2H), 1.64 – 1.41 (m,

4H), 1.34 – 1.23 (m, 2H). ^{13}C NMR (126 MHz, DMSO-d6) δ 172.31, 163.21, 142.83, 136.72, 130.64, 116.88, 115.21, 114.66, 61.49, 59.66, 55.88, 41.07, 35.69, 35.48, 28.65, 28.49, 25.77. HRMS (m/z): $[M+H]^+$ calculated for $C_{18}H_{27}N_4O_3S$ 379.1798, found 379.1796.

11) Synthesis of BN1

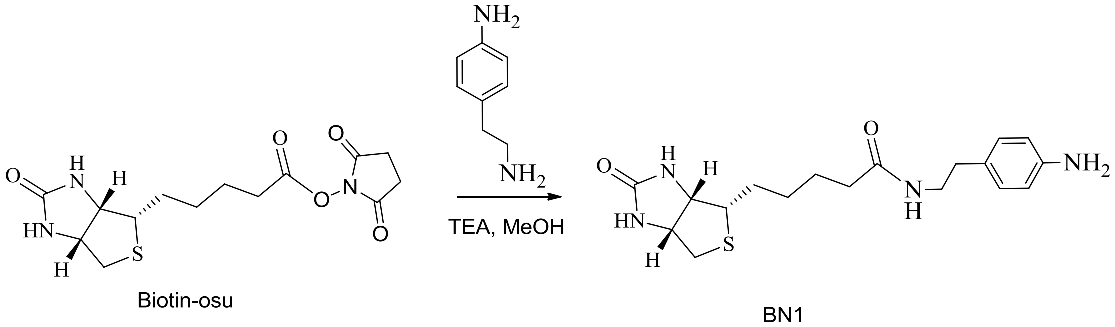

A mixture of biotin-osu (750.2 mg, 2.2 mmol), 4-(2-aminoethyl) aniline (272 mg, 2 mmol), TEA (606 mg, 6 mmol) in DMF was stirred for 3 hours at room temperature. Then the mixture was evaporated to dryness. The crude product was chromatographed on silica gel (DCM:MeOH = 50:1~5:1) and was separated by semi-preparative column to get the purified product BN1 (400 mg, 55.2%). ^1H NMR (500 MHz, DMSO-d6) δ 7.79 (t, J = 5.6 Hz, 1H), 6.86 (m, 2H), 6.52 (m, 2H), 6.43 (s, 1H), 6.37 (s, 1H), 5.26 (s, 2H), 4.31 (m, 1H), 4.13 (m, 1H), 3.15 (m, 2H), 3.08 (m, 1H), 2.82 (dd, J = 12.4, 5.1 Hz, 1H), 2.57 (m, 1H), 2.03 (t, J = 7.4 Hz, 2H), 1.65 – 1.42 (m, 4H), 1.36 – 1.21 (m, 2H). ^{13}C NMR (126 MHz, DMSO-d6) δ 172.25, 163.16, 129.48, 114.92, 61.49, 59.64, 55.90, 41.02, 35.68, 35.01, 28.66, 28.51, 25.79. HRMS (*m/z*): $[M+H]^+$ calculated for $C_{18}H_{27}N_4O_2S$ 363.1849, found 363.1838.

12) Synthesis of BN2

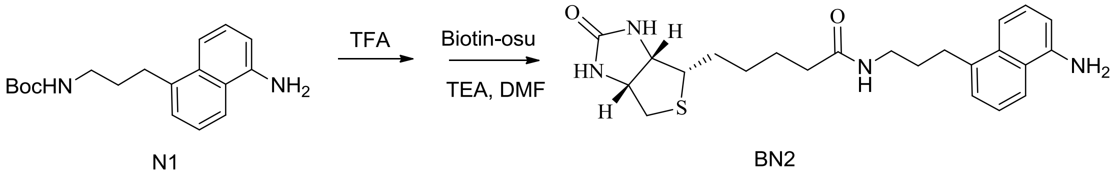

N1 (400 mg, 1.33 mmol) was dissolved in DCM (10 ml), and TFA (1ml, 13.4 mmol) was added dropwise at 0 ℃. After stirring for 3 h, the mixture was evaporated to dryness, and dissolved in DMF (10 ml). TEA was added to adjust pH equal to 8, followed by a solution of biotin-osu (500 mg, 1.46 mol) in DMF (5 ml). After stirring

for 3 h, the mixture was evaporated to dryness, and was chromatographed by silica gel (DCM:MeOH = 50:1~5:1). The crude product was separated by semi-preparative column to get the purified product BN2 (230 mg, 40.5%).

^{1}H NMR (500 MHz, DMSO-d6) δ 7.90 (m, 2H), 7.33 (m, 4H), 6.87 (m, 1H), 6.35 (m, 2H), 4.27 (m, 1H), 4.11 (m, 1H), 3.12 (m, 3H), 2.97 (m, 2H), 2.78 (dd, J = 12.4, 5.1 Hz, 1H), 2.55 (d, J = 12.4 Hz, 1H), 2.08 (t, J = 7.4 Hz, 2H), 1.77 (m, 2H), 1.65 – 1.43 (m, 4H), 1.38 – 1.24 (m, 2H). ^{13}C NMR (126 MHz, DMSO-d6) δ 172.40, 163.15, 126.80, 120.99, 61.48, 59.62, 55.89, 38.82, 35.74, 30.81, 30.65, 28.70, 28.51, 25.86. HRMS (*m/z*): [M+H]$^{+}$ calculated for $C_{23}H_{31}N_{4}O_{2}S$ 427.2162, found 427.2162.

(8) NMR spectra of the probes

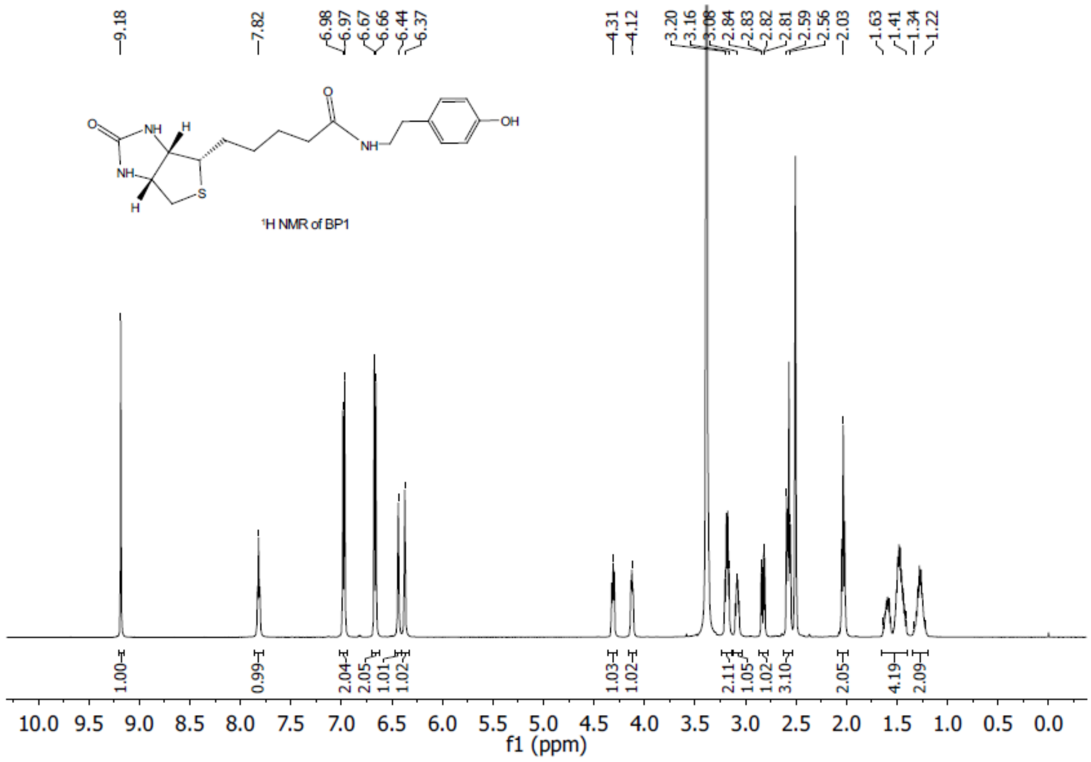

^{1}H NMR spectra of BP1

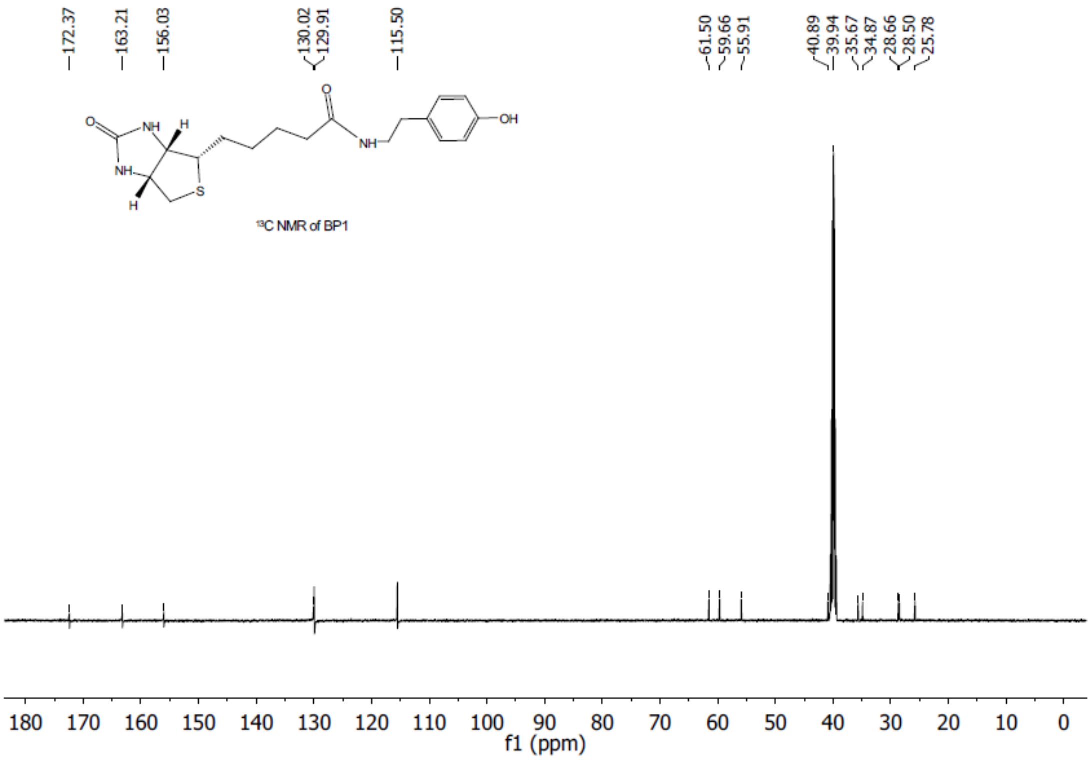

^{13}**C NMR spectra of BP1**

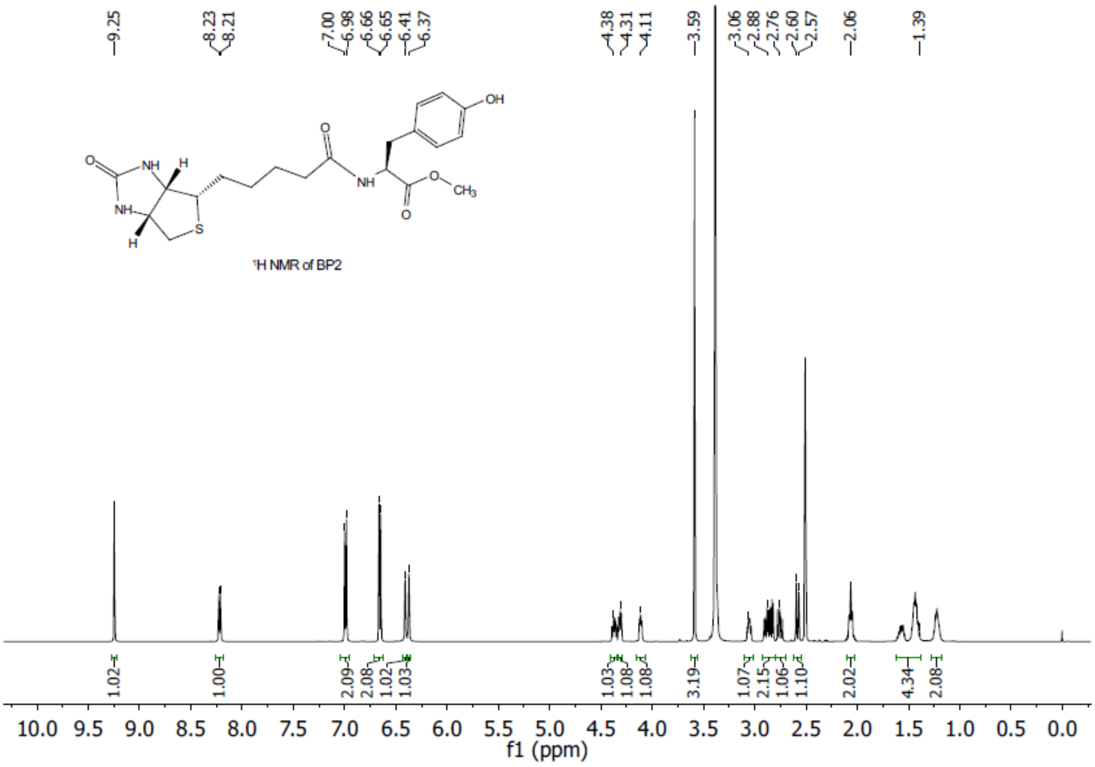

^{1}**H NMR spectra of BP2**

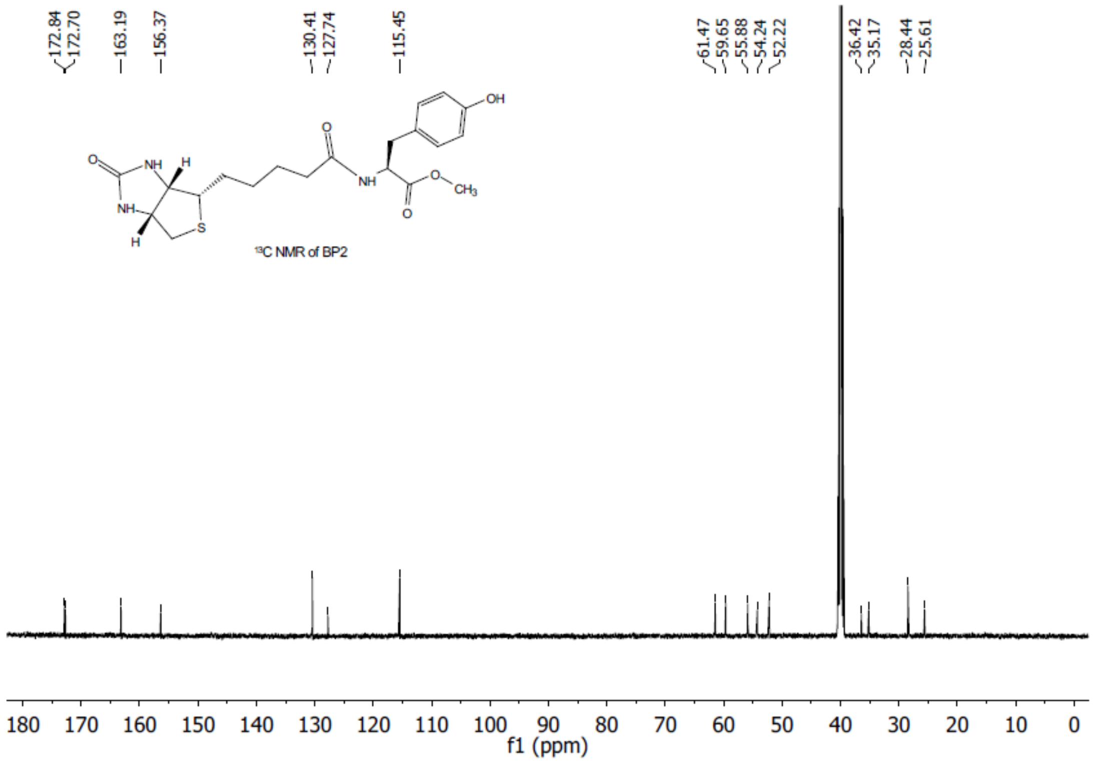

^{13}C NMR spectra of BP2

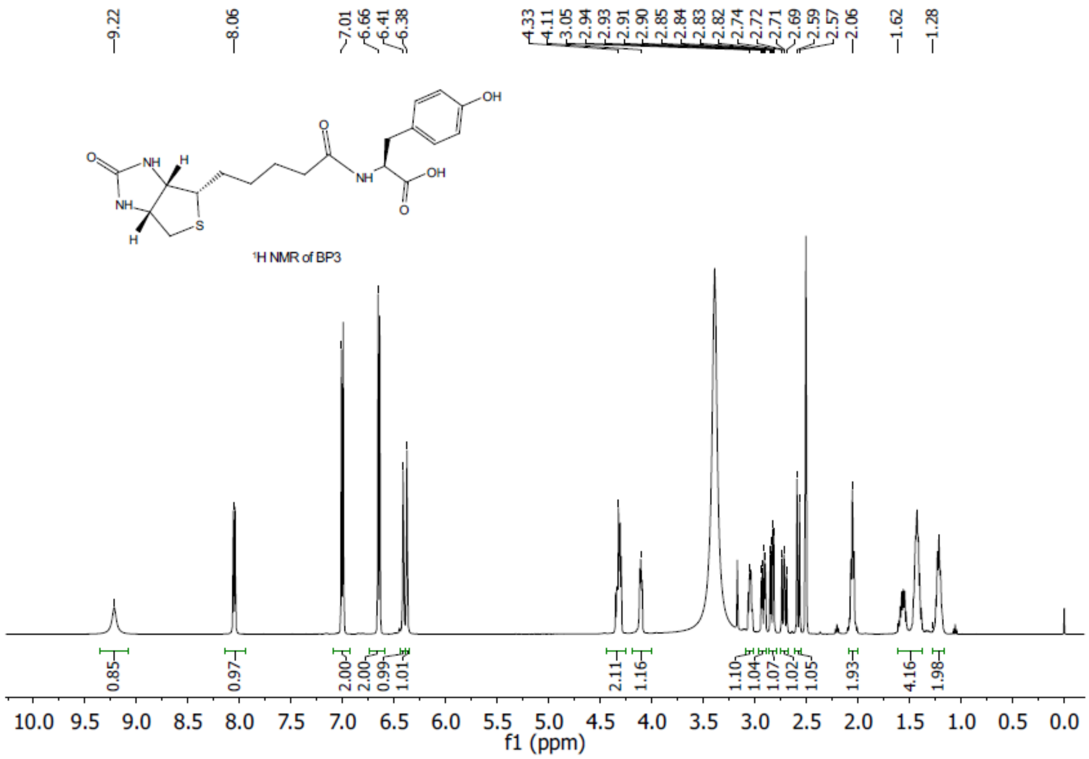

^{1}H NMR spectra of BP3

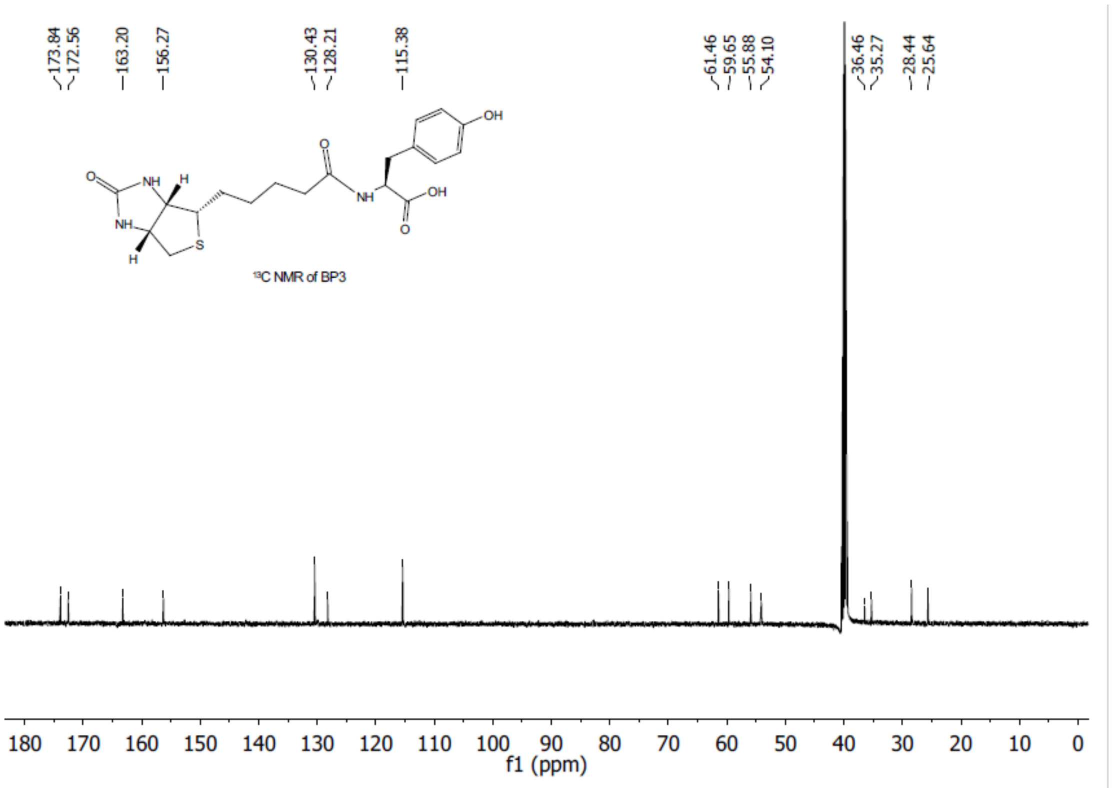

^{13}C NMR spectra of BP3

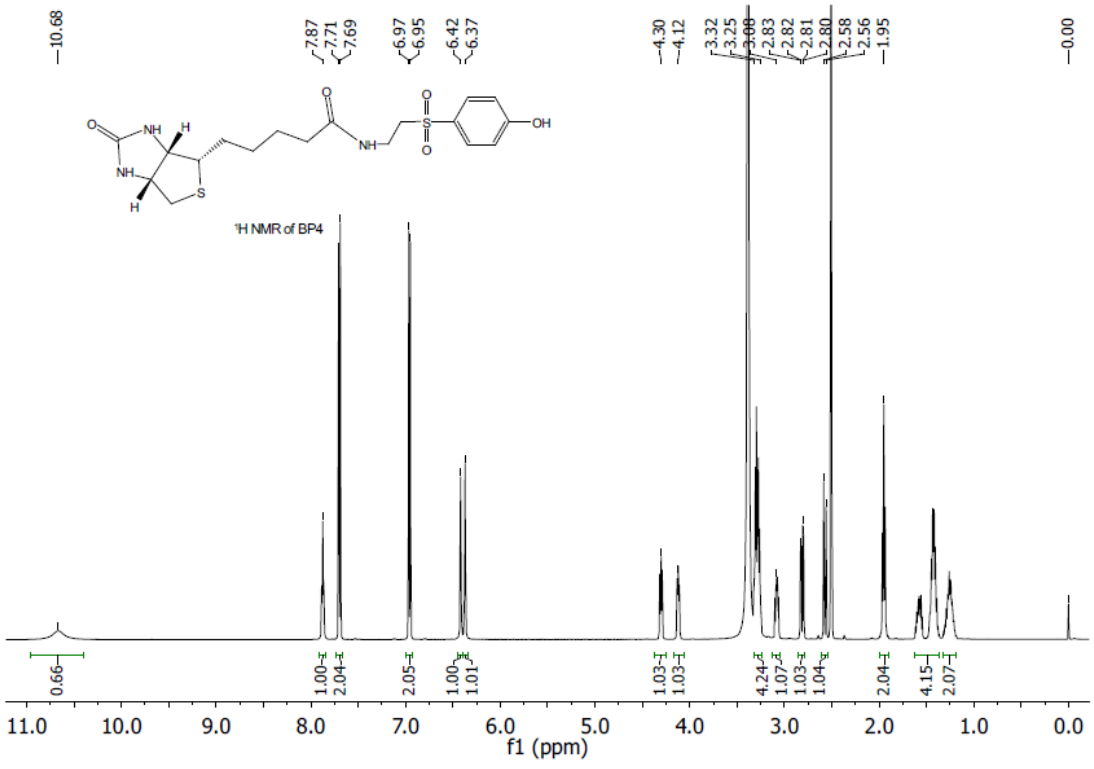

^{1}H NMR spectra of BP4

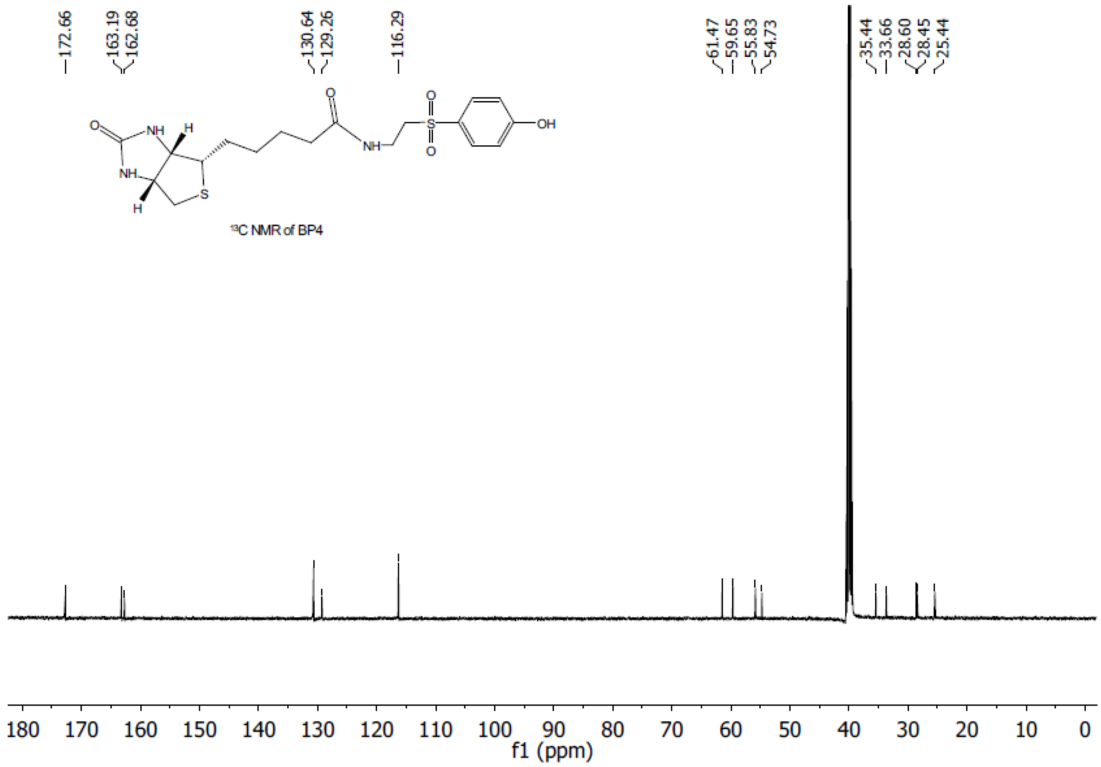

^{13}C NMR spectra of BP4

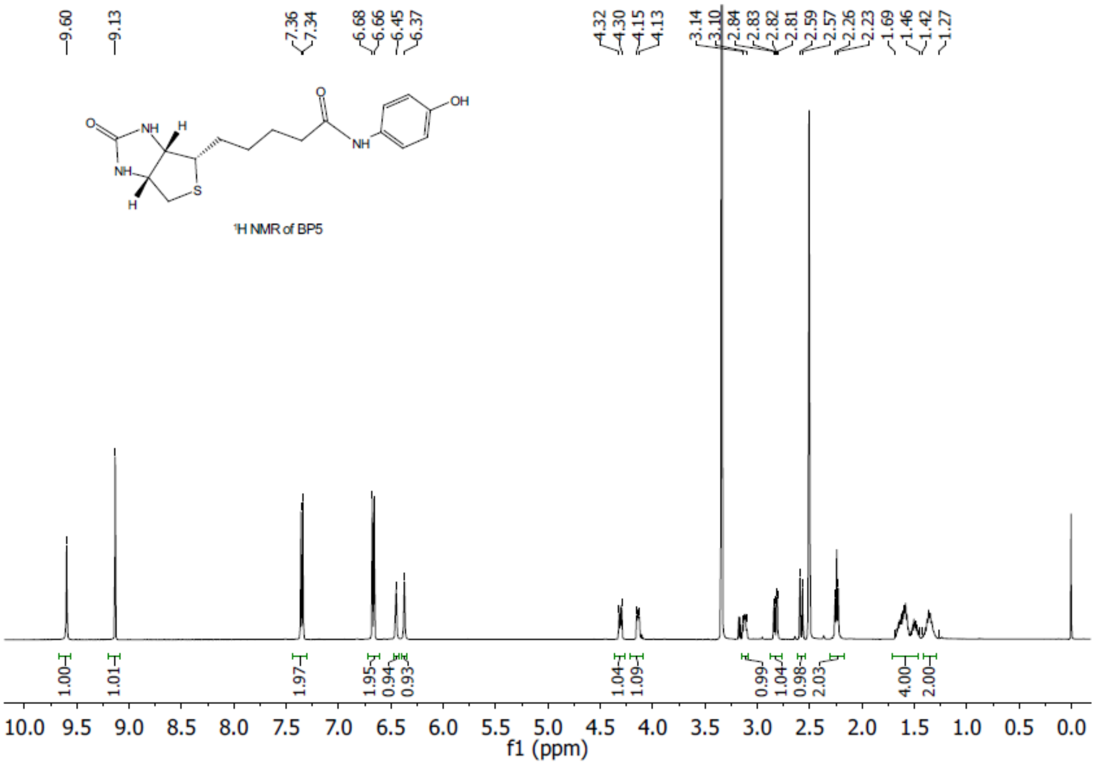

^{1}H NMR spectra of BP5

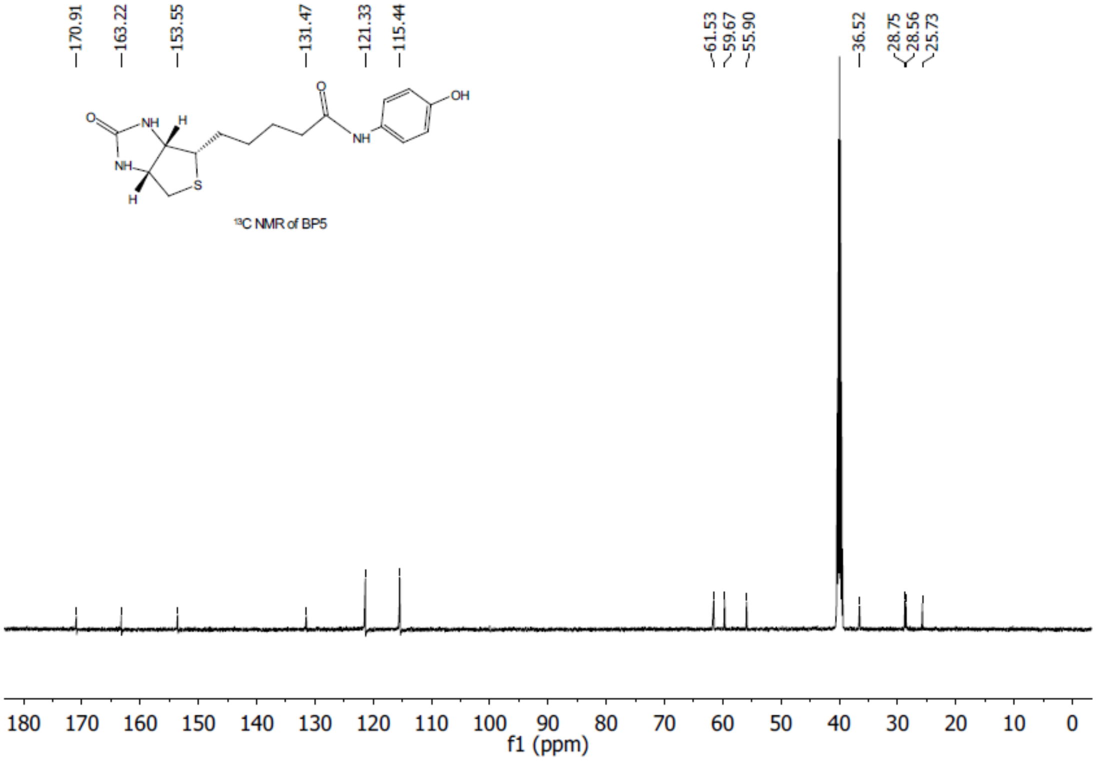

^{13}C NMR spectra of BP5

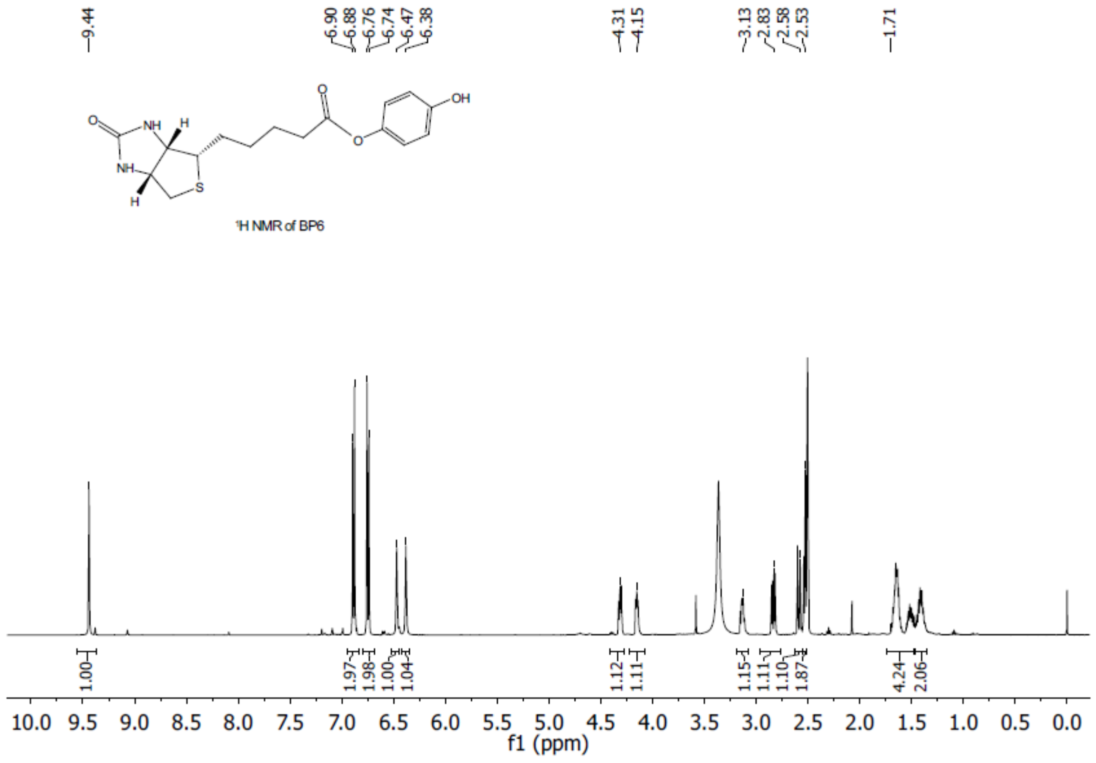

^{1}H NMR spectra of BP6

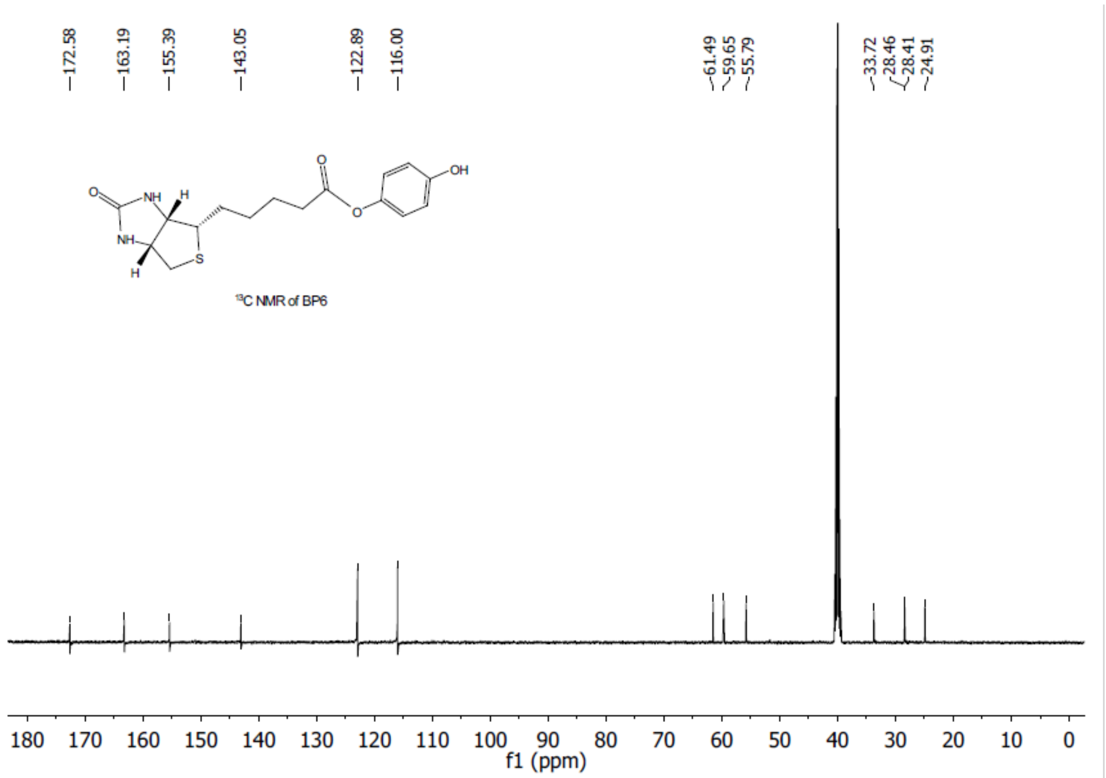

^{13}C NMR spectra of BP6

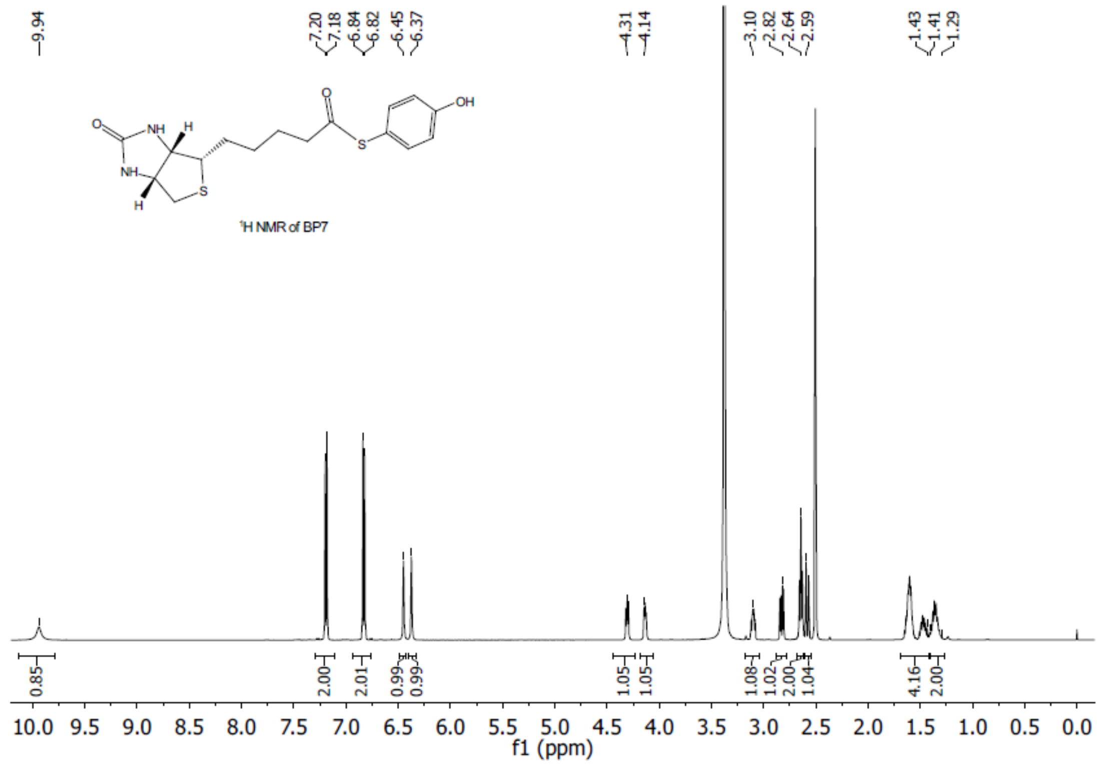

^{1}H NMR spectra of BP7

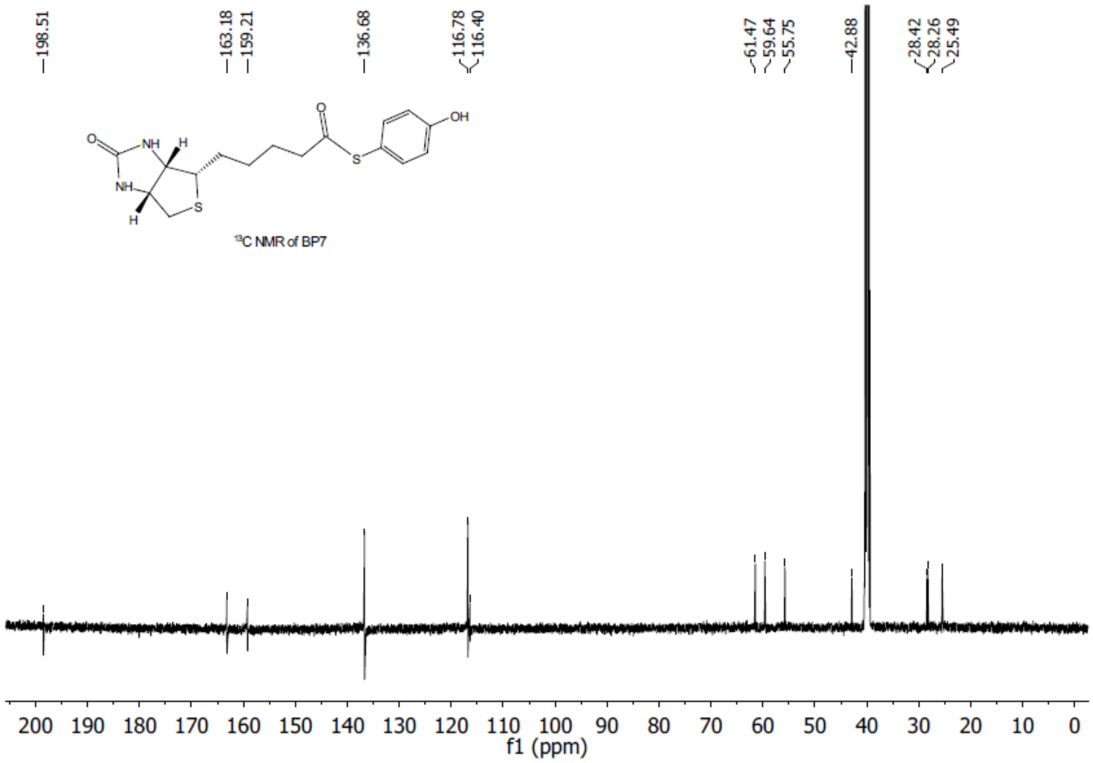

^{13}C NMR spectra of BP7

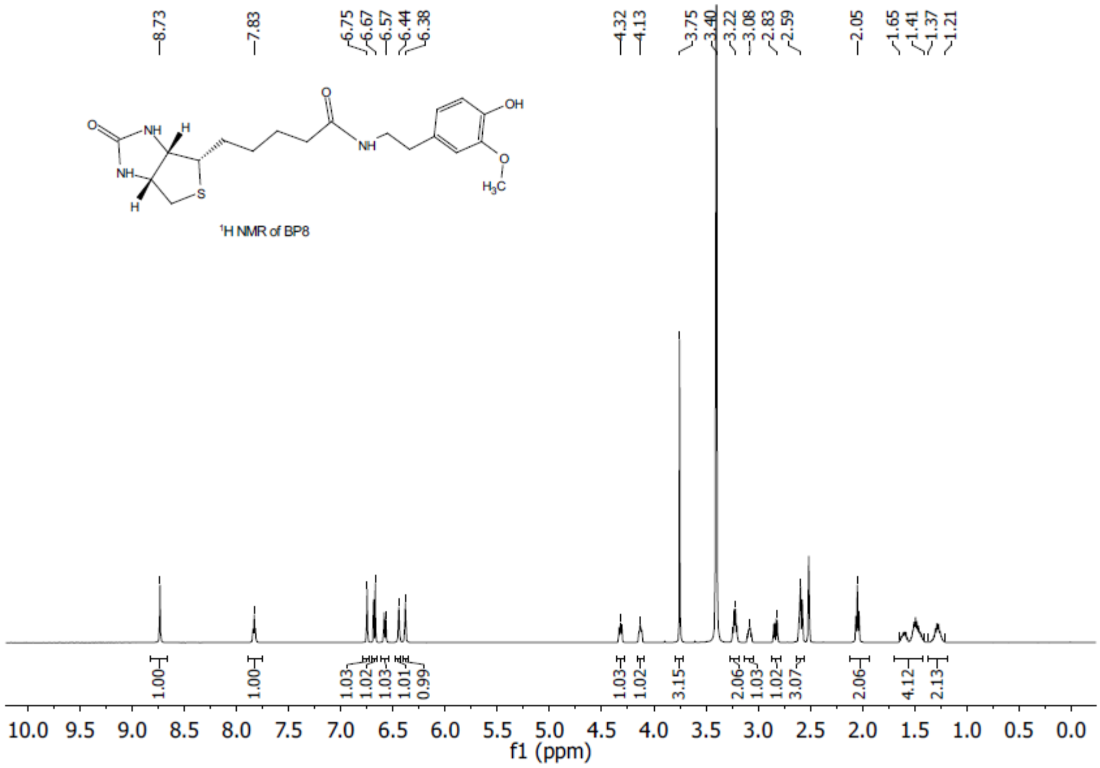

^{1}H NMR spectra of BP8

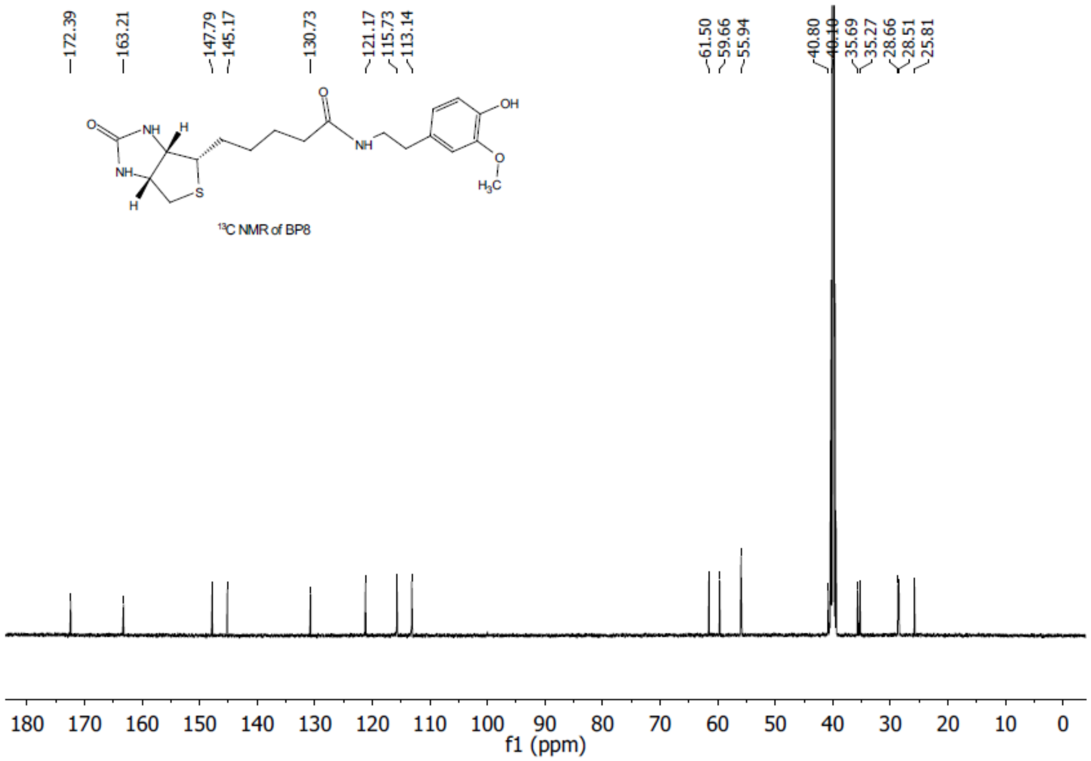

^{13}C NMR spectra of BP8

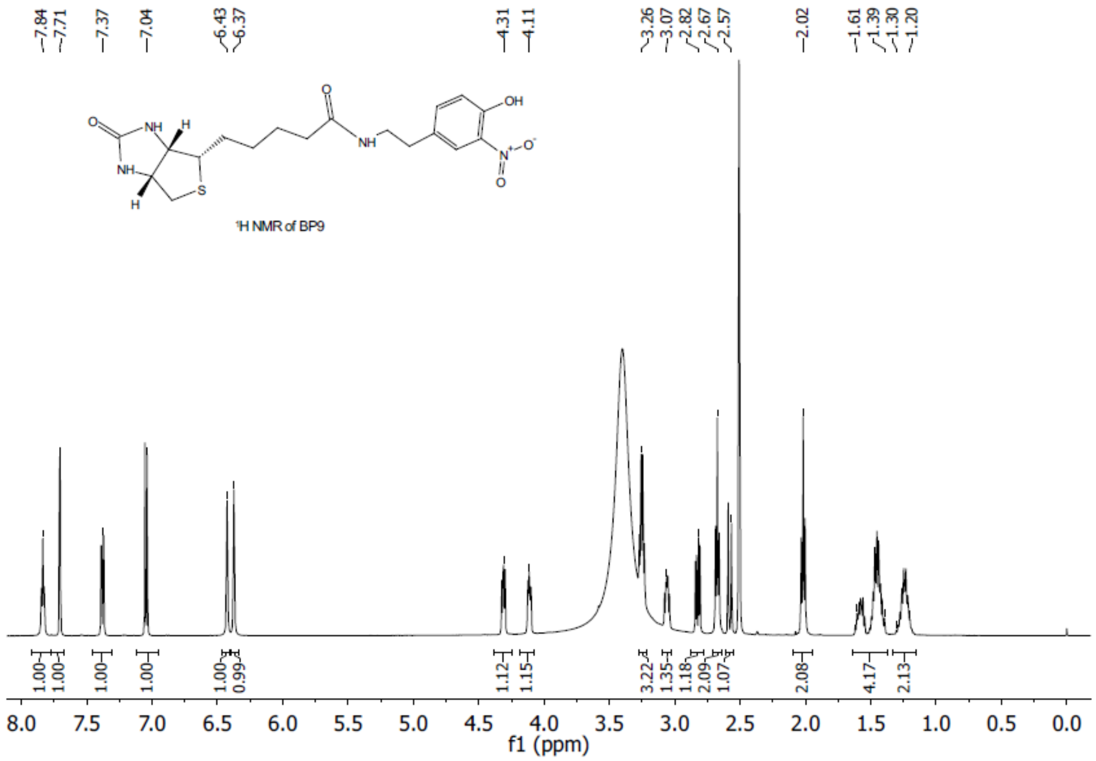

^{1}H NMR spectra of BP9

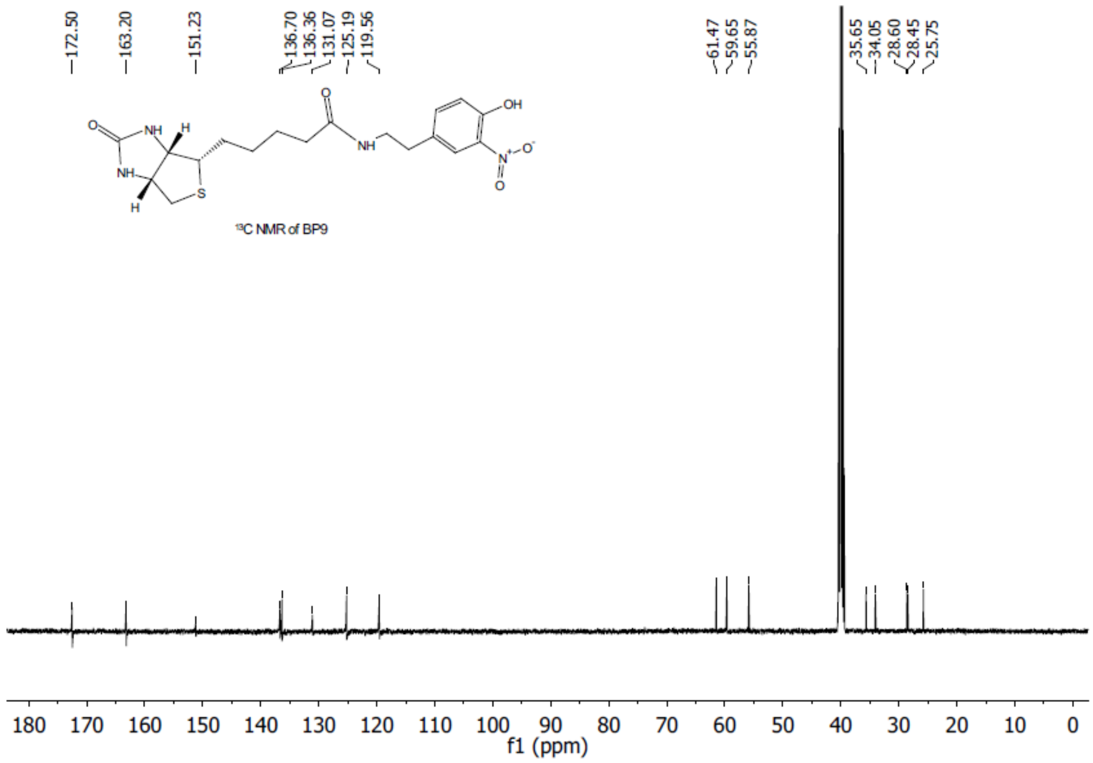

^{13}C NMR spectra of BP9

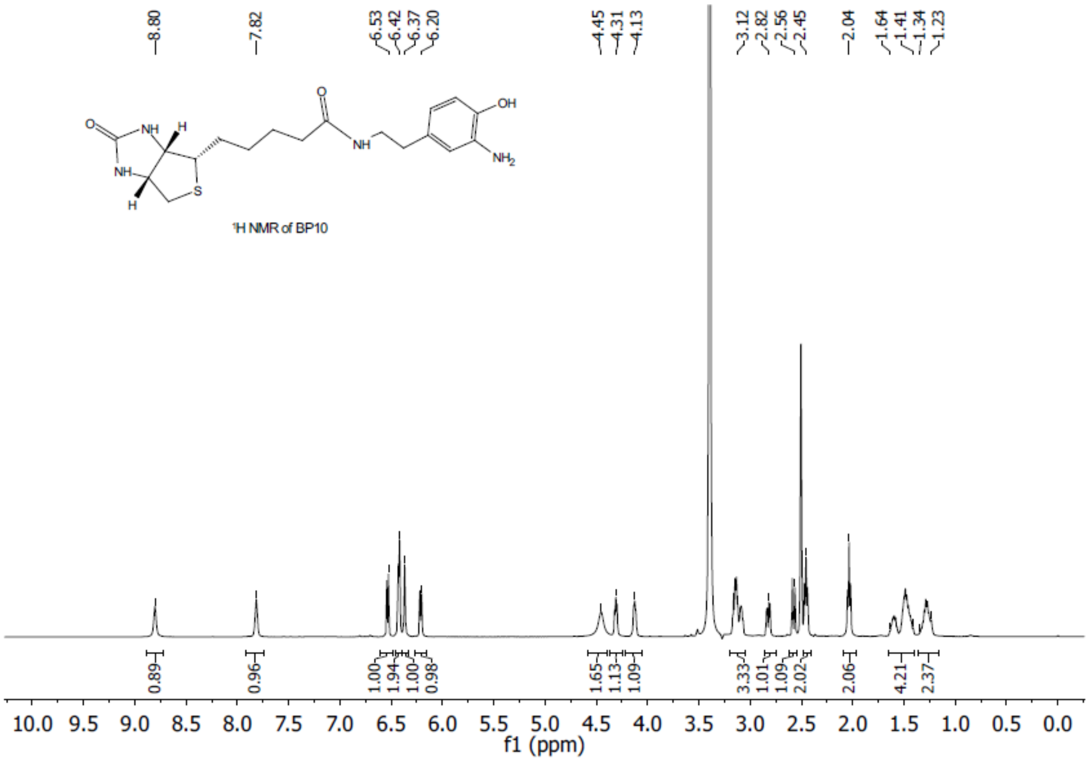

^{1}H NMR spectra of BP10

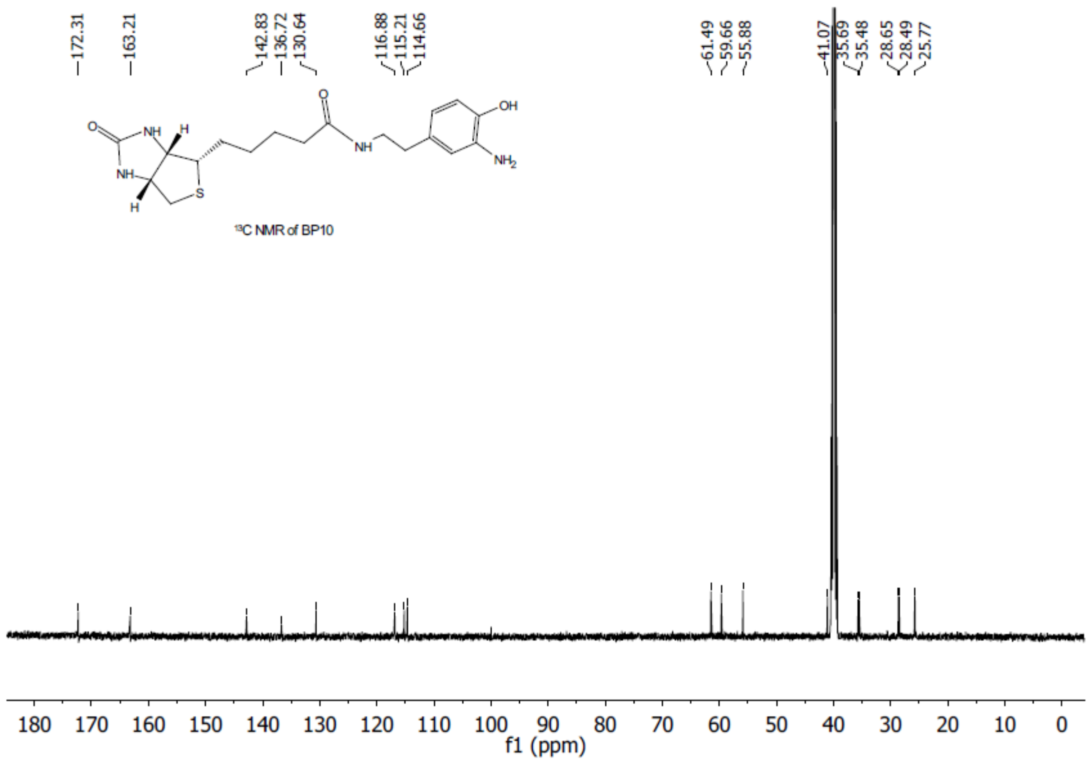

^{13}C NMR spectra of BP10

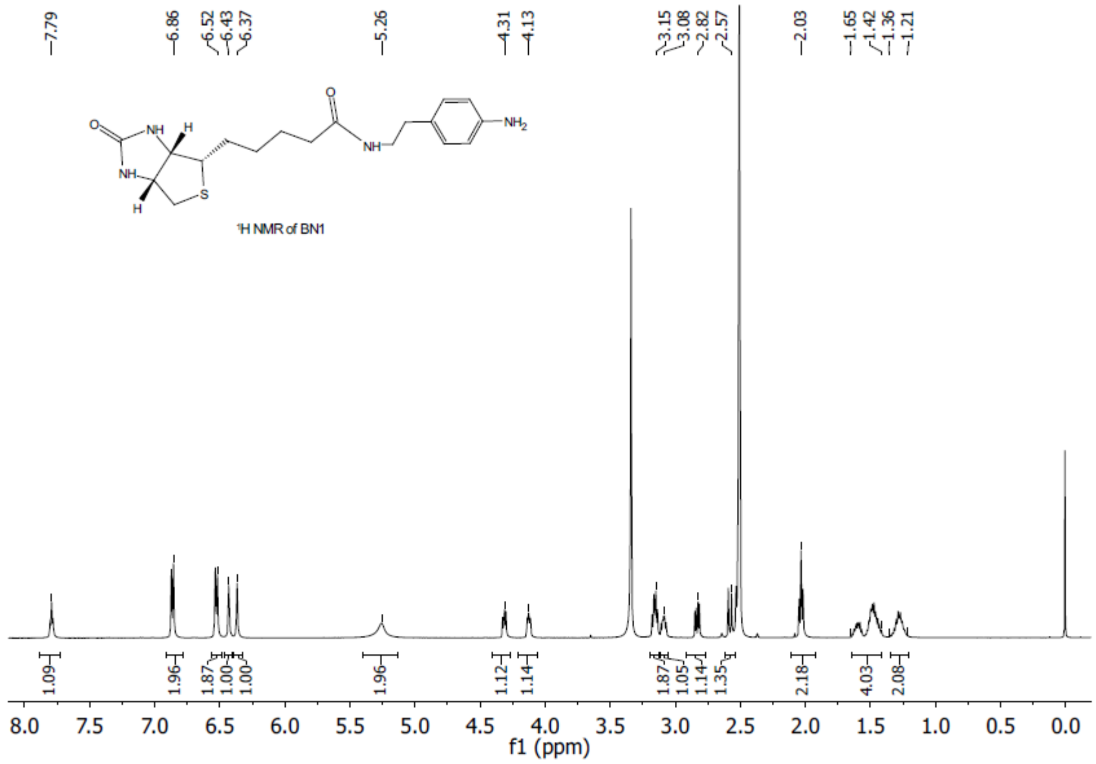

^{1}H NMR spectra of BN1

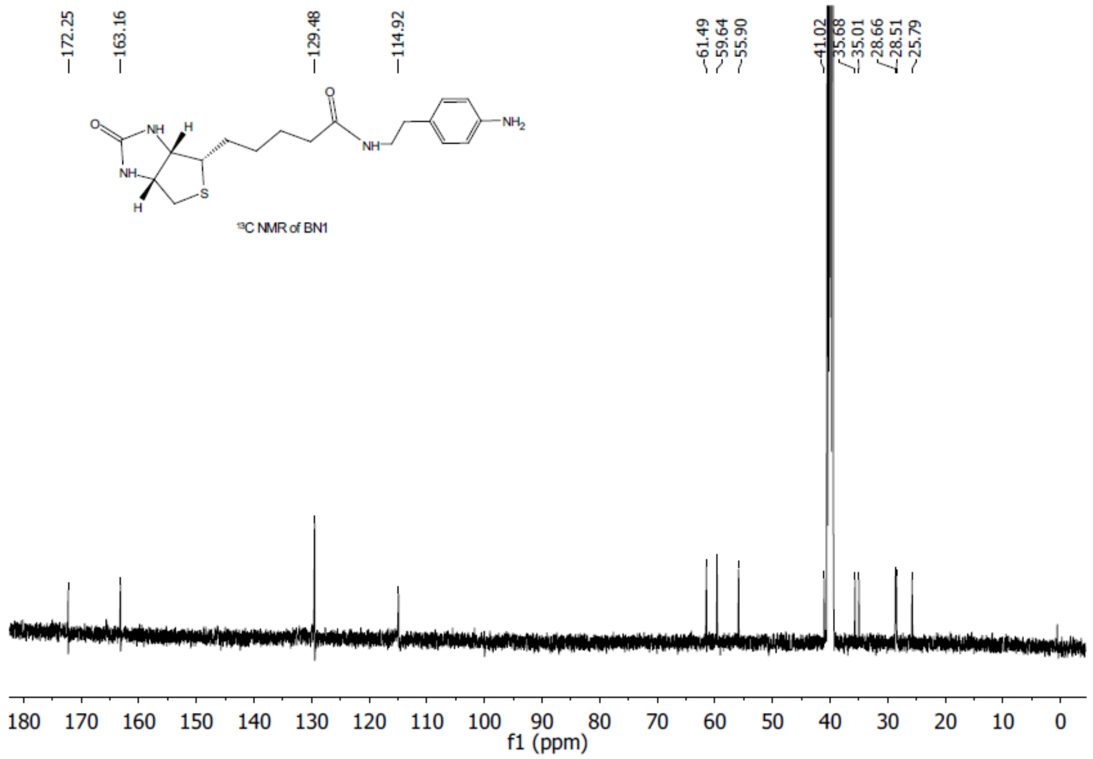

^{13}C NMR spectra of BN1

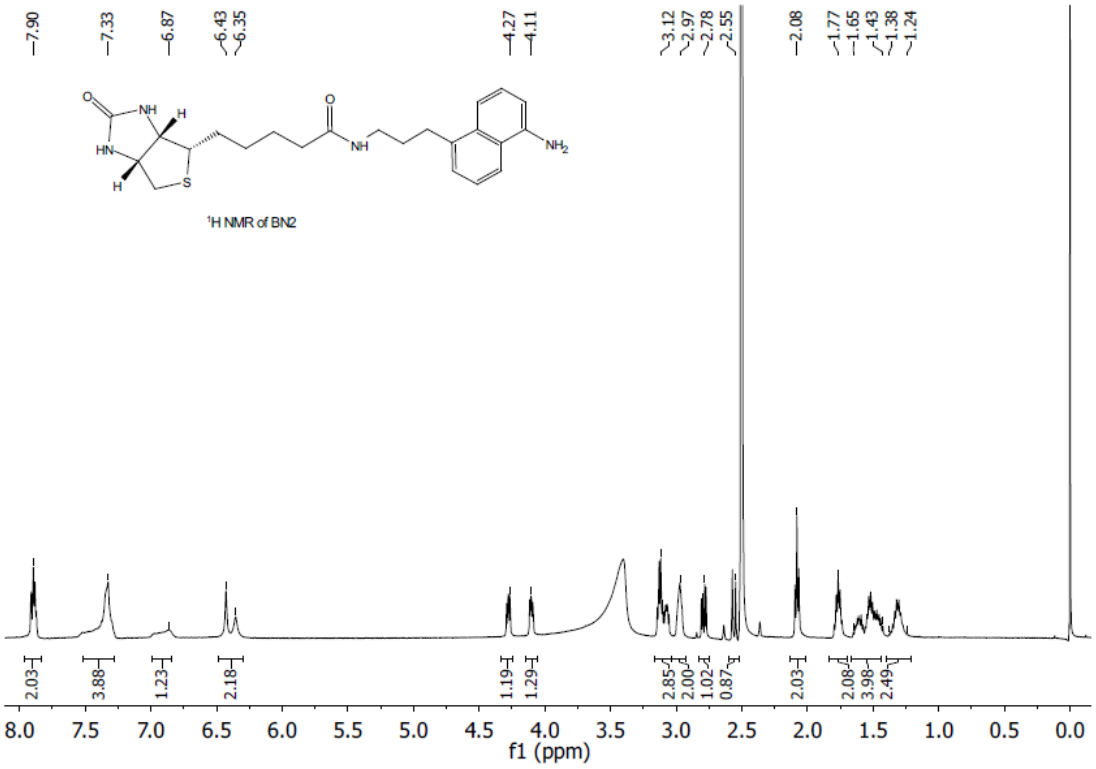

^{1}H NMR spectra of BN2

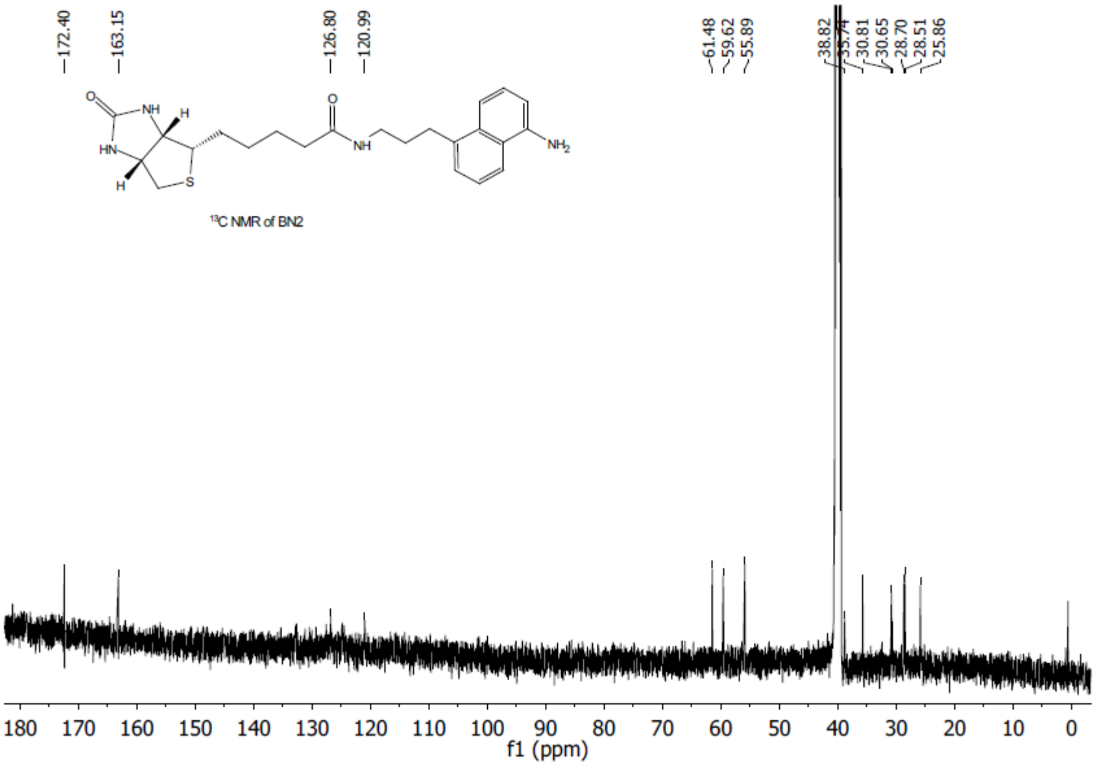

^{13}C NMR spectra of BN2

Supplementary Figures 1-13

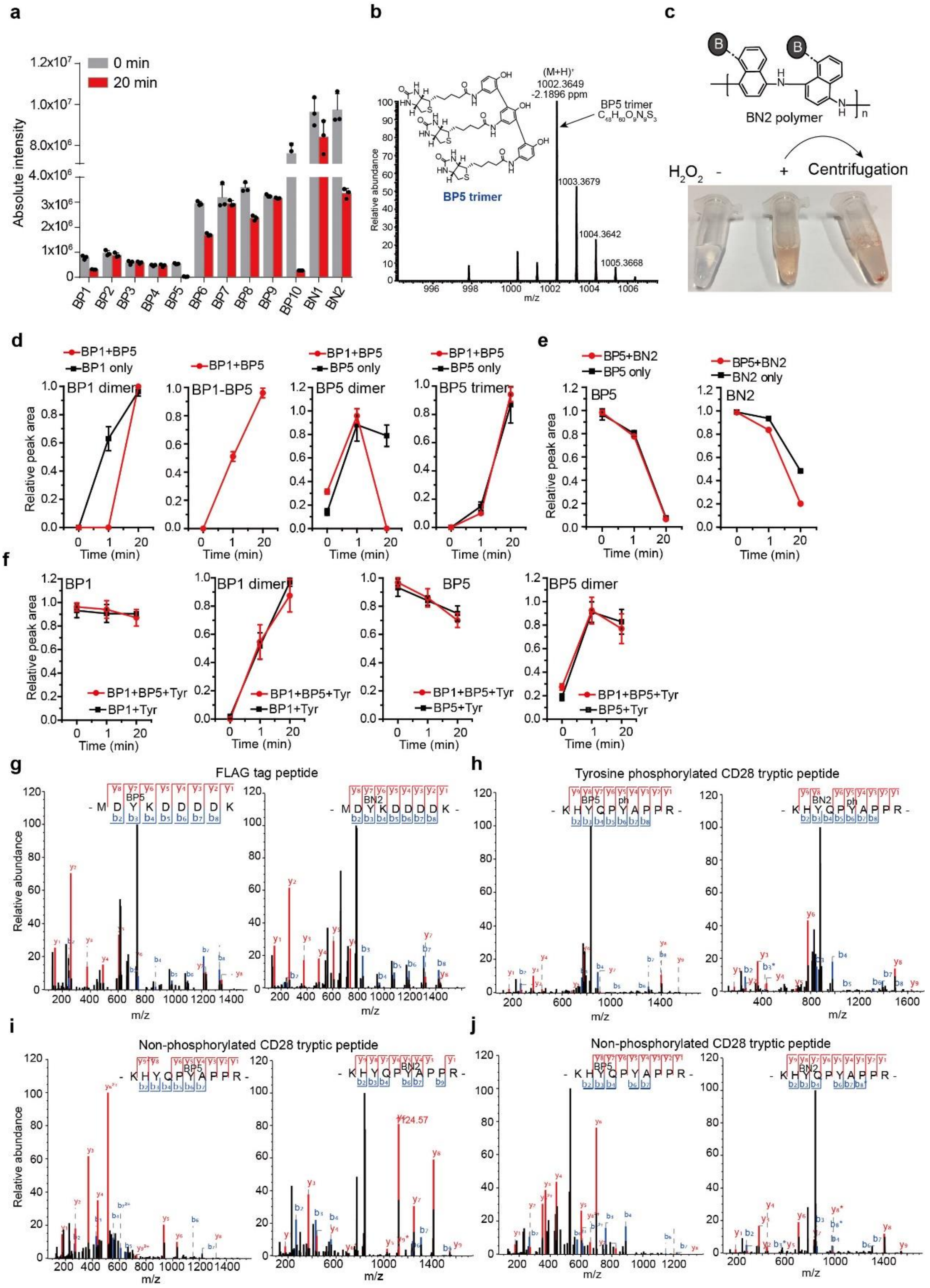

Supplementary Figure 1 (related to **Fig. 1**). **a.** Absolute intensity of BP derivatives before and after the 20 min in vitro reactions with 10 nM HRP and 1 mM H_2O_2. Data are presented as mean values +/- s.d. (error bars) (n=3 independent biological

experiments). **b.** High resolution mass spectrum of the BP5 trimer structure after the BP5 reaction same as in **a**. **c.** Red-colored BN2 polymer produced upon the reaction same as in **a**. The solutions in the first two tubes indicated the BN2 reaction with or without H_2O_2. The third tube indicated the red-colored precipitants after centrifugation at 12,000 ×g for 5 min. **d.** Comparison of the reactivity of BP1 and BP5 (related to **Fig. 1e**). Data are presented as mean values +/- s.d. (error bars) (n=3 independent biological experiments). **e.** Comparison of the reactivity of BP5 and BN2 with the same experimental design as **d**. Data are presented as mean values +/- s.d. (error bars) (n=3 independent biological experiments). **f.** Comparison of the reactivity of BP1 and BP5 with tyrosine (related to **Fig. 1f**). Data are presented as mean values +/- s.d. (error bars) (n=3 independent biological experiments). **g-j.** MS/MS spectra of indicated BP5- and BN2-modified peptides after reactions with 10 nM HRP and 1 mM H_2O_2 in vitro. FLAG tag peptide (**g**), chemically synthesized tyrosine phosphorylated CD28 peptide (phosphorylated at the second tyrosine (**h)** and chemically synthesized non-phosphorylated CD28 tryptic peptide (**i-j**) were chemically synthesized and added into the reaction same as **d** with concentration of 500 µM. All the reactions were performed in triplicate. Source data are provided as a Source Data file.

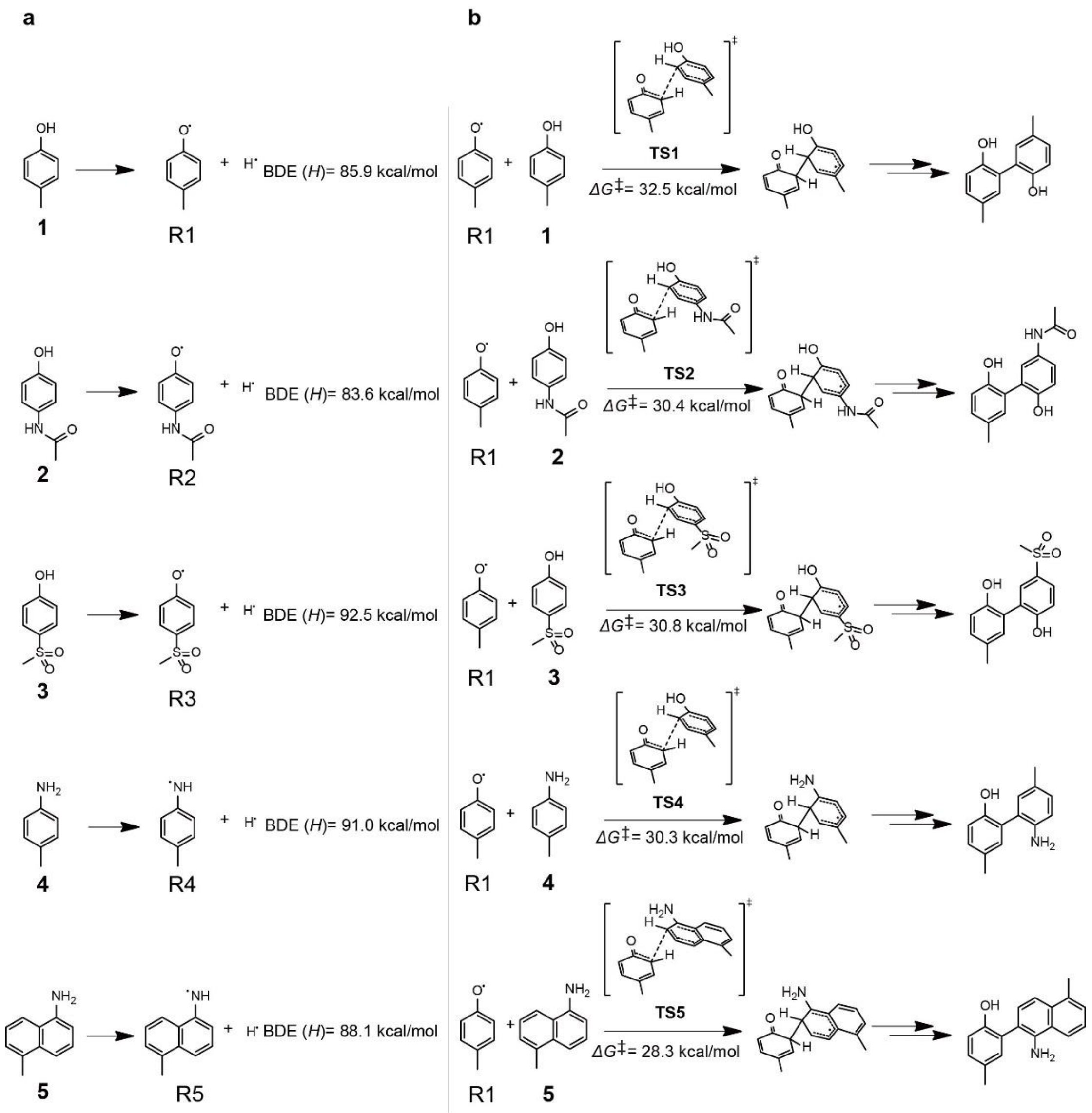

Supplementary Figure 2 (related to **Fig. 1**)**.** In principle, the proximity labeling could be attributed by two reaction steps: (1) radical generation as determined by BDE and (2) radical reaction between probe and tyrosine residue as determined by $\Delta G^{\ddagger}$. The DFT-computed BDE of model compounds 1−5 **(a)** and their corresponding reaction barriers with R1 radical as indicated by $\Delta G^{\ddagger}$ **(b)**.

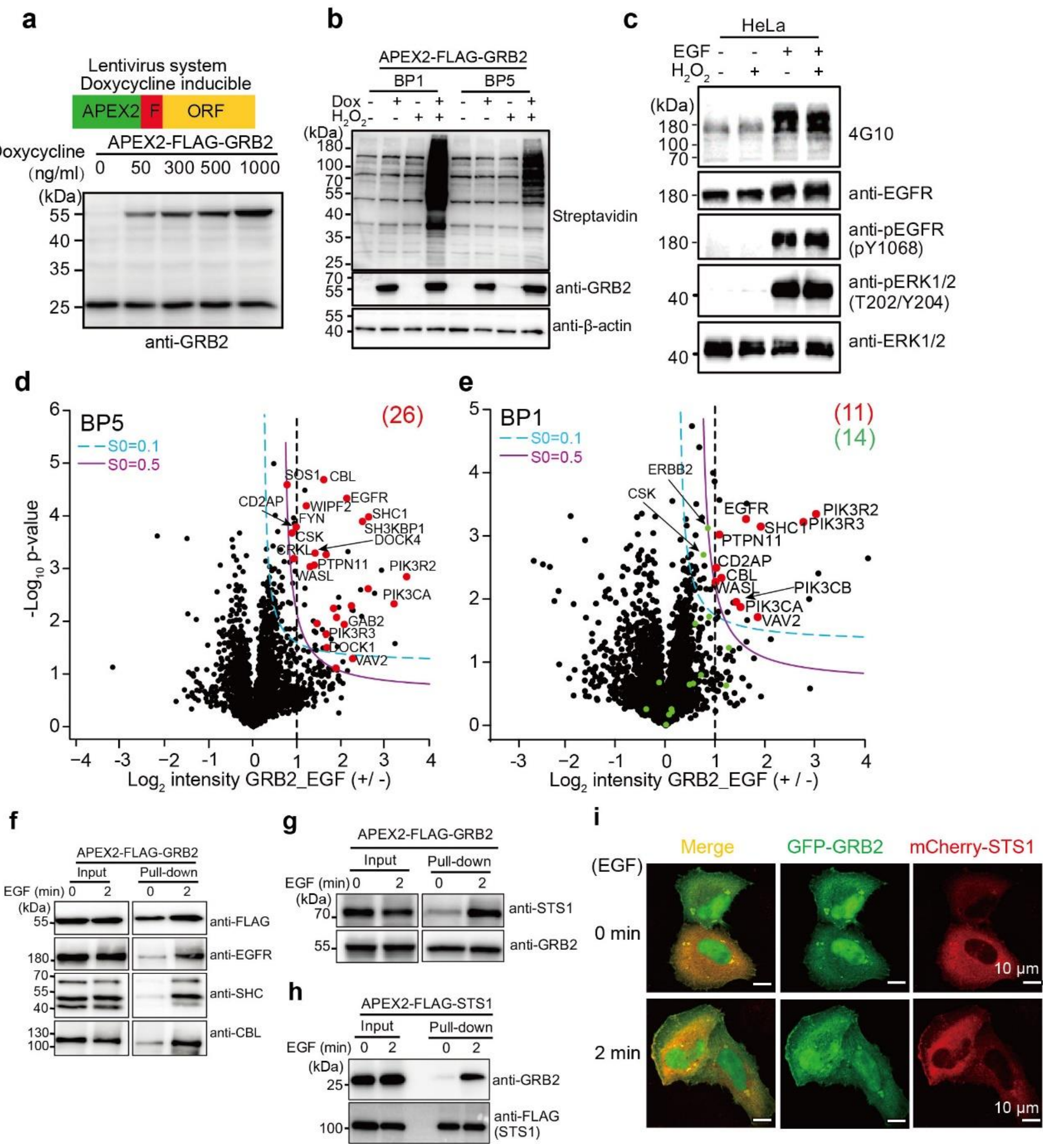

Supplementary Figure 3 (related to **Fig. 2**). **a.** Scheme of the doxycycline-inducible lentivirus plasmid. The expression level of APEX2-FLAG-GRB2 protein in the stable cell line was regulated by doxycycline concentration (n=3 independent biological experiments). Western blot by anti-GRB2 indicated the expression level of both endogenous and exogenously expressed GRB2. **b.** Both BP5 and BP1 mediated labeling in an APEX2- and H_2O_2-dependent manner (n=3 independent biological experiments). **c.** H_2O_2 did not affect global tyrosine phosphorylation level as indicated by 4G10 antibody blot and specific pTyr sites on EGFR and ERK as indicated by specific antibody blot (n=3 independent biological experiments). HeLa cells were treated with 500 µM H_2O_2 and or 100 ng/mL EGF. **d-e.** Comparison between BP5- and

BP1-based GRB2 interactome data with indicated S0 cutoffs in the volcano plots. The cutoff for log2 fold change =1 was indicated. In volcano plot for BP5, 26 reported GRB2-interacting proteins which meet the S0 cutoff of 0.5 were highlighted in red. In volcano plots for BP1, 11 reported GRB2-interacting proteins which meet the S0 cutoff of 0.5 were highlighted in red, while 14 reported GRB2-interacting proteins which is significant in BP5 plot but not in BP1 plot were labeled in green. This analysis indicated the proper S0 cutoff as 0.5 for including known GRB2-interacting proteins while excluding background noise. **f.** Validation of EGFR, CBL and SHC1 as EGF stimulation-dependent GRB2-interacting proteins. APEX2-FLAG tagged GRB2 stably expressed in HeLa cells was proximity labeled by BP5 and pulled down by streptavidin beads, and indicated proteins were blotted (n=3 independent biological experiments). **g-h.** Reciprocal pull down-western blot validation of GRB2 as EGF stimulation-dependent STS1-interacting protein (n=3 independent biological experiments). Experiments were performed same as **f** in APEX2-FLAG tagged STS1 or GRB2 stably expressed in HeLa cells. **i.** Colocalization of transiently co-transfected GFP-tagged GRB2 and mCherry-tagged STS1 upon EGF stimulation (n=3 independent biological experiments). The scale bar was 10 μm. Results were quantitatively confirmed by line profile analysis shown in **Supplementary Figure 13a,** and quantification of biological replicates were included in **Source Data file**. Quantification of all the WBs in this figure were presented in **Supplementary Figure 12 e-j**. Source data are provided as a Source Data file.

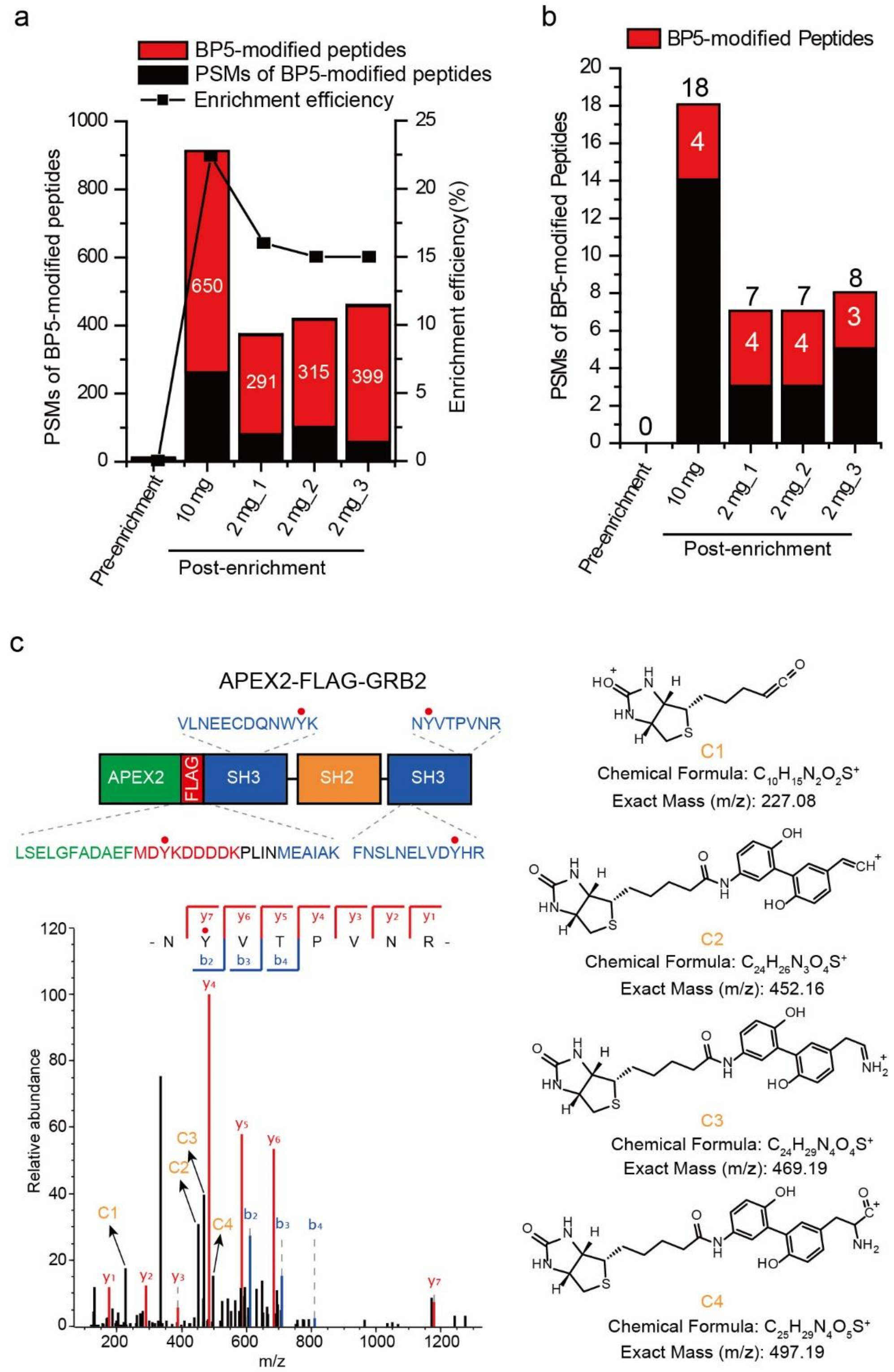

Supplementary Figure 4 (related to **Fig. 2**). **a.** Identification and enrichment efficiency of BP5-modified peptides from APEX2-FLAG-GRB2 cell line. Different amount of starting materials was adopted. The enrichment efficiency was calculated as: PSM number of BP5-modified peptides/ Total identified PSMs×100%. **b.** Identified BP5-modified peptides and PSMs of bait protein APEX2-FLAG-GRB2 were highlighted. **c.**

Identified BP5-modified peptide sequences and modification sites of APEX2-FLAG-GRB2. Representative MS/MS spectra of BP5-modified peptides for GRB2 were highlighted**.** Observed signature fragment ions of BP5 upon HCD fragmentation were shown. C1 and C3 type of fragment ions have been reported previously[8, 9], while C2 and C4 were newly defined in this study. Source data are provided as a Source Data file.

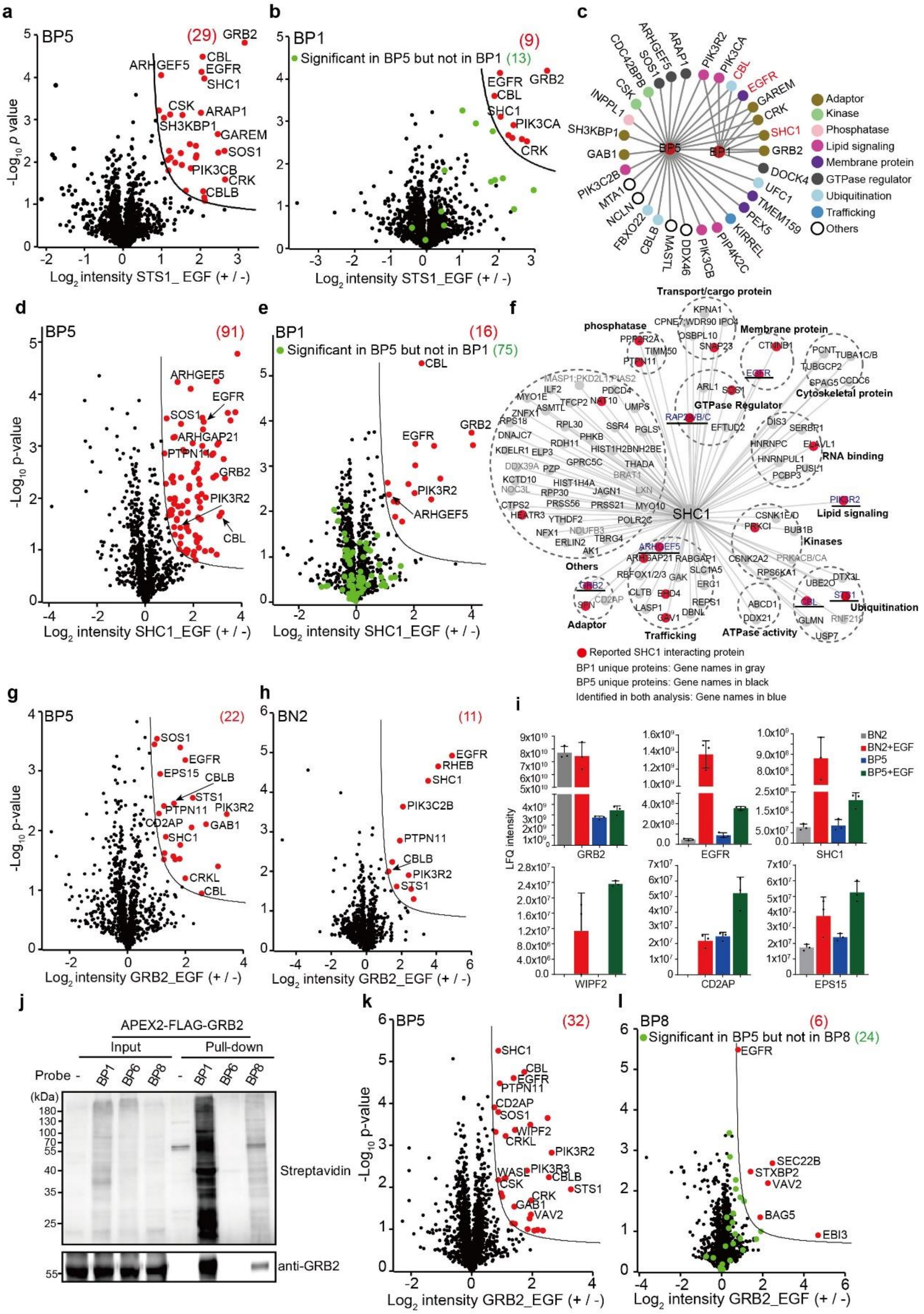

Supplementary Figure 5 (related to **Fig. 2**). **a-b.** Volcano plots of STS1-interacting proteins quantified by BP5- and BP1-based proximity proteomics (n=3 independent biological experiments). Significantly changed proteins upon 2 min EGF stimulation were highlighted in red (FDR<0.05 and S0=0.5). The relevant proteins that are

significant in BP5 experiment but not in BP1 experiment were marked in green. **c.** Interaction map and function annotation of significant STS1-associated proteins identified by BP5- and BP1-based proximity proteomics. The colored circles indicate the functional classification. Known STS1-associated proteins were labeled in red. **d-e.** Volcano plots of SHC1-interacting proteins quantified by BP5- and BP1-proximity proteomics (n=3 independent biological experiments). Significantly changed proteins upon 2 min EGF stimulation were highlighted in red (FDR<0.05 and S0=0.5). The relevant proteins that are significant in BP5 experiment but not in BP1 experiment were marked in green. **f.** Interaction map and function annotation of significant SHC1-associated proteins identified by BP5- and BP1-proximity proteomics. The identified proteins with same GO functional classification were clustered in the same circle. **g-h.** Volcano plots of GRB2-interacting proteins quantified by BP5- and BN2-based proximity proteomics (n=3 independent biological experiments). Significantly changed proteins upon 2 min EGF stimulation were highlighted in red (FDR<0.05 and S0=0.5). **i.** LFQ intensity comparison of GRB2 interactome identified by the BP5- and BN2-based proximity proteomic analysis. The bait protein GRB2 and known direct interacting proteins EGFR and SHC1 were listed in the top panel, while known indirect interacting proteins WIPF2, CD2AP and EPS15 were listed in the bottom panel. Data are presented as mean values +/- s.d. (error bars) (n=3 independent biological experiments). **j.** Streptavidin western blot analysis of in vivo labeling activity of BP1, BP6 and BP8 in HeLa cells with stably expressed APEX2-FLAG-GRB2 fusion protein (n=3 independent biological experiments). After incubating the cell with probes for 30 min, 500 µM of H_2O_2 was added for 1 min reaction. Quantification was presented in **Supplementary Figure 12k**. **k-l.** Volcano plots of GRB2-interacting proteins quantified by BP5- and BP8-based proximity proteomics (n=3 independent biological experiments). Significantly changed proteins upon 2 min EGF stimulation were highlighted in red (FDR<0.05 and S0=0.5). The relevant proteins that are significant in BP5 experiment but not in BP8 experiment were marked in green. Source data are provided as a Source Data file.

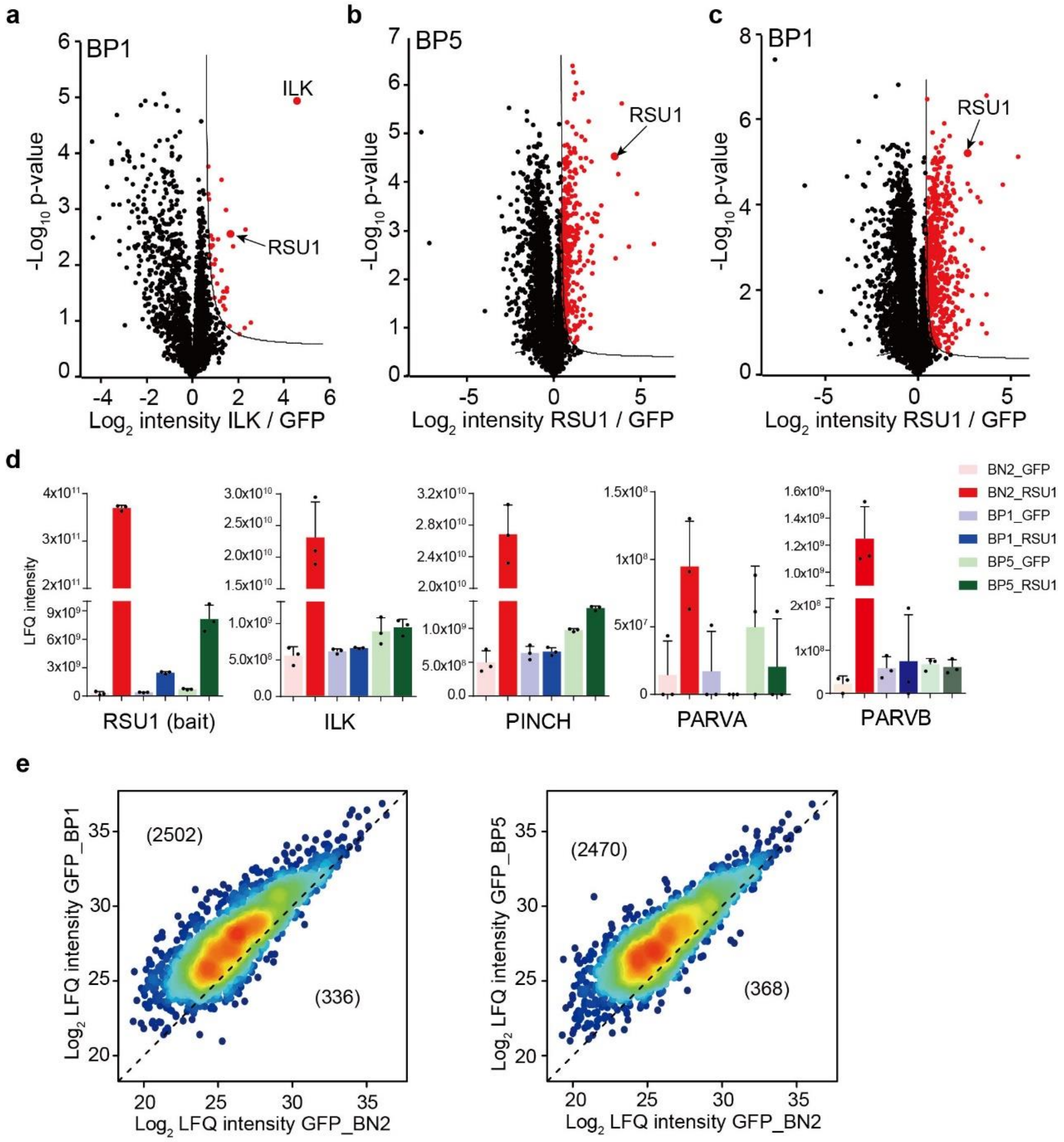

Supplementary Figure 6 (related to **Fig. 3**)**. a** (**associated with Fig. 3b-c**)**.** Volcano plot of BP1-based proximity proteomic analysis with stably expressed APEX2-FLAG-ILK in HT1080 cells as the bait protein (n=3 independent biological experiments). **b-c** (**associated with Fig. 3d**)**.** Volcano plot comparison between BP5- and BP1-based proximity proteomic analysis with stably expressed APEX2-FLAG-RSU1 in HT1080 cells as the bait protein (n=3 independent biological experiments). **d.** LFQ intensity comparison of the ILK-PINCH-PARVIN-RSU1 complex proteins identified by the BP1-, BP5- and BN2-based proximity proteomic analysis with RSU1 as bait protein. Data are presented as mean values +/- s.d. (error bars) (n=3 independent biological experiments). **e.** Comparison of the background labeling for BP1 vs. BN2 and BP5 vs.

BN2. LFQ intensity of the identified proteins in the GFP groups (in ILK/GFP experiment) were used for the analysis. Only proteins with unique peptides > 1 and at least 2 valid LFQ intensity values (after log_2) in triplicate analysis were considered for the following analysis. The numbers in the brackets represent the protein numbers located on the related side of the diagonal sections. Source data are provided as a Source Data file.

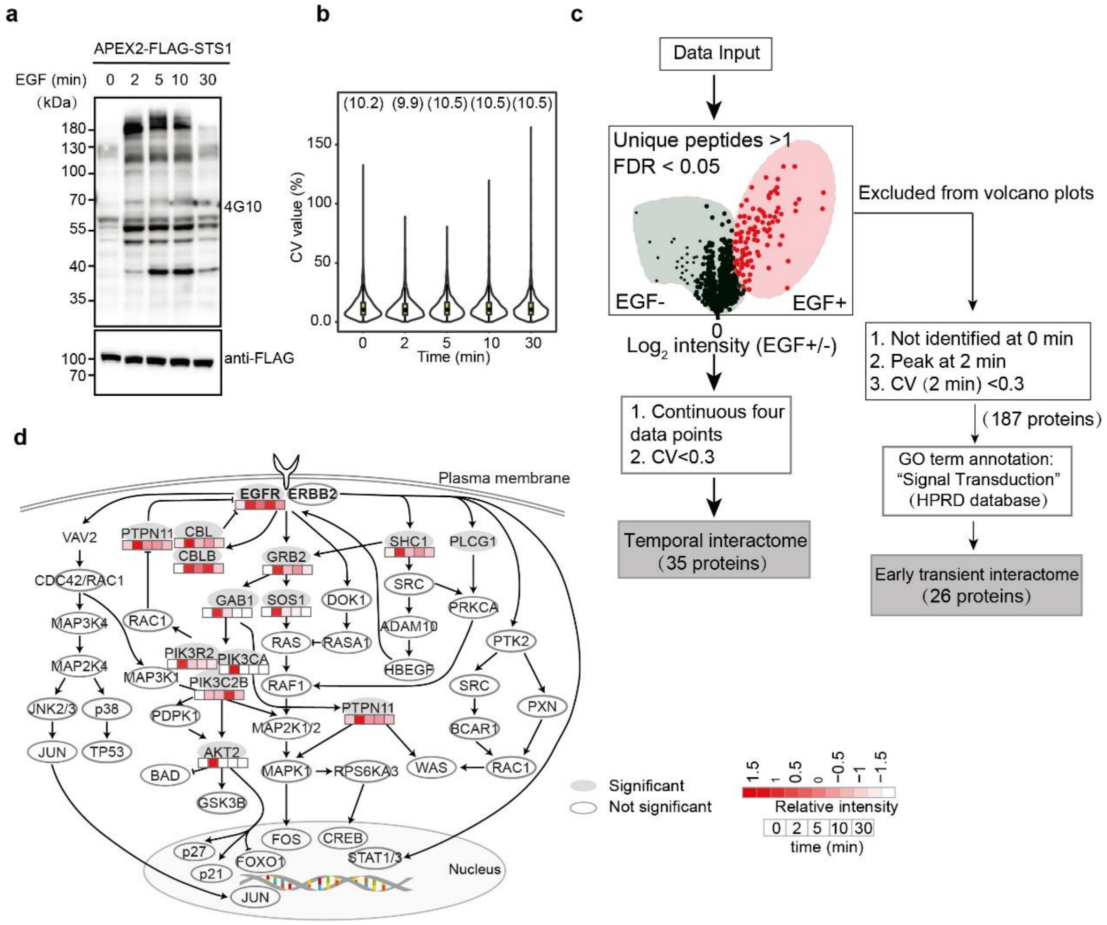

Supplementary Figure 7 (related to **Fig. 4**). **a.** Validation of EGF stimulation performance in HeLa cells with stably expressed APEX2-FLAG-STS1 by 4G10-blotted western blot (n=3 biologically independent experiments). Quantification was presented in **Supplementary Figure 12l**. **b.** CV distribution of the proteins identified in the EGF time-course experiments. The numbers in the brackets indicate the median values of the CVs in the corresponding experiments (n=3 biologically independent experiments). The violins indicate the distribution of the CV values. The medium CV values are marked with black dots in the box; the interquartile range (IQR) are presented by the box; whiskers extend to the range of 1.5 time of the IQR; CV values lying beyond the whiskers are considered as outliers. **c.** Workflow of MS data processing for both proximity proteomics related to **Fig. 4** and AP-MS related to **Fig. 5**. **d.** Pathway map of significant proteins related to EGFR/ERBB2 signaling pathway. Colors in the grids indicate relative intensity of the proteins over the five data points. The intensities are average LFQ intensity calculated from n=3 independent biological experiments. The

signaling pathway map was adopted from our recent report[10]. Source data are provided as a Source Data file.

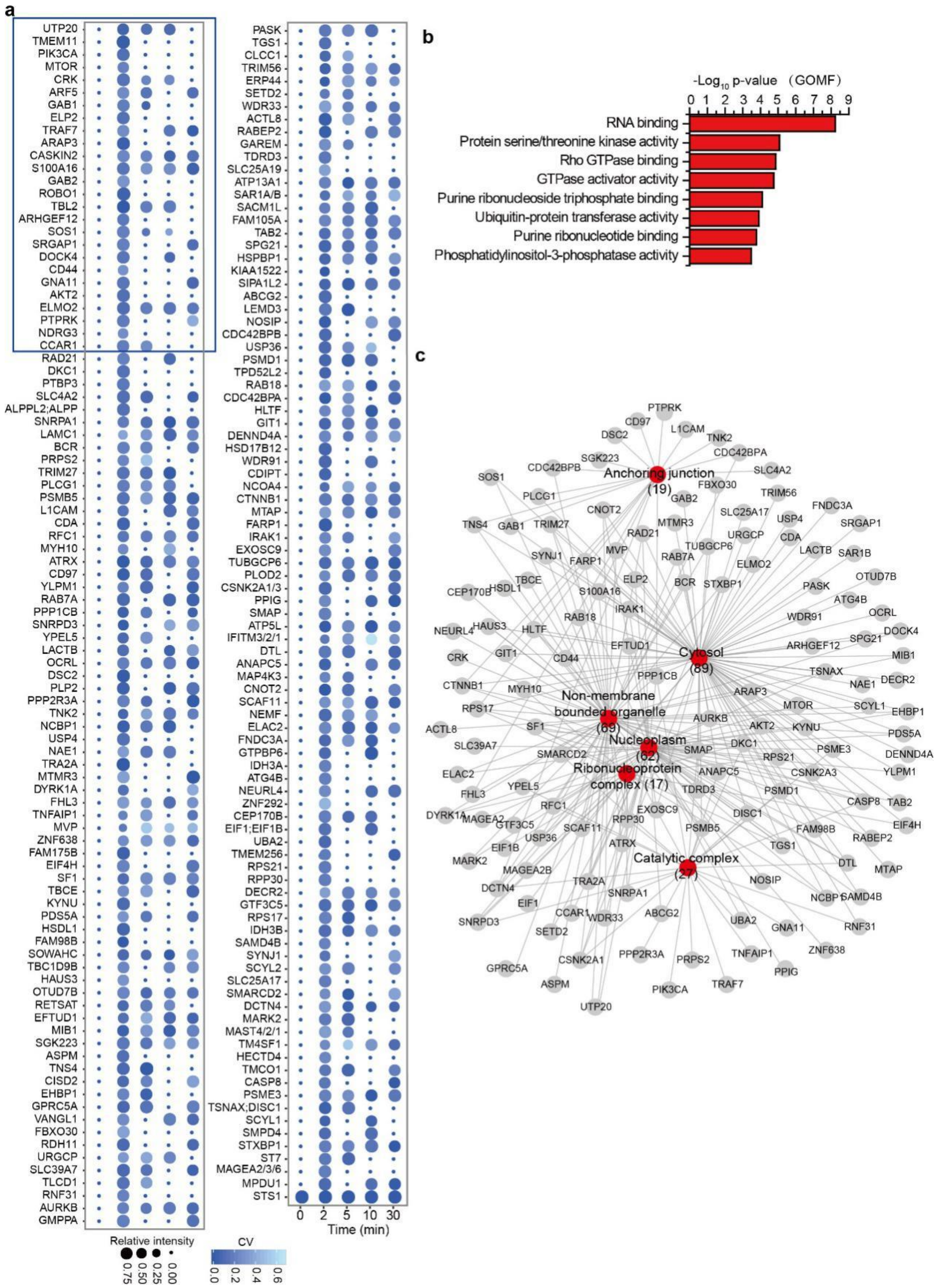

Supplementary Figure 8 (related to **Fig. 4**). **a.** Dot plots of the 187 proteins with missing values at 0 min but confident values at 2 min of EGF stimulation (CV<0.3, n=3 independent biological experiments). Relative intensity against to the highest value in the same group were presented. The CV distribution is color-coded with different intensity. The proteins with HPRD (version 9; http://www.hprd.org/) [11] annotated signal

transduction function were highlighted. **b.** GO-term molecular function (GOMF) annotation of the 187 proteins identified in **a** (FDR<0.05). **c.** Cellular component map of the 187 proteins as annotated by GO knowledgebase (FDR<0.05). The localization terms are labeled with red circles. The numbers in the brackets indicated the identified proteins associated with this term. Source data are provided as a Source Data file.

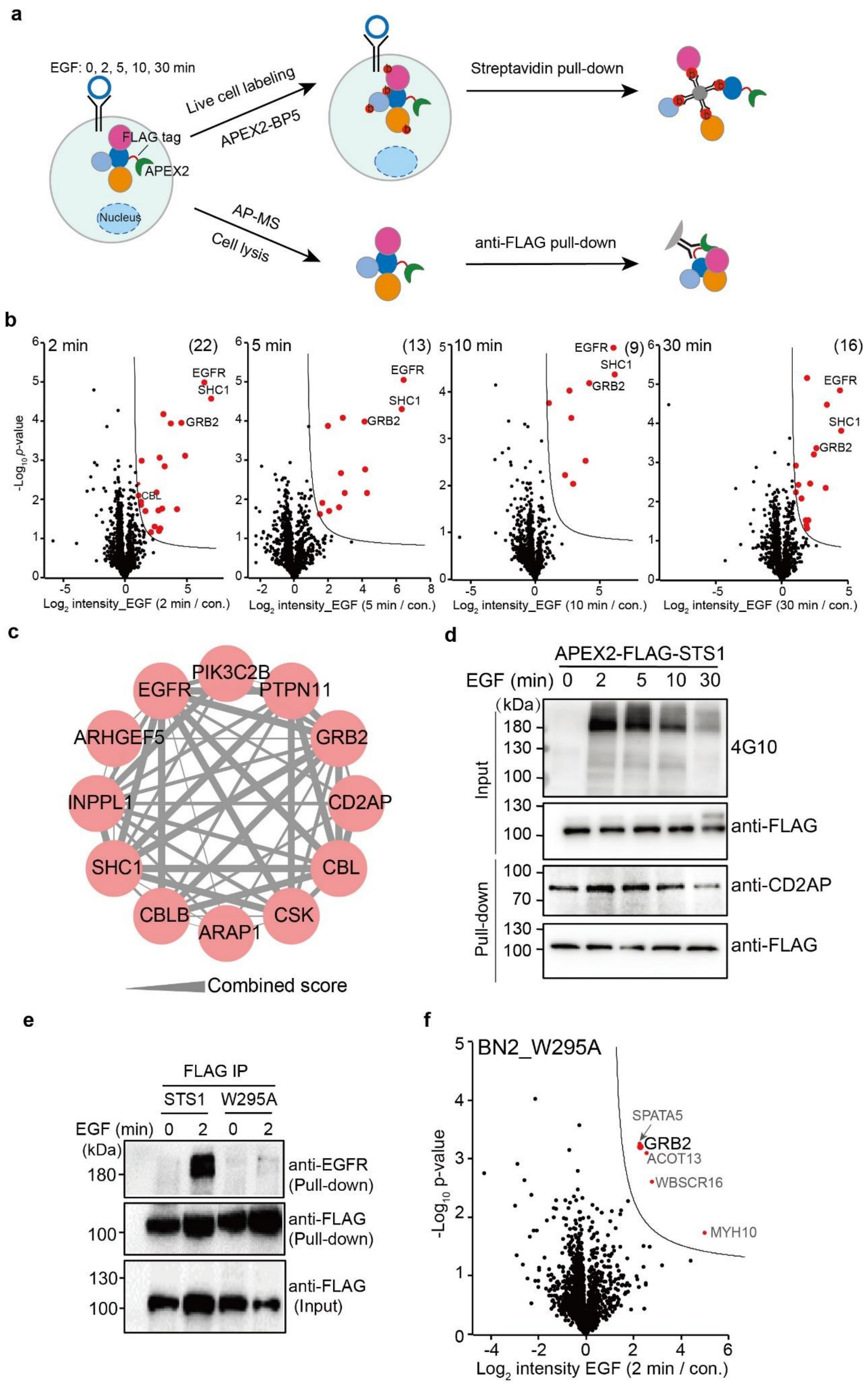

Supplementary Figure 9 (related to **Fig. 5**). **a.** Workflow comparison between proximity

proteomics and AP-MS. **b.** Volcano plots of EGF stimulation-dependent STS1 interactome characterized by AP-MS at each EGF stimulation point (n=3 independent biological experiments). Significantly changed proteins were highlighted in red (FDR<0.05 and S0=0.5). **c.** The interaction relationship of the 12 overlapped proteins as characterized by the STRING analysis (interaction score>0.25). **d.** Validation of STS1 interaction with CD2AP by pull-down of APEX2-FLAG-STS1 and western blots (n=3 independent biological experiments). Quantification was presented in **Supplementary Figure 12m**. **e.** FLAG tag-based pull-down and western blot of APEX2-FLAG-STS1 and APEX2-FLAG-STS1 with W295A mutation in SH3 domain (n=3 independent biological experiments). Quantification was presented in **Supplementary Figure 12n**. **f.** Volcano plot of BN2-based proximity proteomic analysis of W295A mutated-STS1 interactome upon 2 min EGF stimulation in HeLa cells (n=3 independent biological experiments). Significantly changed proteins were highlighted in red (FDR<0.05 and S0=0.5). Source data are provided as a Source Data file.

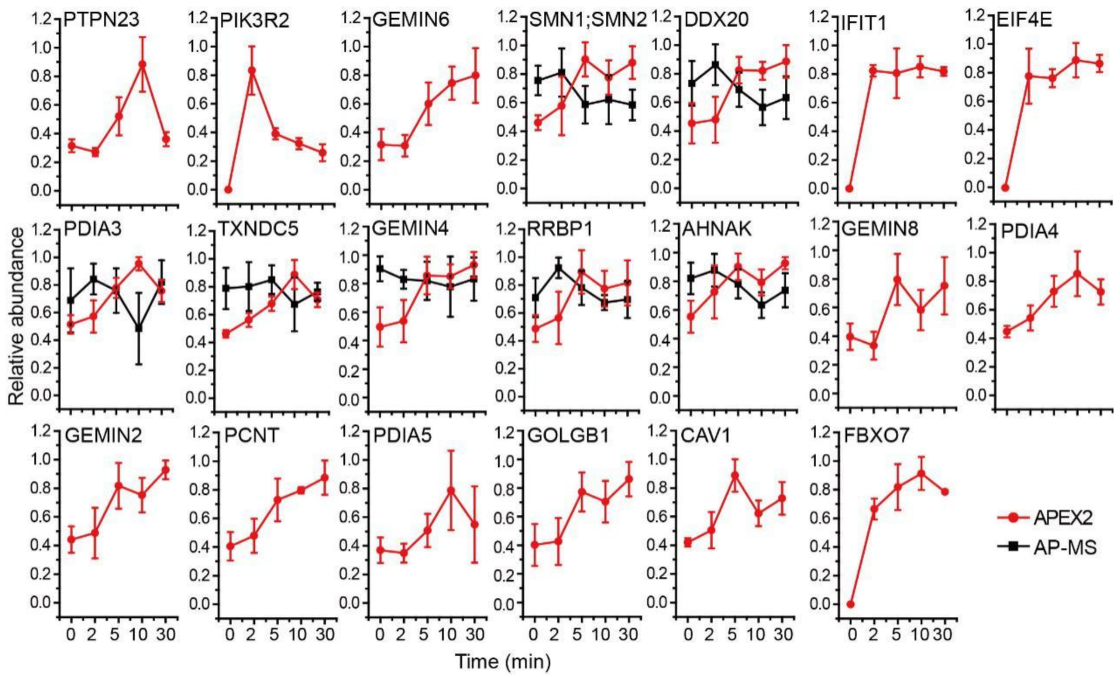

Supplementary Figure 10 (related to **Fig. 6**). Time-course curves of the STS1-interacting proteins that pass the data analysis cutoff only in APEX2-BP5 proximity proteomics. Side-by-side AP-MS data was presented when available. Data are presented as mean values +/- s.d. (n=3 independent biological experiments). The plots for RABEP1, STAM, STAM2 and HGS are separately presented in Fig. 6c. Source data are provided as a Source Data file.

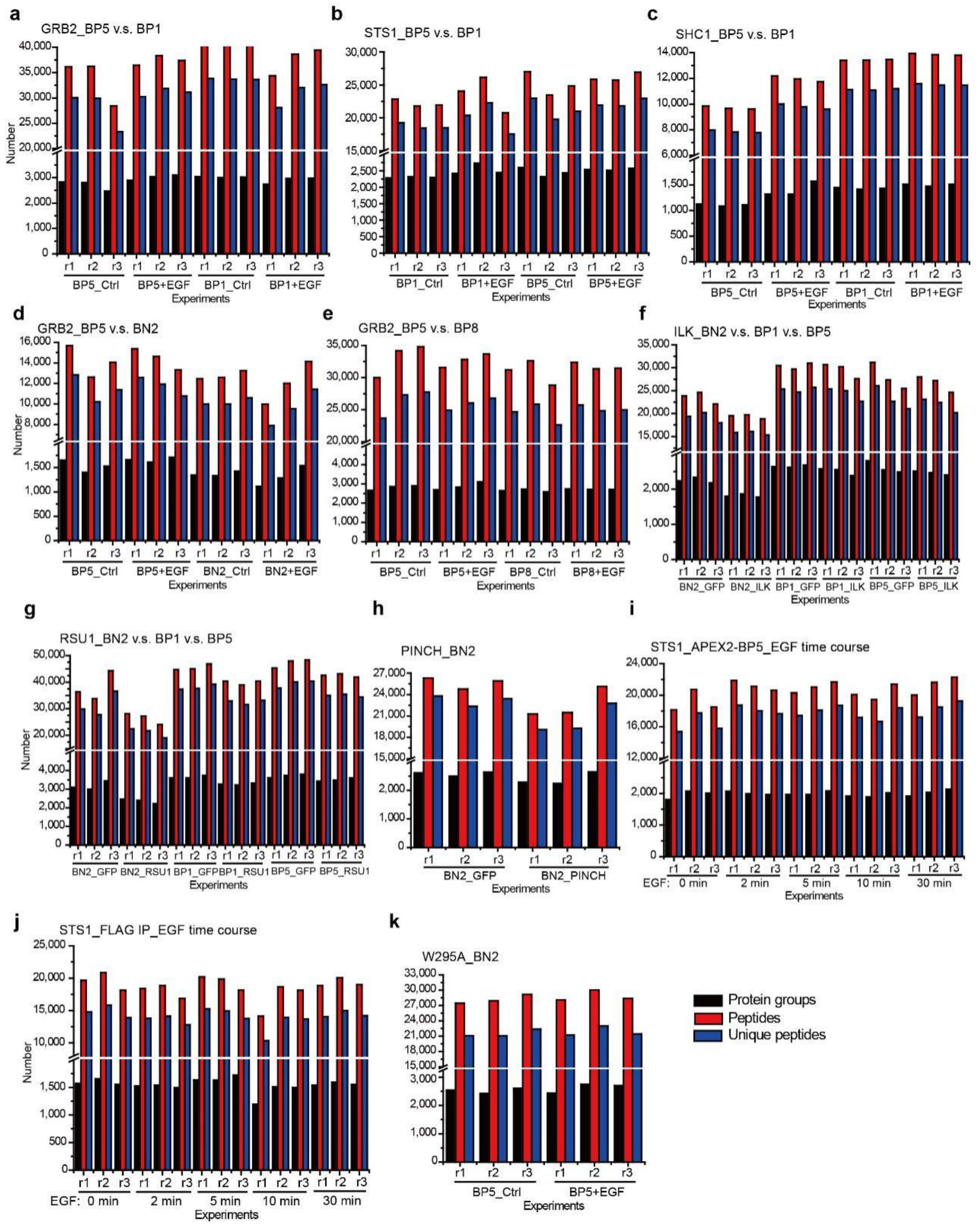

Supplementary Figure 11. Protein groups, peptides and unique peptide identification corresponding to proteomic experiments in this study. **a.** Related to **Fig. 2**. **b.** Related to **Supplementary Fig. 5a-b**. **c.** Related to **Supplementary Fig. 5d-e**. **d.** Related to **Supplementary Fig. 5g-h**. **e.** Related to **Supplementary Fig. 5k-l**. **f.** Related to **Fig. 3b-c** and **Supplementary Fig. 6a**. **g.** Related to **Fig. 3d** and **Supplementary Fig. 6b-c**. **h.** Related to **Fig. 3e**. **i.** Related to **Fig. 4**. **j.** Related to **Supplementary Fig. 9b**. **k.** Related to **Supplementary Fig. 9f**. Source data are provided as a Source Data file.

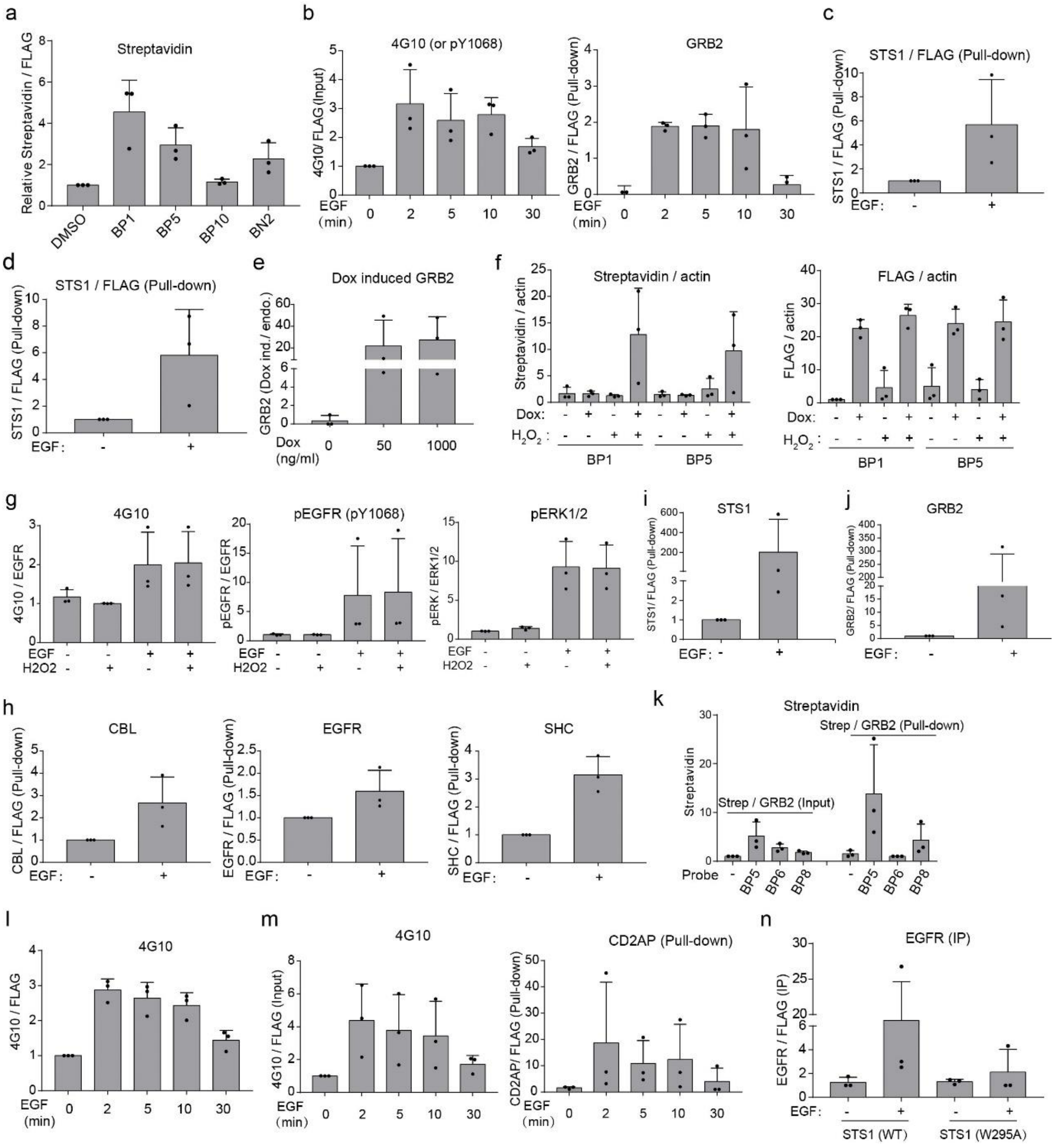

Supplementary Figure 12. Quantification for the WB figures in this study. Data are presented as mean values +/- s.d. (error bars) (n=3 independent biological experiments). **a.** Related to **Figure 1d**. **b.** Related to **Figure 4f**. **c.** Related to **Figure 4h**. **d.** Related to **Figure 5d**. **e.** Related to **Supplementary Figure 3a**. **f.** Related to **Supplementary Figure 3b**. **g.** Related to **Supplementary Figure 3c**. **h.** Related to **Supplementary Figure 3f**. **i.** Related to **Supplementary Figure 3g**. **j.** Related to **Supplementary Figure 3h**. **k.** Related to **Supplementary Figure 5j**. **l.** Related to **Supplementary Figure 7a**. **m.** Related to **Supplementary Figure 9d**. **n.** Related to **Supplementary Figure 9e**. Source data are provided as a Source Data file.

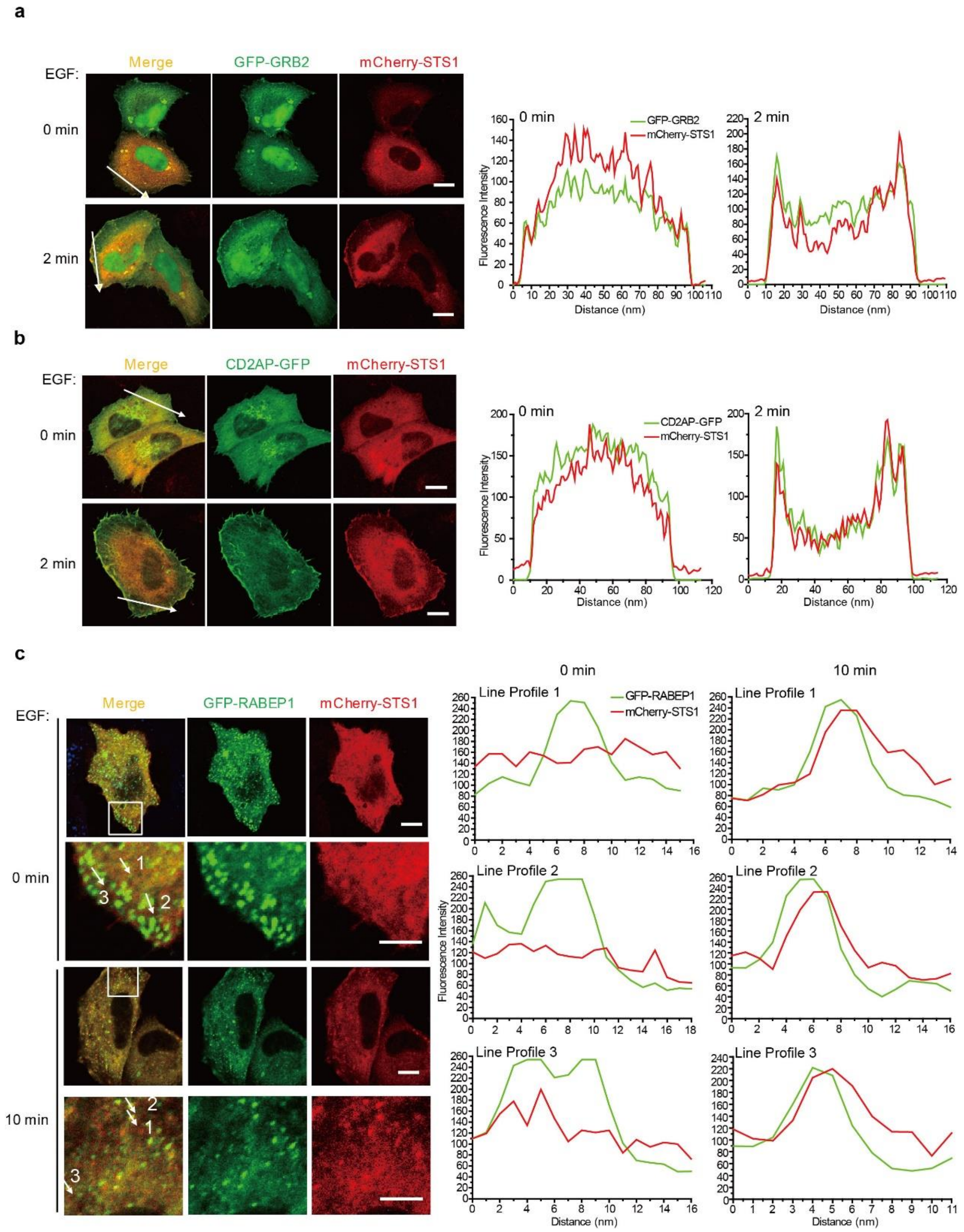

Supplementary Figure 13. Quantification for the immunofluorescence images in this study. The "Plot Profiles" were used to display the intensities of fluorescence pixel along the line. The selected image region was marked with line and arrow. Arrows indicate the direction of the line. The X-axis represents the distance along the line and the Y-axis represents the fluorescence pixel intensities. **a.** Related to **Supplementary Figure 3i**. **b.** Related to **Figure 5e**. **c.** Related to **Figure 6d**. The selected regions are labeled with "1", "2" and "3" in the zoom in sections.

The scale bars in **a**, **b** and **c** are 10 µm, and 50 µm for zoom in figures in **c**. Source data are provided as a Source Data file.

Supplementary References

1. Kwak C, Shin S, Park JS, Jung M, Nhung TTM, Kang MG, *et al.* Contact-ID, a tool for profiling organelle contact sites, reveals regulatory proteins of mitochondrial-associated membrane formation. *Proc. Natl. Acad. Sci. U. S. A.* 2020, **117**(22)**:** 12109-12120.

2. Frisch MJT, G. W.; Schlegel, H. B.; Scuseria, G. E.; Robb, M. A.; Cheeseman, J. R.; Scalmani, G.; Barone, V.; Petersson, G. A.; Nakatsuji, H.; Li, X.; Caricato, M.; Marenich, A. V.; Bloino, J.; Janesko, B. G.; Gomperts, R.; Mennucci, B.; Hratchian, H. P.; Ortiz, J. V.; Izmaylov, A. F.; Sonnenberg, J. L.; Williams-Young, D.; Ding, F.; Lipparini, F.; Egidi, F.; Goings, J.; Peng, B.; Petrone, A.; Henderson, T.; Ranasinghe, D.; Zakrzewski, V. G.; Gao, J.; Rega, N.; Zheng, G.; Liang, W.; Hada, M.; Ehara, M.; Toyota, K.; Fukuda, R.; Hasegawa, J.; Ishida, M.; Nakajima, T.; Honda, Y.; Kitao, O.; Nakai, H.; Vreven, T.; Throssell, K.; Montgomery, J. A., Jr.; Peralta, J. E.; Ogliaro, F.; Bearpark, M. J.; Heyd, J. J.; Brothers, E. N.; Kudin, K. N.; Staroverov, V. N.; Keith, T. A.; Kobayashi, R.; Normand, J.; Raghavachari, K.; Rendell, A. P.; Burant, J. C.; Iyengar, S. S.; Tomasi, J.; Cossi, M.; Millam, J. M.; Klene, M.; Adamo, C.; Cammi, R.; Ochterski, J. W.; Martin, R. L.; Morokuma, K.; Farkas, O.; Foresman, J. B.; Fox, D. J. Gaussian 16, Revision A.03. *Gaussian, Inc., Wallingford CT* 2016.

3. Becke AD. Density-Functional Thermochemistry. III. The Role of Exact Exchange. *J. Chem. Phys.* 1993, **98**(7)**:** 5648-5652.

4. Lee CY, Weitao; Parr, Robert G. Development of the Colle-Salvetti correlation-energy formula into a functional of the electron density. *Phys. Rev. B* 1988, **37**(2)**:** 785-789.

5. Zhao YT, D. G. The M06 suite of density functionals for main group thermochemistry, thermochemical kinetics, noncovalent interactions, excited states, and transition elements: two new functionals and systematic testing of four M06-class functionals and 12 other functionals. *Theor. Chem. Account* 2008, **120:** 215-241.

6. Rhee H-W, Zou P, Udeshi ND, Martell JD, Mootha VK, Carr SA*, et al.* Proteomic Mapping of Mitochondria in Living Cells via Spatially Restricted Enzymatic Tagging. *Science* 2013, **339**(6125)**:** 1328-1331.

7. Zhou Y, Wang G, Wang P, Li Z, Yue T, Wang J*, et al.* Expanding APEX2 Substrates for Spatial-specific Labeling of Nucleic Acids and Proteins in Living Cells. *Angew. Chem. Int. Ed. Engl.* 2019, **58**(34)**:** 11763-11767.

8. Renuse S, Madugundu AK, Jung JH, Byeon SK, Goldschmidt HL, Tahir R*, et al.* Signature Fragment Ions of Biotinylated Peptides. *J Am Soc Mass Spectrom* 2020, **31**(2)**:** 394-404.

9. Udeshi ND, Pedram K, Svinkina T, Fereshetian S, Myers SA, Aygun O*, et al.* Antibodies to biotin enable large-scale detection of biotinylation sites on proteins. *Nat Methods* 2017, **14**(12)**:** 1167-1170.

10. Chu B, He A, Tian Y, He W, Chen P, Hu J*, et al.* Photoaffinity-engineered protein scaffold for systematically exploring native phosphotyrosine signaling complexes in tumor samples. *Proc. Natl. Acad. Sci. U. S. A.* 2018, **115**(38)**:** E8863-E8872.

11. Keshava Prasad TS, Goel R, Kandasamy K, Keerthikumar S, Kumar S, Mathivanan S, *et al.* Human Protein Reference Database--2009 update. *Nucleic Acids Res.* 2009, **37**(Database issue)**:** D767-772.
