# Supplementary material for: Spatiotemporal profiling of cytosolic signaling complexes in living cells by selective proximity proteomics
Source: Nat Commun. 2021 Jan 4;12:71. doi: 10.1038/s41467-020-20367-x (PMC7782698; doi:10.1038/s41467-020-20367-x)
Supplement: Supplementary file 3 — Description of Additional Supplementary Files [file 41467_2020_20367_MOESM3_ESM.docx]

**Description of Supplementary Files**

**File name: Supplementary Data 1.**

Description: The products detected in the study of the probes reacting with HRP plus H2O2. Related to Figure 1.

**File name: Supplementary Data 2.**

Description: Cartesian coordinates of DFT-computed structures. Related to Supplementary Figure 2.

**File name: Supplementary Data 3.**

Description: Proteomic data for BP5 and BP1 comparison with GRB2 as the bait protein. Related to Figure 2. **Sheet 1**: Comparison of significant proteins in BP1 or BP5; **Sheet 2**: Two-sided student’s t-test of BP5 EGF+/-; **Sheet 3**: Two-sided student’s t-test of BP1 EGF+/-.

**File name: Supplementary Data 4.**

Description: Proteomic data for peptide enrichment with GRB2 as the bait protein. Related to Supplementary Figure 4. **Sheet 1:** Summary; **Sheet 2:** Identified biotinylated peptides with overlap to the significant proteins as indicated in Figure 2 b-c. **Sheet 3:** Search result of 10 mg starting material; **Sheet 4-6:** Search results for three independent biological replicates of 2 mg starting material.

**File name: Supplementary Data 5.**

Description: Proteomic data for BP5 and BN2 comparison with GRB2 as the bait protein. Related to Supplementary Figure 5g-h. **Sheet1:** Comparison of significant proteins in BP5 or BN2; **Sheet 2:** Two-sided student’s t-test result of BP5 EGF+/-; **Sheet3:** Two-sided student’s t-test result of BN2 EGF+/-.

**File name: Supplementary Data 6.**

Description: Proteomic data for BP5, BN2 and BP1 comparison with ILK as the bait protein. Related to Figure 3. **Sheet 1:** Comparison of significant proteins in BN2, BP1, or BP5; **Sheet 2-4:** Two-sided student’s t-test results of BN2, BP1 and BP5, respectively.

**File name: Supplementary Data 7.**

Description: Proteomic data for BP5, BN2 and BP1 comparison with RSU1 as the bait protein. Related to Figure 3. Comparison of significant proteins in BN2, BP1, or BP5; **Sheet 2-4:** Two-sided student’s t-test result of BN2, BP1 and BP5, respectively.

**File name: Supplementary Data 8.**

Description: Proteomic data for BN2 selective labeling with PINCH as the bait protein. Related to Figure 3. **Sheet 1:** Source data for PINCH vs. GFP volcano plot; **Sheet 2:** Result of the two-sided student’s t-test.

**File name: Supplementary Data 9.**

Description: Proteomic data for proximity labeling-based EGF-stimulation time course experiment with STS1 as the bait protein. Related to Figure 4. **Sheet 1:** Source data for EGF stimulation time course experiment; **Sheet 2-5**: Results of the two-sided student’s t-test of 2 vs. 0 min, 5 vs. 0 min, 10 vs. 0 min, and 30 vs. 0 min, respectively.

**File name: Supplementary Data 10.**

Description: Proteomic data for AP-MS-based EGF-stimulation time course experiment with STS1 as the bait protein. Related to Figure 5. **Sheet 1:** Source data for EGF stimulation time course experiment; **Sheet 2-5:** Results of the two-sided student’s t-test of 2 vs. 0 min, 5 vs. 0 min, 10 vs. 0 min, and 30 vs. 0 min, respectively.

**File name: Supplementary Data 11.**

Description: Primer list.
