# Supplementary material for: Spatiotemporal profiling of cytosolic signaling complexes in living cells by selective proximity proteomics
Source: Nat Commun. 2021 Jan 4;12:71. doi: 10.1038/s41467-020-20367-x (PMC7782698; doi:10.1038/s41467-020-20367-x)
Supplement: Supplementary file 15 — Reporting Summary [file 41467_2020_20367_MOESM15_ESM.pdf]

nature research

Corresponding author(s): Ruijun Tian

Last updated by author(s): Oct 28, 2020

Reporting Summary

Nature Research wishes to improve the reproducibility of the work that we publish. This form provides structure for consistency and transparency in reporting. For further information on Nature Research policies, see our Editorial Policies and the Editorial Policy Checklist.

Statistics

For all statistical analyses, confirm that the following items are present in the figure legend, table legend, main text, or Methods section.

n/a	Confirmed	
☐	☒	The exact sample size (n) for each experimental group/condition, given as a discrete number and unit of measurement
☐	☒	A statement on whether measurements were taken from distinct samples or whether the same sample was measured repeatedly
☐	☒	The statistical test(s) used AND whether they are one- or two-sided<br>*Only common tests should be described solely by name; describe more complex techniques in the Methods section.*
☒	☐	A description of all covariates tested
☐	☒	A description of any assumptions or corrections, such as tests of normality and adjustment for multiple comparisons
☐	☒	A full description of the statistical parameters including central tendency (e.g. means) or other basic estimates (e.g. regression coefficient) AND variation (e.g. standard deviation) or associated estimates of uncertainty (e.g. confidence intervals)
☐	☒	For null hypothesis testing, the test statistic (e.g. F, t, r) with confidence intervals, effect sizes, degrees of freedom and P value noted<br>*Give P values as exact values whenever suitable.*
☒	☐	For Bayesian analysis, information on the choice of priors and Markov chain Monte Carlo settings
☐	☒	For hierarchical and complex designs, identification of the appropriate level for tests and full reporting of outcomes
☐	☒	Estimates of effect sizes (e.g. Cohen's d, Pearson's r), indicating how they were calculated

Our web collection on statistics for biologists contains articles on many of the points above.

Software and code

Policy information about availability of computer code

Data collection	All the mass spectrometry data were collected using Thermo Scientific Xcalibur software (version 4.1.50). All fluorescence images were collected using Nikon NIS-Elements AR software (version 4.40.00). All WB images were collected using Tanon 6100C (SN 14T15RGBFLI6-1226) image system.
Data analysis	The database search and label-free quantification for mass spectrometry data were done with MaxQuant software (version 1.5.5.1) and downstream statistics analyzed by Perseus software (version 1.5.5.3). All images were analyzed using Nikon NIS-Element AR (version 4.40.00) and ImageJ software. The protein-protein relationship was analyzed with STRING (version 11.0). Molecular function and cellular compartment annotation were referred to GO knowledgebase (Released at 2020-06-01, with 44,411 GO terms and 7,975,639 annotations; FDR<0.05). Protein function annotation was referred to HPRD database (version 9) and related reports (Supplementary Figure 7a). For data analysis of peptide enrichment experiment, the raw data was searched in ProteinDiscover software (Thermo Fisher Scientific, PD1.4.1.14). No code was used for data analysis in this study.

For manuscripts utilizing custom algorithms or software that are central to the research but not yet described in published literature, software must be made available to editors and reviewers. We strongly encourage code deposition in a community repository (e.g. GitHub). See the Nature Research guidelines for submitting code & software for further information.

Data

Policy information about availability of data

All manuscripts must include a data availability statement. This statement should provide the following information, where applicable:

- Accession codes, unique identifiers, or web links for publicly available datasets
- A list of figures that have associated raw data
- A description of any restrictions on data availability

Source data are provided with this paper. All the raw MS data have been deposited to ProteomeXchange Consortium repository with the dataset identifier PXD020709 (Project Web Link: http://www.ebi.ac.uk/pride/archive/projects/PXD020709).

Field-specific reporting

Please select the one below that is the best fit for your research. If you are not sure, read the appropriate sections before making your selection.

[x] Life sciences [ ] Behavioural & social sciences [ ] Ecological, evolutionary & environmental sciences

For a reference copy of the document with all sections, see nature.com/documents/nr-reporting-summary-flat.pdf

Life sciences study design

All studies must disclose on these points even when the disclosure is negative.

Sample size	Three independent samples were selected for all experiments in this paper for the following reasons: 1. For the repeatability verification and quantitative accuracy of experimental results; 2. To meet the basic requirements of LFQ quantification method; 3. This is consistent with previous literature examples for studying dynamic protein complexes. Our experiments meet the established standards of the field.
Data exclusions	In each label-free quantification process, the proteins in the "proteingroups" table marked with "identified only by site", "potential contamination" and "reverse" are excluded at the beginning of the analysis. The exclusion criteria were pre-established by the LFQ method of Perseus software for processing the data searched in MaxQuant software.
Replication	All the mass spectrometry analysis were done with three biological replicates. For all the biological findings reported in this paper, all experiments were successfully replicated with the properly optimized condition. For western blot and imaging, the results were all repeated with at least three times (n>=3). Validation were also done by principle-wise different methods (e.g., validating MS data by western blot or co-localization imaging) and reciprocal affinity purification-western blot. Attempts at all the replication were all successful.
Randomization	Randomization was not applicable to the study, because we used cell lines with consistent generic background to perform all the studies. There is no need for sample randomization with high sample similarity.
Blinding	Experiments were not operated blinded. However, mass spectrometry data collection were blinded.

Reporting for specific materials, systems and methods

We require information from authors about some types of materials, experimental systems and methods used in many studies. Here, indicate whether each material, system or method listed is relevant to your study. If you are not sure if a list item applies to your research, read the appropriate section before selecting a response.

Materials & experimental systems

n/a	Involved in the study	
[ ]	[x]	Antibodies
[ ]	[x]	Eukaryotic cell lines
[x]	[ ]	Palaeontology and archaeology
[x]	[ ]	Animals and other organisms
[x]	[ ]	Human research participants
[x]	[ ]	Clinical data
[x]	[ ]	Dual use research of concern

Methods

n/a	Involved in the study	
[x]	[ ]	ChIP-seq
[x]	[ ]	Flow cytometry
[x]	[ ]	MRI-based neuroimaging

Antibodies

Antibodies used	The primary antibodies used in this study were 4G10 (Merck Millipore, 05-321, 1:1000), anti-pEGFR (Tyr1068) (CST, 3777S, 1:1000), anti-pERK1/2 (CST, 9101, 1:1000), anti-ERK1/2 (CST, 4695, 1:1000), streptavidin-HRP (Thermo Fisher Scientific, 21130, 1:3000), anti-FLAG (SIGMA, F1804, 1:1000), anti-EGFR (CST, 4267s, 1:1000), anti-CBL (CST, 8447S, 1:1000), anti-GRB2 (BD, 610112, 1:1000), anti-STS1 (Abcam, ab34781, 1:1000), anti-SHC (BD, 610878, 1:1000) and anti-β-actin (Beyotime, AF0003, 1:1000). The secondary antibodies include HRP-conjugated anti-rabbit (Beyotime, A0208, 1:1000) and anti-mouse (Beyotime, A0216, 1:1000). (All the

antibody information was provided in the Methods part.)

Validation	1. mouse 4G10: Merck Millipore, catalog number 05-321, Lot number 3433888. (dilution 1:1000) (https://www.merckmillipore.com/CN/zh/product/Anti-Phosphotyrosine-Antibody-clone-4G10,MM_NF-05-321?ReferrerURL=https%3A%2F%2Fcn.bing.com%2F)<br>2. Rabbit anti-pEGFR (Tyr1068): CST, catalog number 3777S, Lot number 16. (dilution 1:1000) (https://www.cellsignal.com/products/primary-antibodies/phospho-egf-receptor-tyr1068-d7a5-xp-rabbit-mab/3777?site-search-type=Products&N=4294956287&Ntt=3777s&fromPage=plp&_requestid=449261)<br>3. Rabbit anti-pERK1/2: CST, catalog number 9101, Lot number 28. (dilution 1:1000) (https://www.cellsignal.com/products/primary-antibodies/phospho-p44-42-mapk-erk1-2-thr202-tyr204-antibody/9101)<br>4. Streptavidin-HRP: Thermo Fisher Scientific, catalog number 21130, Lot number SI259565. (dilution 1:3000) (https://www.thermofisher.com/order/catalog/product/21130?SID=srch-hj-21130#/21130?SID=srch-hj-21130)<br>5. mouse anti-FLAG: SIGMA, catalog number F1804, Lot number SLCD3524. (dilution 1:1000) (https://www.sigmaaldrich.com/catalog/product/sigma/f1804?lang=zh&region=CN)<br>6. Rabbit anti-EGFR: CST, catalog number 4267s, Lot number 19. (dilution 1:1000) (https://www.cellsignal.com/products/primary-antibodies/egf-receptor-d38b1-xp-rabbit-mab/4267?site-search-type=Products&N=4294956287&Ntt=4267s&fromPage=plp&_requestid=450098)<br>7. Rabbit anti-CBL: CST, catalog number 8447S, Lot number 1. (dilution 1:1000) (https://www.cellsignal.com/products/primary-antibodies/c-cbl-d4e10-rabbit-mab/8447?site-search-type=Products&N=4294956287&Ntt=8447s&fromPage=plp&_requestid=450371)<br>8. mouse anti-GRB2: BD, catalog number 610112, Lot number 4199954. (dilution 1:1000) (https://www.bdbiosciences.com/us/applications/research/b-cell-research/intracellular-antigens/human/purified-mouse-anti--grb2-81grb2/p/610112)<br>9. Rabbit anti-STS1: Abcam, catalog number ab34781, Lot number GR278584-6. (dilution 1:1000) (https://www.abcam.com/sts1-antibody-ab34781.html)<br>10. mouse anti-SHC: BD, catalog number 610878, Lot number 8025664.(dilution 1:1000) (https://www.bdbiosciences.com/us/reagents/research/antibodies-buffers/cell-biology-reagents/cell-biology-antibodies/purified-mouse-anti-shc-30shc/p/610878)<br>11. mouse anti-β-actin: Beyotime, catalog number AF0003, Lot number AA128-1. (dilution 1:1000). (https://www.beyotime.com/product/AF0003.htm)<br>12. HRP-conjugated Goat anti-rabbit: Beyotime, catalog number A0208, Lot number 031020200515. (dilution 1:1000) (https://www.beyotime.com/product/A0208.htm)<br>13. HRP-conjugated Goat anti-mouse: Beyotime, catalog number A0216, Lot number 032320200601. (dilution 1:1000). (https://www.beyotime.com/product/A0216.htm)<br>14. rabbit anti-ERK1/2: CST, catalog number 4695, Lot number 14. (dilution 1:1000). (https://www.cellsignal.com/products/primary-antibodies/c-cbl-d4e10-rabbit-mab/8447?site-search-type=Products&N=4294956287&Ntt=8447s&fromPage=plp&_requestid=450371)

Eukaryotic cell lines

Policy information about cell lines

Cell line source(s)	HeLa, HT1080 and 293T cell lines were used in this study. All the cell lines were from ATCC.
Authentication	All the cell lines from ATCC were authenticated by STR profiling by ATCC.
Mycoplasma contamination	All the cell lines were tested for mycoplasma contamination by ATCC.
Commonly misidentified lines<br>(See ICLAC register)	No.
