# Supplementary material for: Spatiotemporal profiling of cytosolic signaling complexes in living cells by selective proximity proteomics
Source: Nat Commun. 2021 Jan 4;12:71. doi: 10.1038/s41467-020-20367-x (PMC7782698; doi:10.1038/s41467-020-20367-x)
Supplement: Supplementary file 16 — Source Data [file 41467_2020_20367_MOESM16_ESM.zip › NCOMMS-20-22505C_sd/WB and IF_Replicates and Quantification/Figure 4f/Three replicates.pptx]

## Slide 1
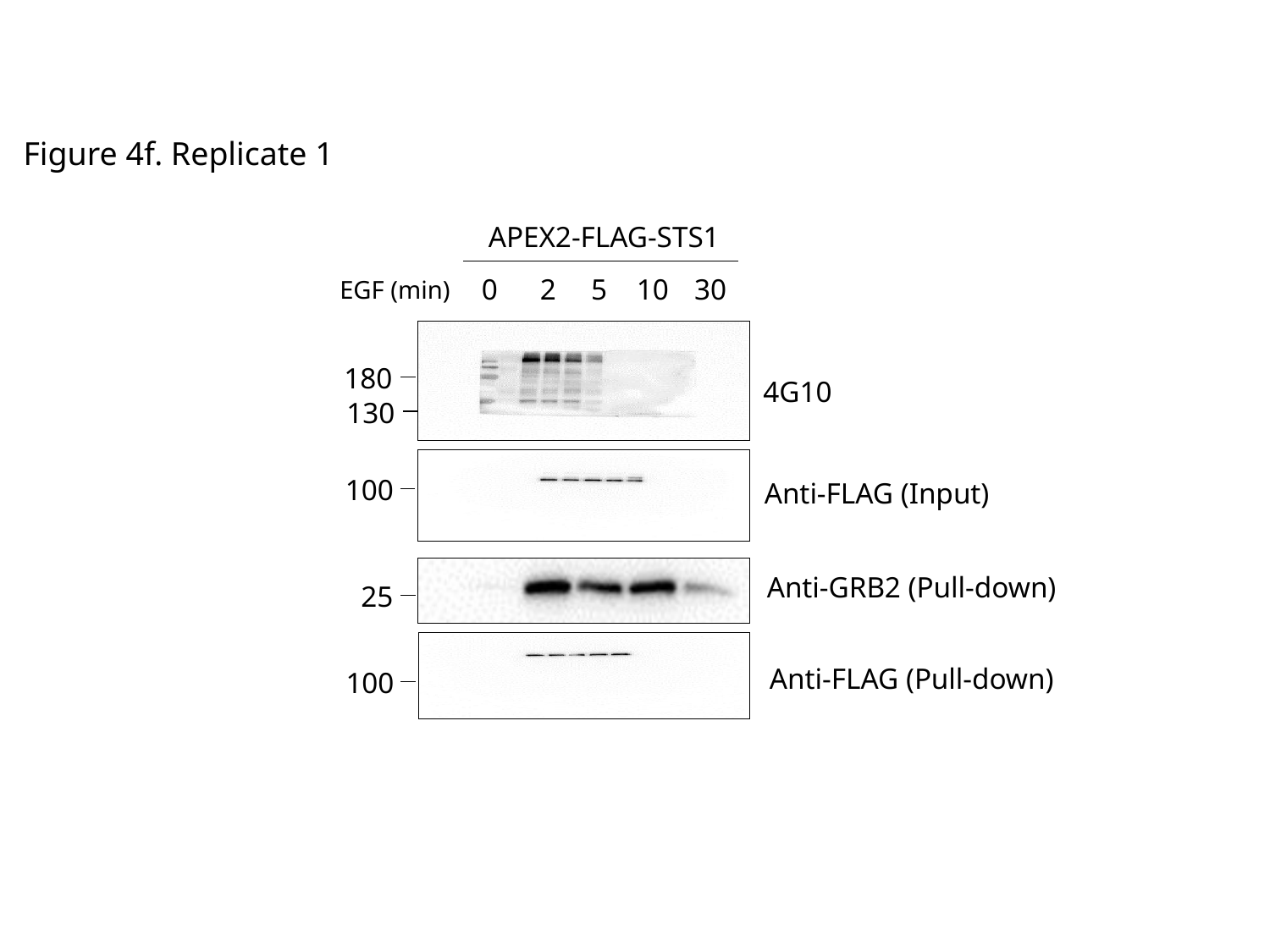

Figure 4f. Replicate 1
APEX2-FLAG-STS1
0
2
5
10
30
EGF (min)
180
4G10
130
100
Anti-FLAG (Input)
Anti-GRB2 (Pull-down)
25
Anti-FLAG (Pull-down)
100

## Slide 2
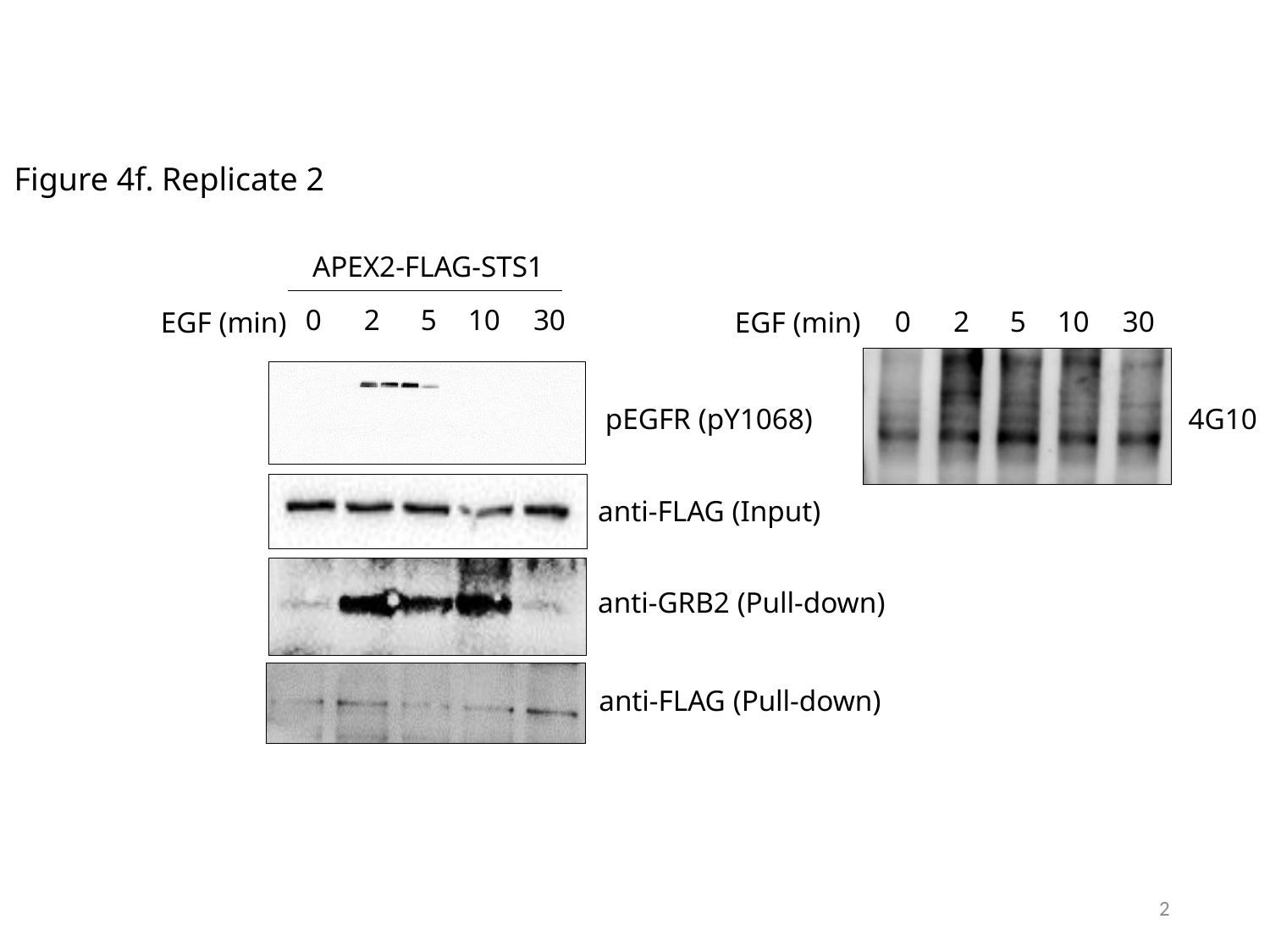

Figure 4f. Replicate 2
APEX2-FLAG-STS1
0
2
5
10
30
0
2
5
10
30
EGF (min)
EGF (min)
pEGFR (pY1068)
4G10
anti-FLAG (Input)
anti-GRB2 (Pull-down)
anti-FLAG (Pull-down)
2

## Slide 3
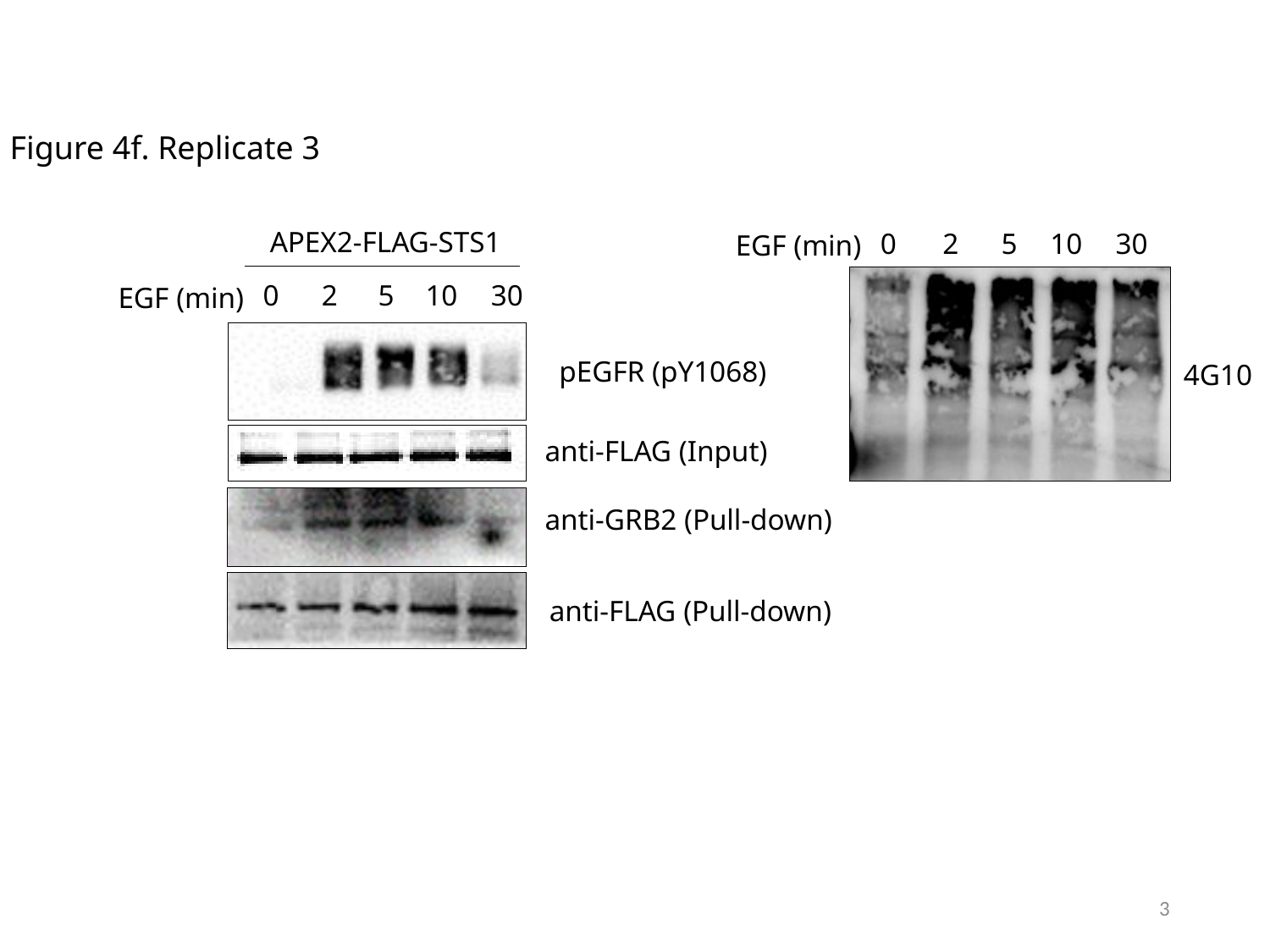

Figure 4f. Replicate 3
APEX2-FLAG-STS1
0
2
5
10
30
EGF (min)
0
2
5
10
30
EGF (min)
pEGFR (pY1068)
4G10
anti-FLAG (Input)
anti-GRB2 (Pull-down)
anti-FLAG (Pull-down)
3

## Slide 4
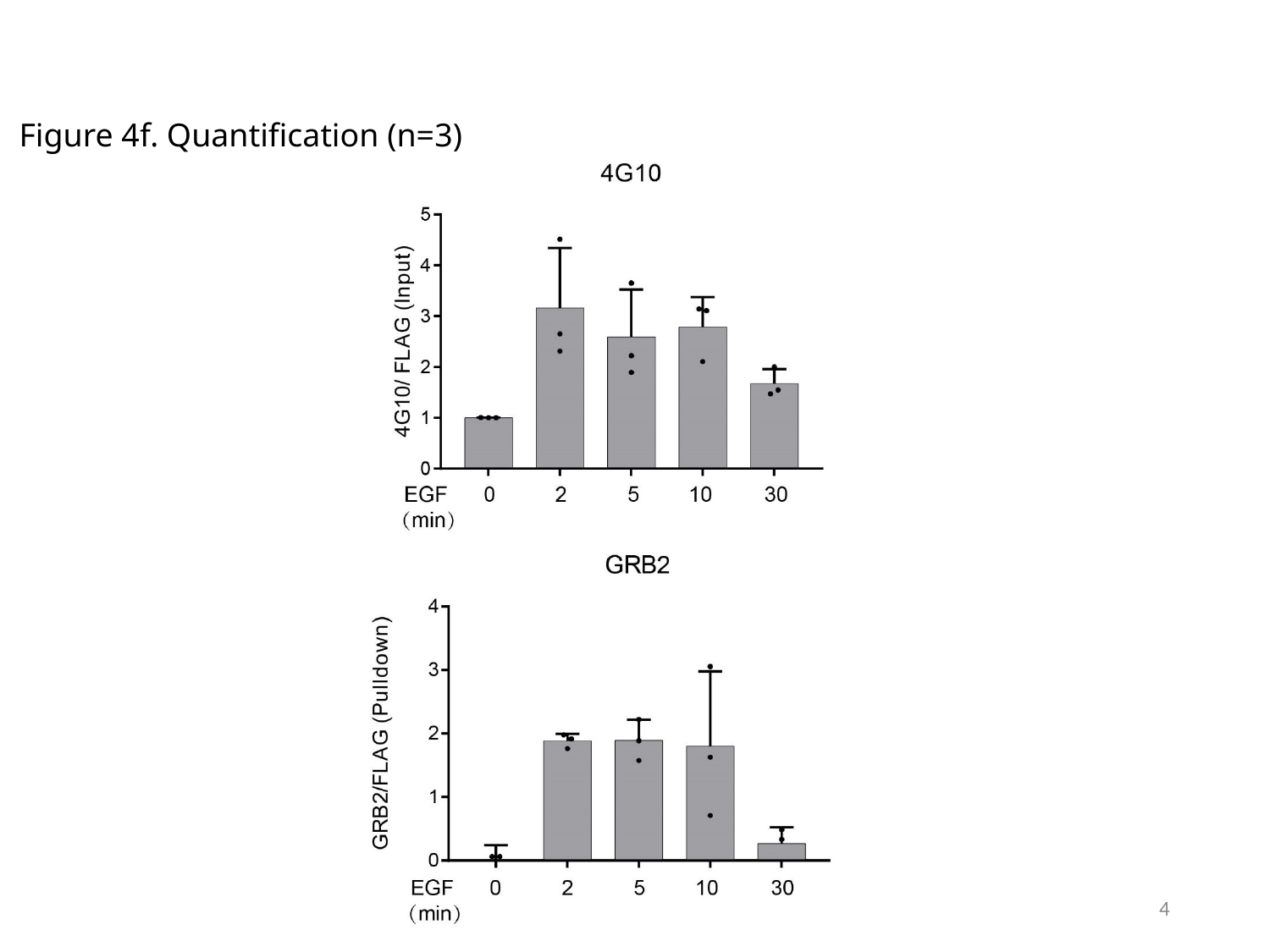

Figure 4f. Quantification (n=3)
4
