# Supplementary material for: Spatiotemporal profiling of cytosolic signaling complexes in living cells by selective proximity proteomics
Source: Nat Commun. 2021 Jan 4;12:71. doi: 10.1038/s41467-020-20367-x (PMC7782698; doi:10.1038/s41467-020-20367-x)
Supplement: Supplementary file 16 — Source Data [file 41467_2020_20367_MOESM16_ESM.zip › NCOMMS-20-22505C_sd/WB and IF_Replicates and Quantification/Figure 4h/Three replicates.pptx]

## Slide 1
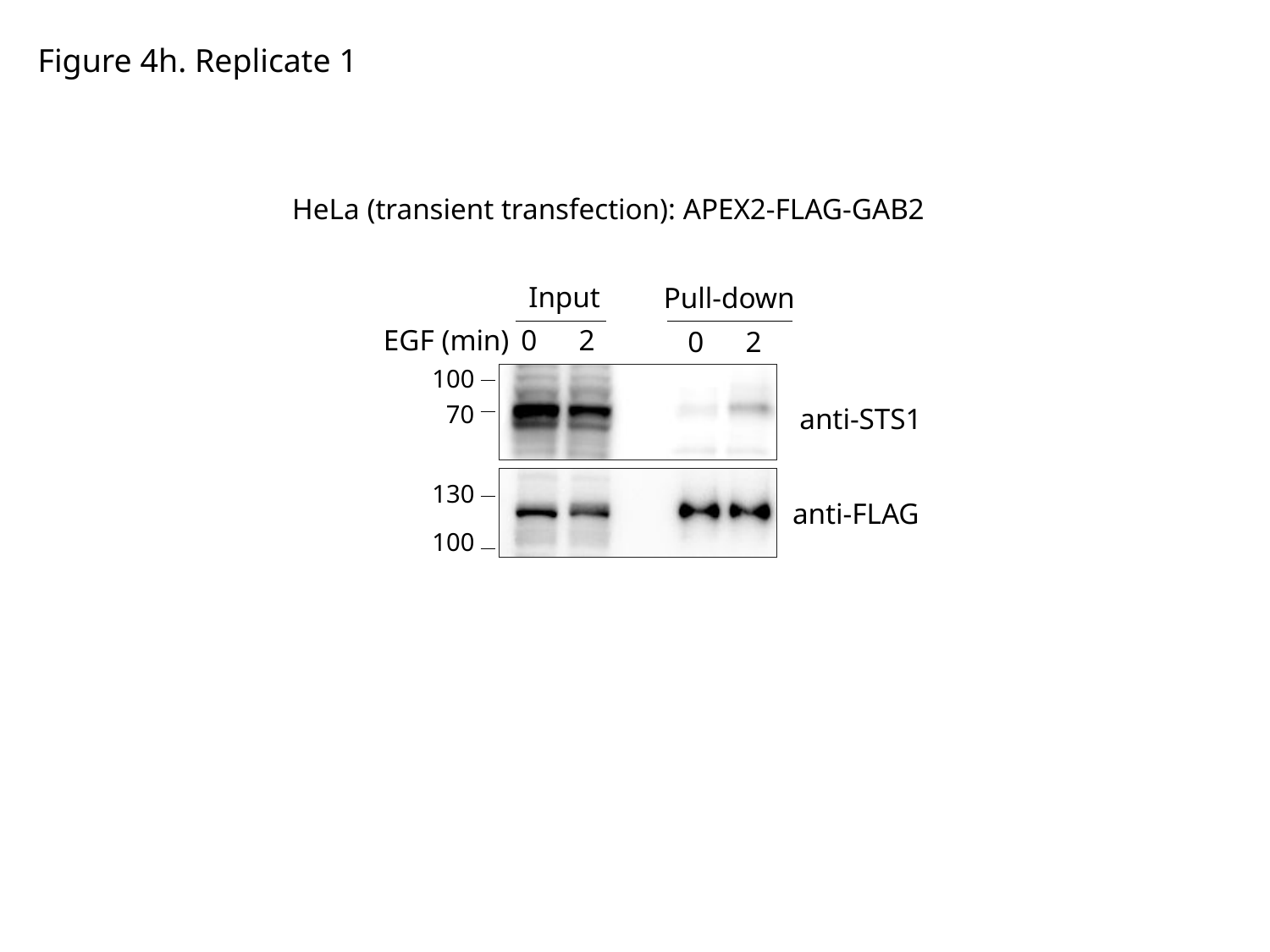

Figure 4h. Replicate 1
HeLa (transient transfection): APEX2-FLAG-GAB2
Input
Pull-down
EGF (min)
0
2
0
2
100
70
anti-STS1
130
anti-FLAG
100

## Slide 2
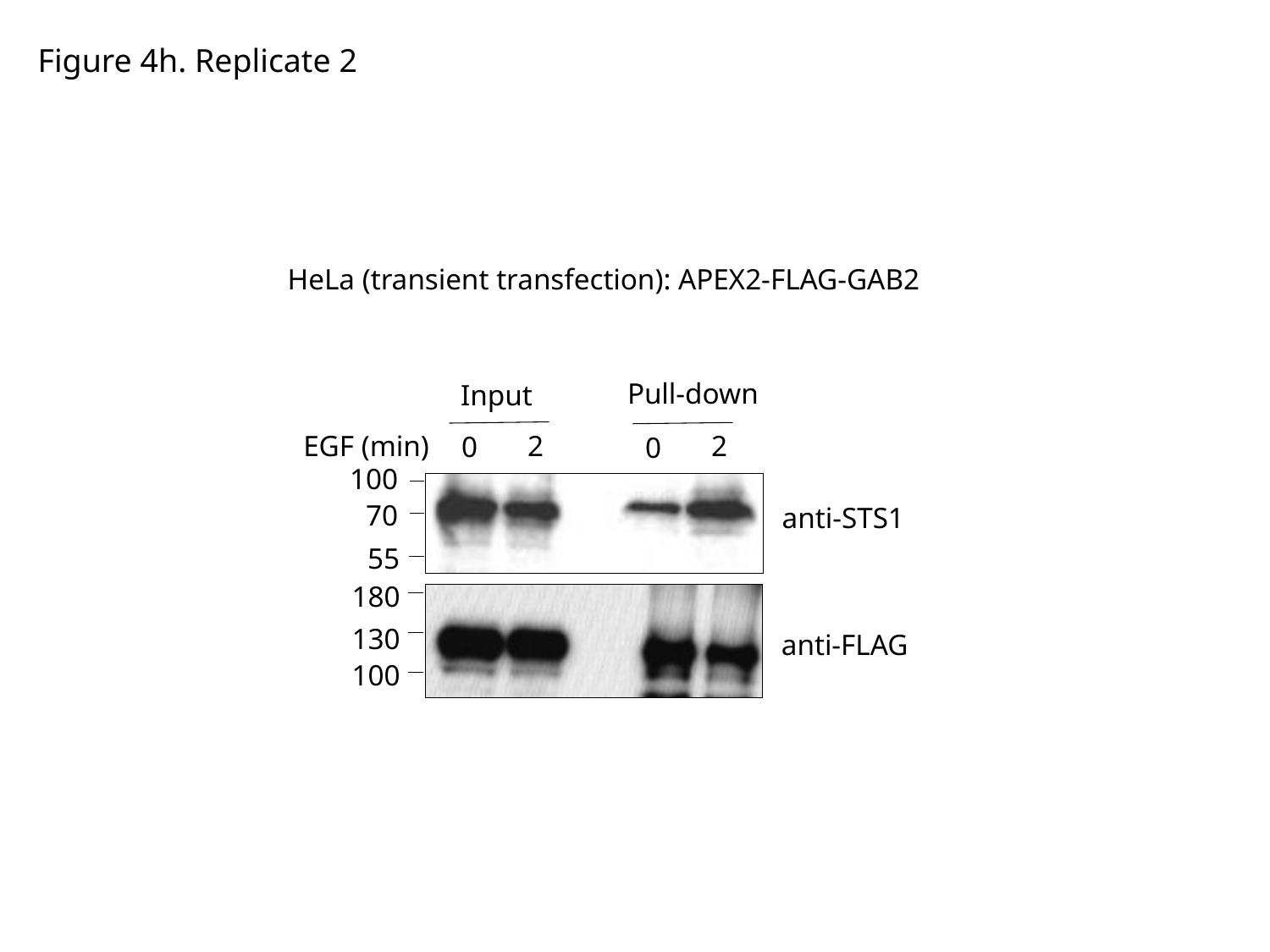

Figure 4h. Replicate 2
HeLa (transient transfection): APEX2-FLAG-GAB2
Pull-down
Input
2
EGF (min)
2
0
0
100
70
anti-STS1
55
180
130
anti-FLAG
100

## Slide 3
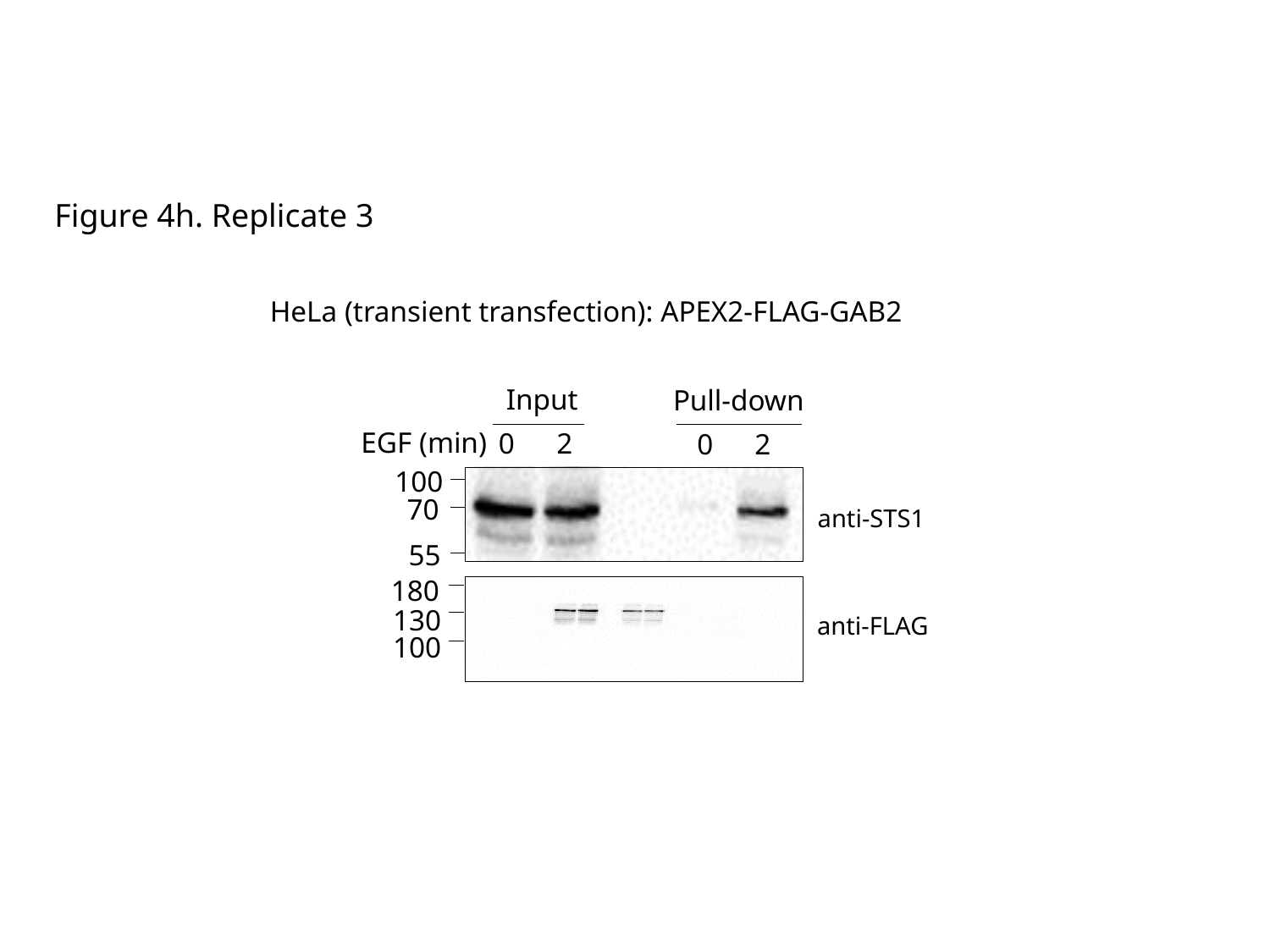

Figure 4h. Replicate 3
HeLa (transient transfection): APEX2-FLAG-GAB2
Input
Pull-down
EGF (min)
0
2
0
2
100
70
anti-STS1
55
180
130
anti-FLAG
100

## Slide 4
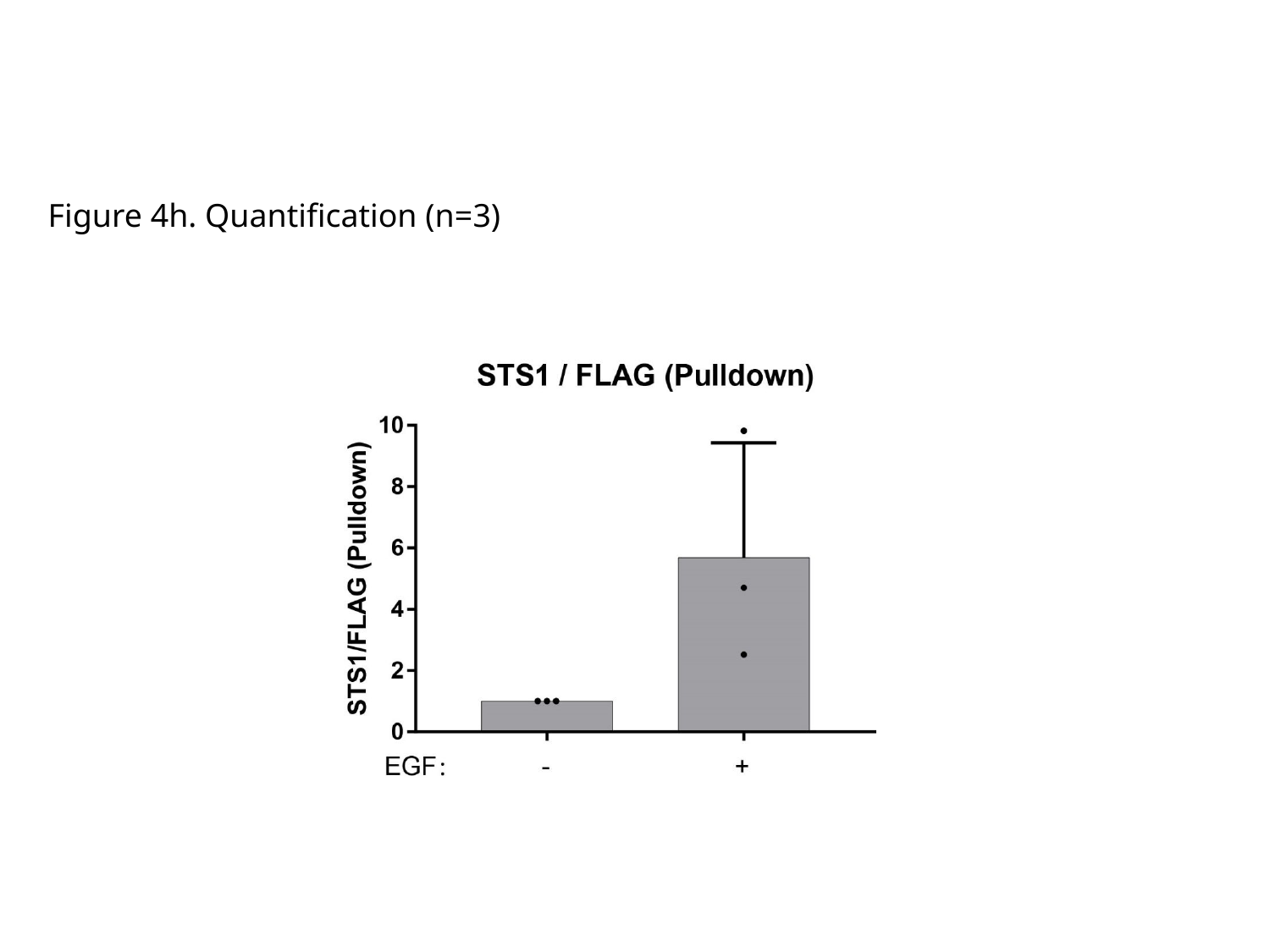

Figure 4h. Quantification (n=3)
