# Supplementary material for: Spatiotemporal profiling of cytosolic signaling complexes in living cells by selective proximity proteomics
Source: Nat Commun. 2021 Jan 4;12:71. doi: 10.1038/s41467-020-20367-x (PMC7782698; doi:10.1038/s41467-020-20367-x)
Supplement: Supplementary file 16 — Source Data [file 41467_2020_20367_MOESM16_ESM.zip › NCOMMS-20-22505C_sd/WB and IF_Replicates and Quantification/Figure 6d/Three replicates.pptx]

## Slide 1
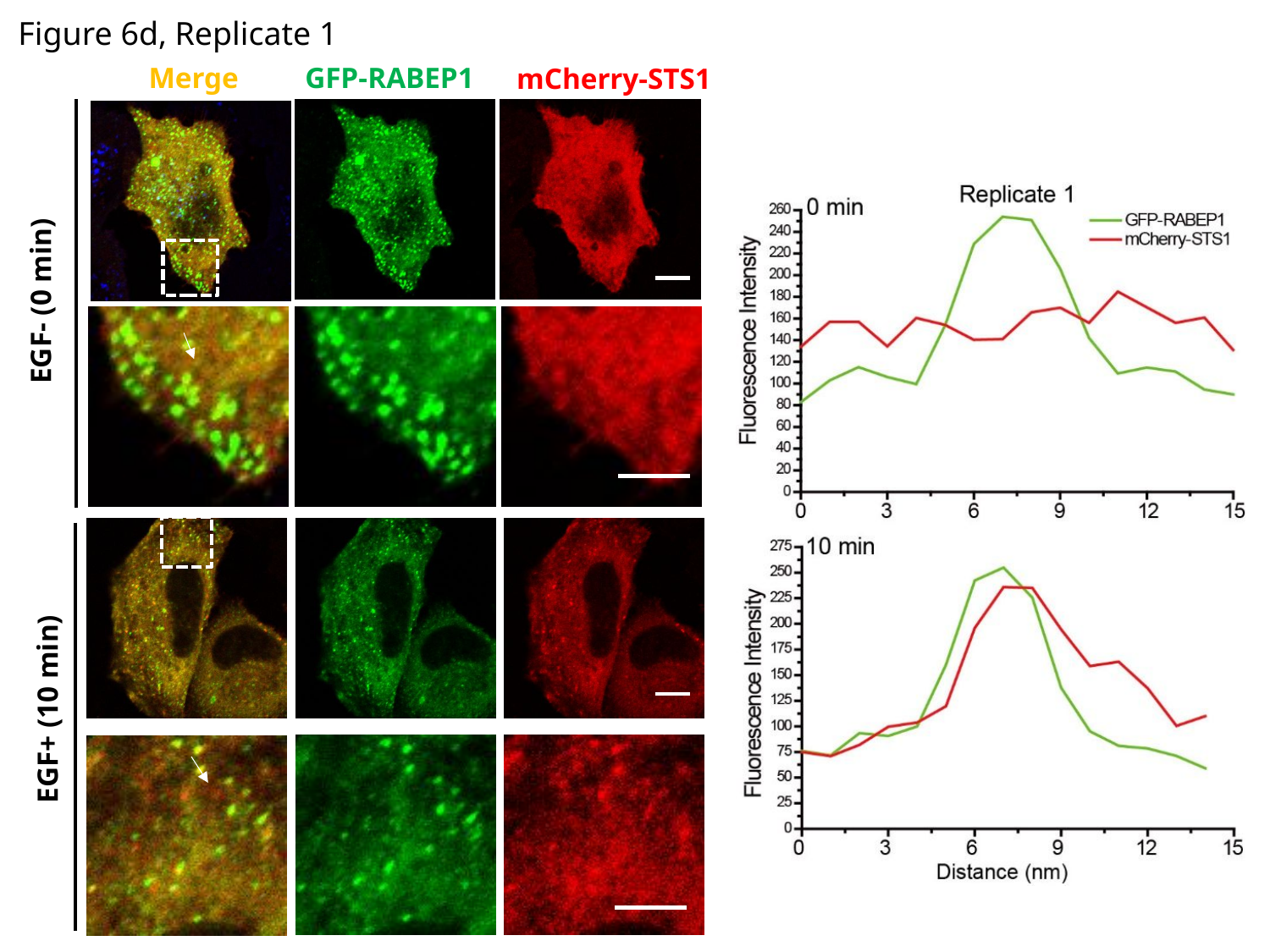

Figure 6d, Replicate 1
Merge
GFP-RABEP1
mCherry-STS1
EGF- (0 min)
EGF+ (10 min)

## Slide 2
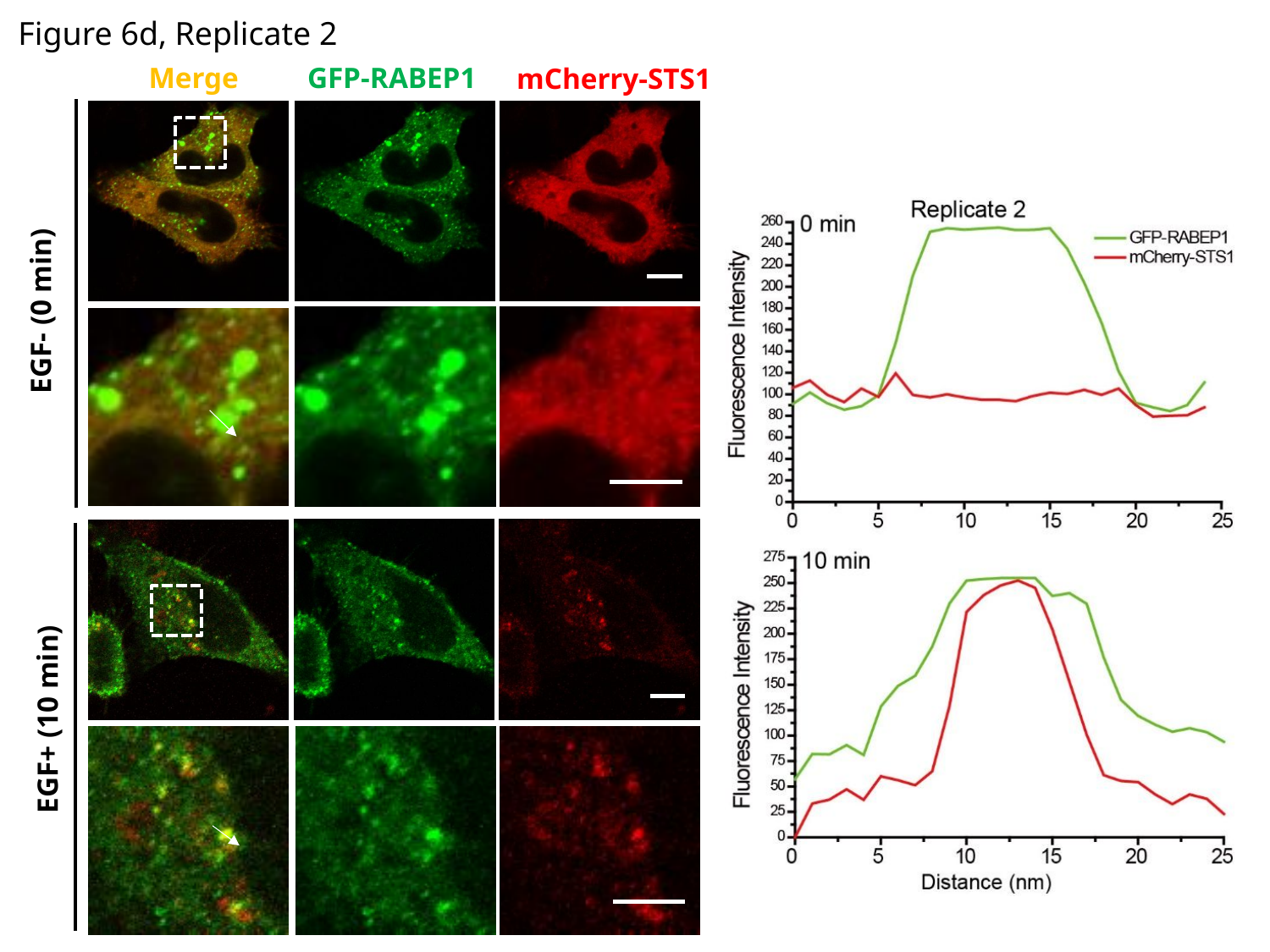

Figure 6d, Replicate 2
Merge
GFP-RABEP1
mCherry-STS1
EGF- (0 min)
EGF+ (10 min)

## Slide 3
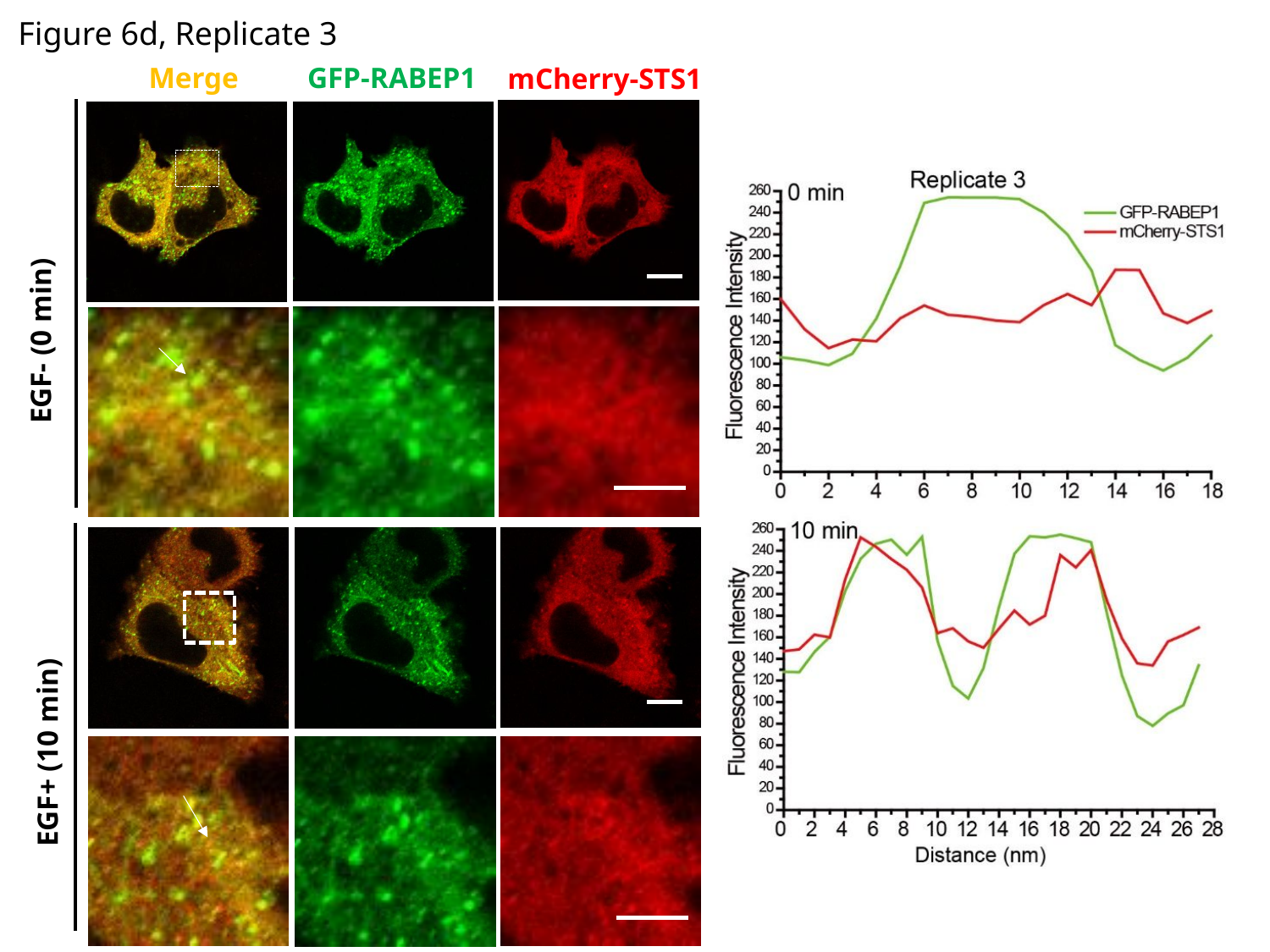

Figure 6d, Replicate 3
Merge
GFP-RABEP1
mCherry-STS1
EGF- (0 min)
EGF+ (10 min)
