# Supplementary material for: Spatiotemporal profiling of cytosolic signaling complexes in living cells by selective proximity proteomics
Source: Nat Commun. 2021 Jan 4;12:71. doi: 10.1038/s41467-020-20367-x (PMC7782698; doi:10.1038/s41467-020-20367-x)
Supplement: Supplementary file 16 — Source Data [file 41467_2020_20367_MOESM16_ESM.zip › NCOMMS-20-22505C_sd/WB and IF_Replicates and Quantification/Supplementary Figure 3a/Three replicates.pptx]

## Slide 1
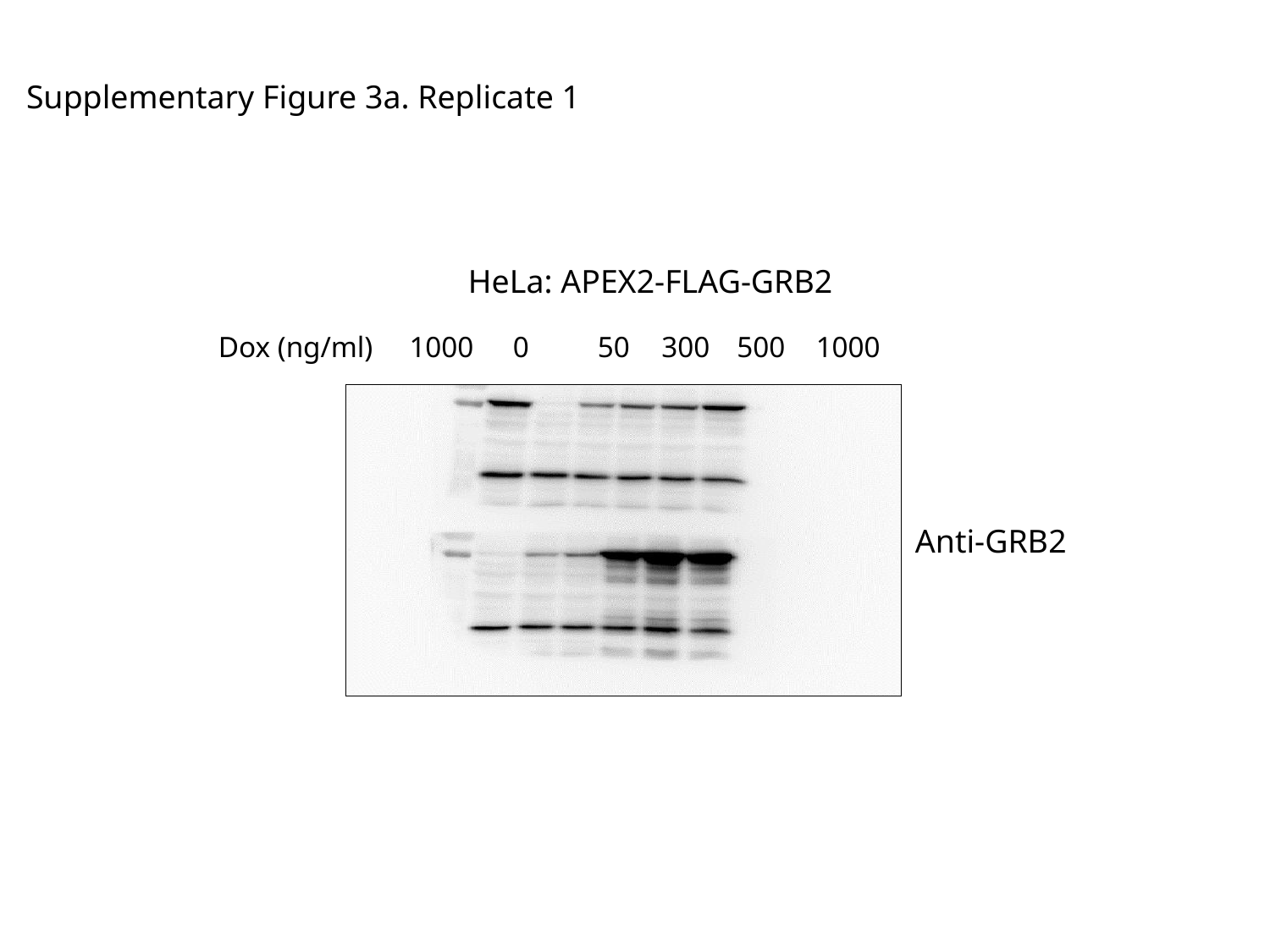

Supplementary Figure 3a. Replicate 1
HeLa: APEX2-FLAG-GRB2
Dox (ng/ml)
1000
0
50
300
500
1000
Anti-GRB2

## Slide 2
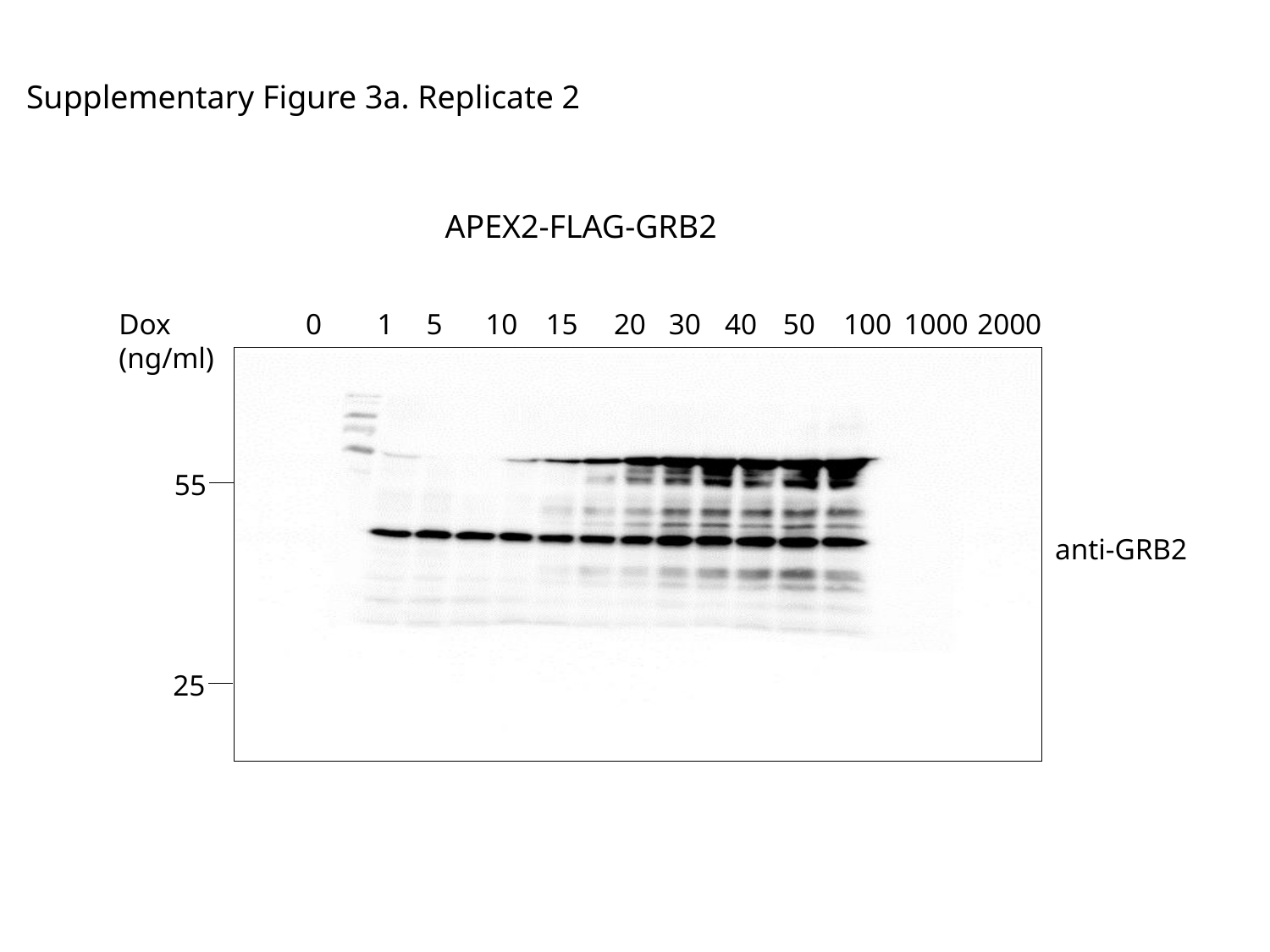

Supplementary Figure 3a. Replicate 2
APEX2-FLAG-GRB2
Dox (ng/ml)
0
1
5
10
15
20
30
40
50
100
1000
2000
55
anti-GRB2
25

## Slide 3
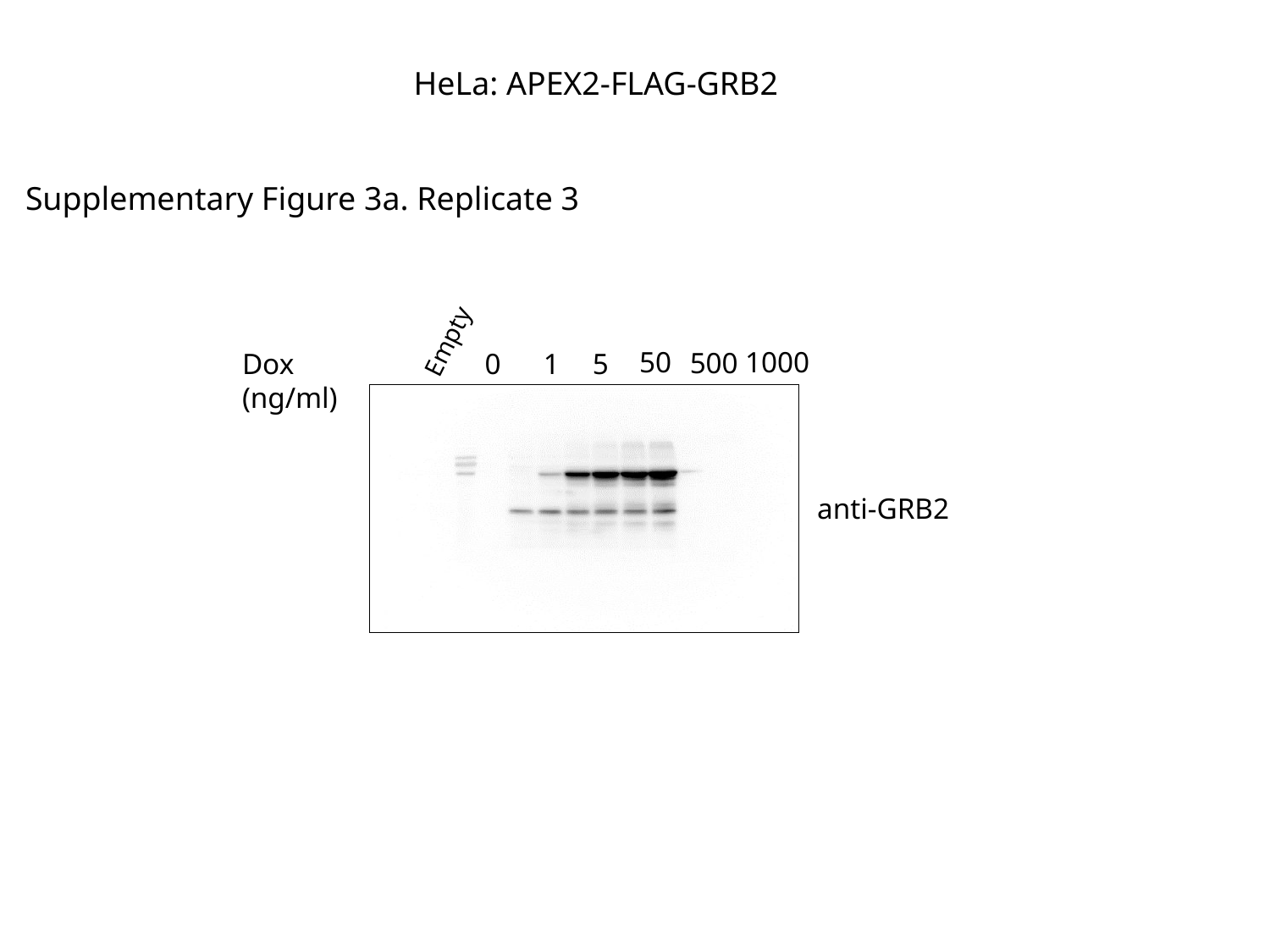

HeLa: APEX2-FLAG-GRB2
Supplementary Figure 3a. Replicate 3
Empty
50
1000
500
Dox (ng/ml)
0
1
5
anti-GRB2

## Slide 4
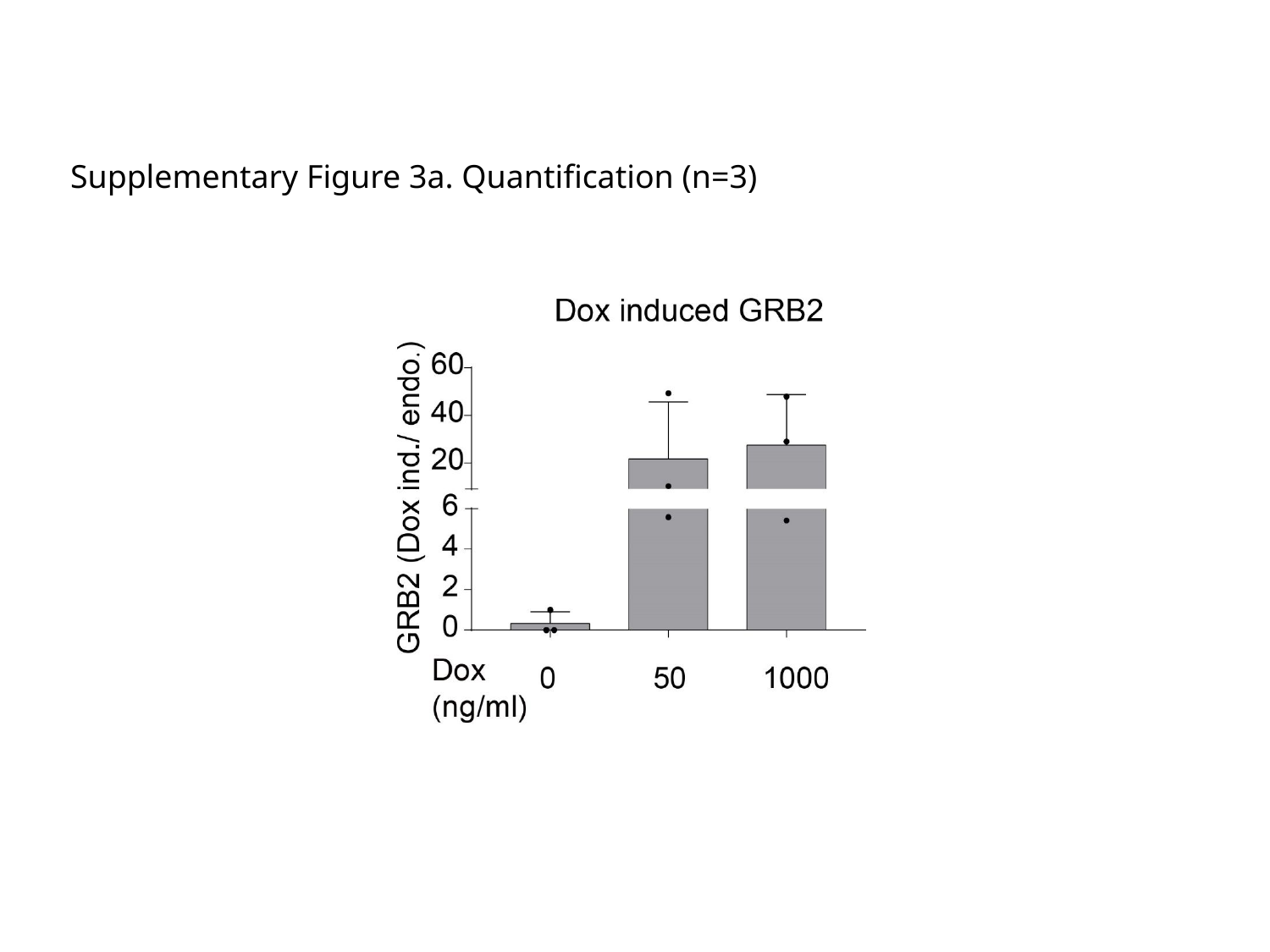

Supplementary Figure 3a. Quantification (n=3)
