# Supplementary material for: Spatiotemporal profiling of cytosolic signaling complexes in living cells by selective proximity proteomics
Source: Nat Commun. 2021 Jan 4;12:71. doi: 10.1038/s41467-020-20367-x (PMC7782698; doi:10.1038/s41467-020-20367-x)
Supplement: Supplementary file 16 — Source Data [file 41467_2020_20367_MOESM16_ESM.zip › NCOMMS-20-22505C_sd/WB and IF_Replicates and Quantification/Supplementary Figure 3b/Three replicates.pptx]

## Slide 1
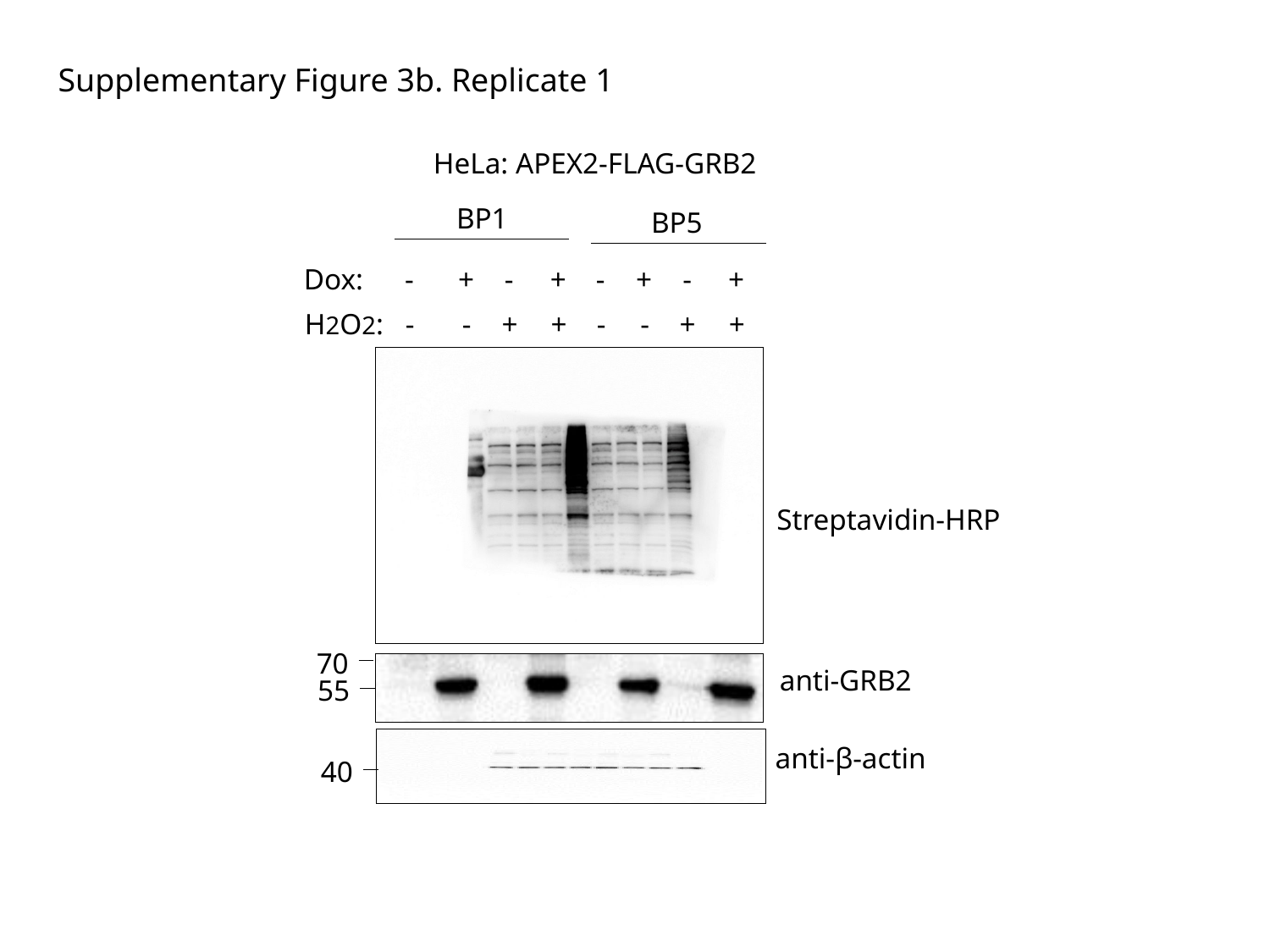

Supplementary Figure 3b. Replicate 1
HeLa: APEX2-FLAG-GRB2
BP1
BP5
Dox:
-
+
-
+
-
+
-
+
H2O2:
-
-
+
+
-
-
+
+
Streptavidin-HRP
70
anti-GRB2
55
anti-β-actin
40

## Slide 2
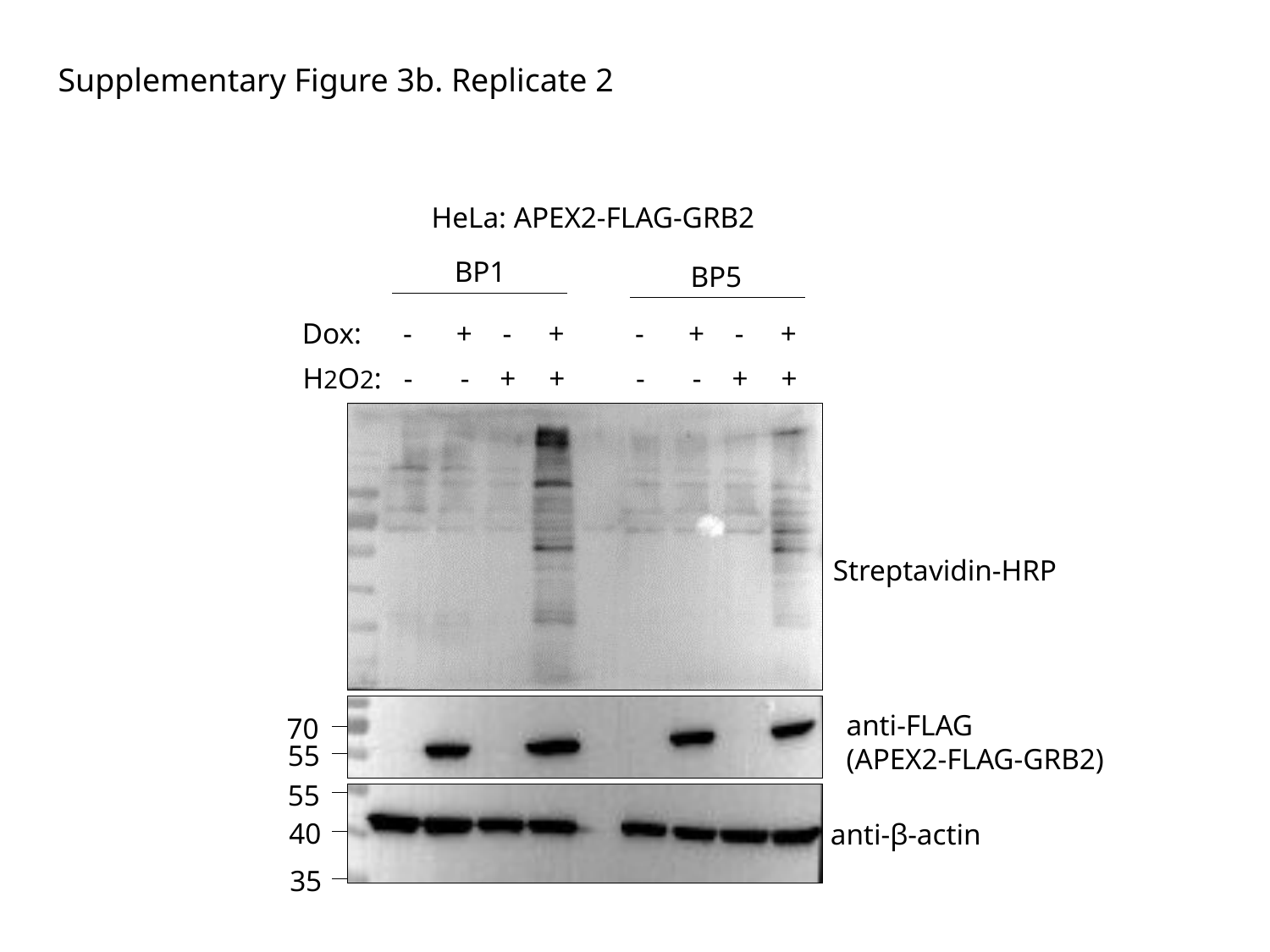

Supplementary Figure 3b. Replicate 2
HeLa: APEX2-FLAG-GRB2
BP1
BP5
Dox:
-
+
-
+
-
+
-
+
H2O2:
-
-
+
+
-
-
+
+
Streptavidin-HRP
anti-FLAG
(APEX2-FLAG-GRB2)
70
55
55
40
anti-β-actin
35

## Slide 3
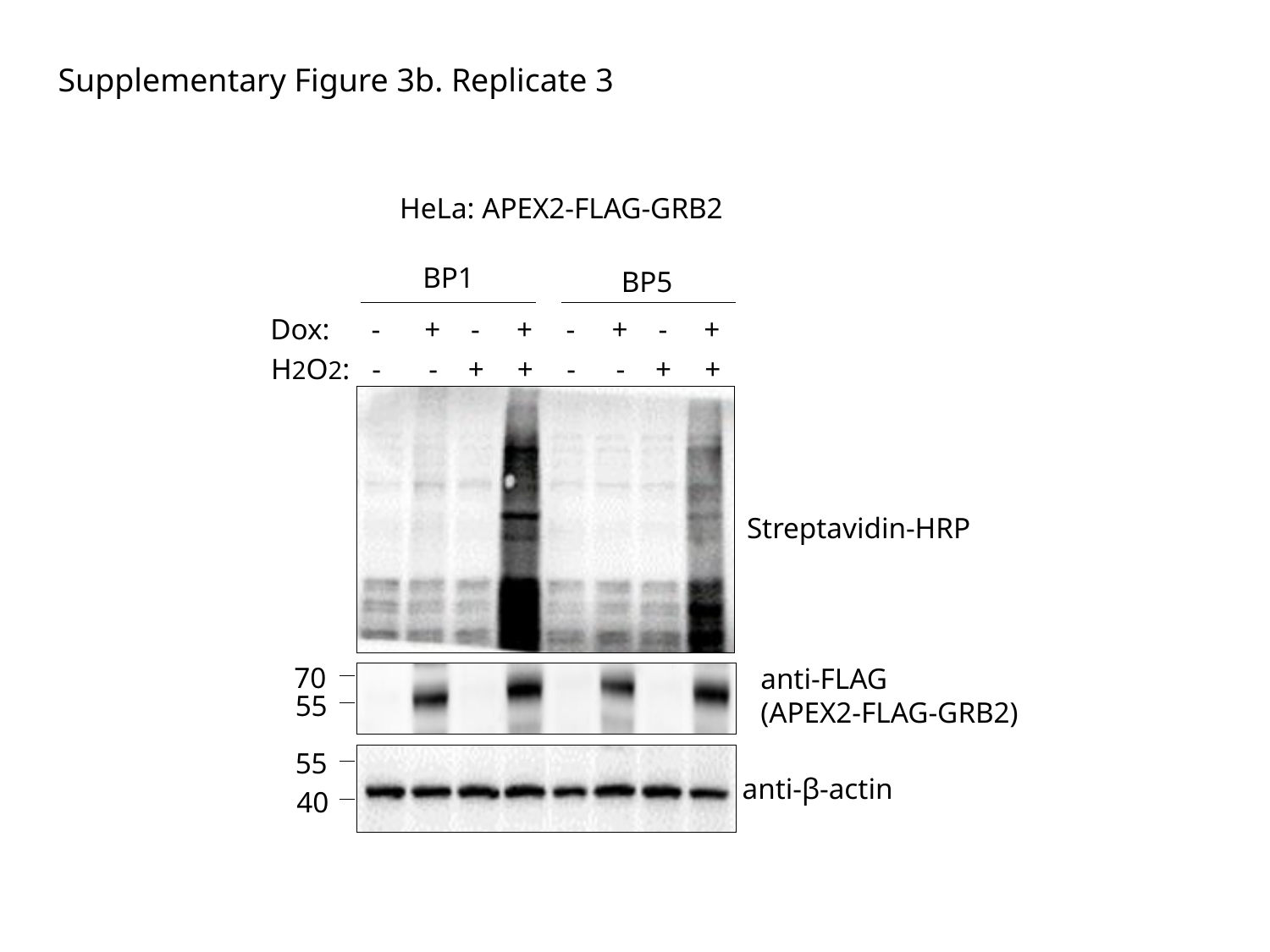

Supplementary Figure 3b. Replicate 3
HeLa: APEX2-FLAG-GRB2
BP1
BP5
Dox:
-
+
-
+
-
+
-
+
H2O2:
-
-
+
+
-
-
+
+
Streptavidin-HRP
70
anti-FLAG
(APEX2-FLAG-GRB2)
55
55
anti-β-actin
40

## Slide 4
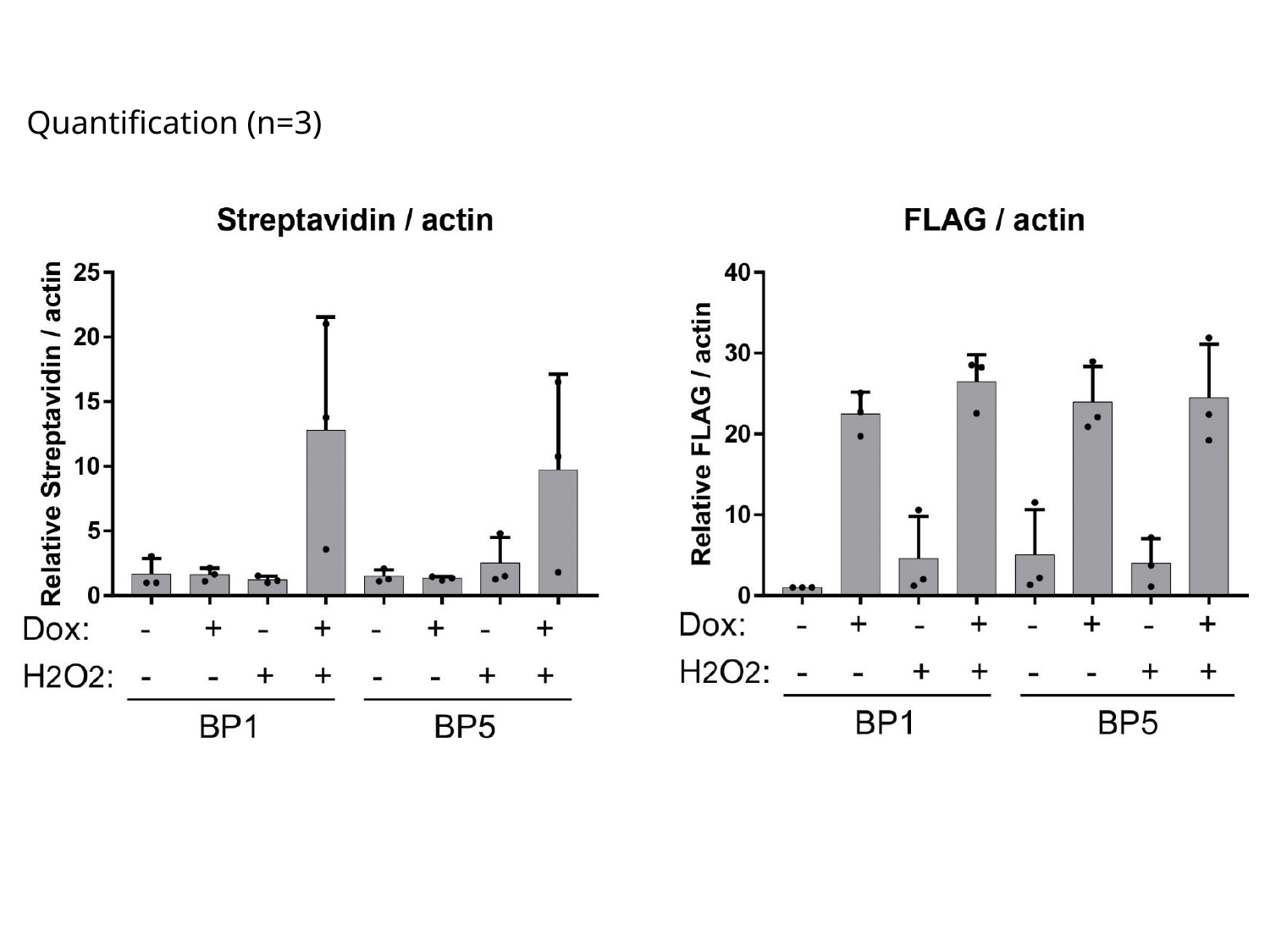

Quantification (n=3)
