# Supplementary material for: Spatiotemporal profiling of cytosolic signaling complexes in living cells by selective proximity proteomics
Source: Nat Commun. 2021 Jan 4;12:71. doi: 10.1038/s41467-020-20367-x (PMC7782698; doi:10.1038/s41467-020-20367-x)
Supplement: Supplementary file 16 — Source Data [file 41467_2020_20367_MOESM16_ESM.zip › NCOMMS-20-22505C_sd/WB and IF_Replicates and Quantification/Supplementary Figure 3c/Three replicates.pptx]

## Slide 1
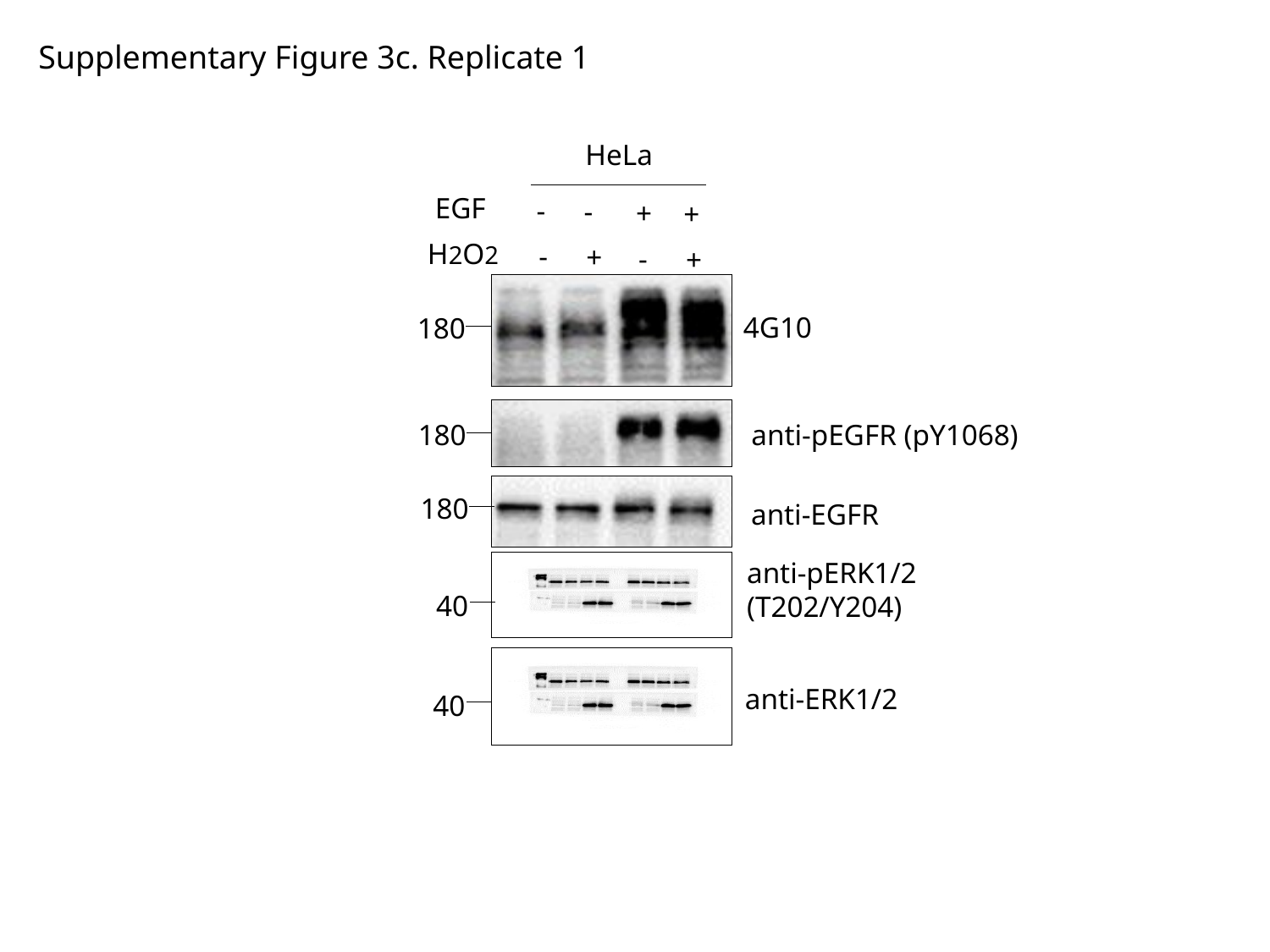

Supplementary Figure 3c. Replicate 1
HeLa
EGF
-
-
+
+
H2O2
-
+
-
+
4G10
180
anti-pEGFR (pY1068)
180
180
anti-EGFR
anti-pERK1/2
(T202/Y204)
40
anti-ERK1/2
40

## Slide 2
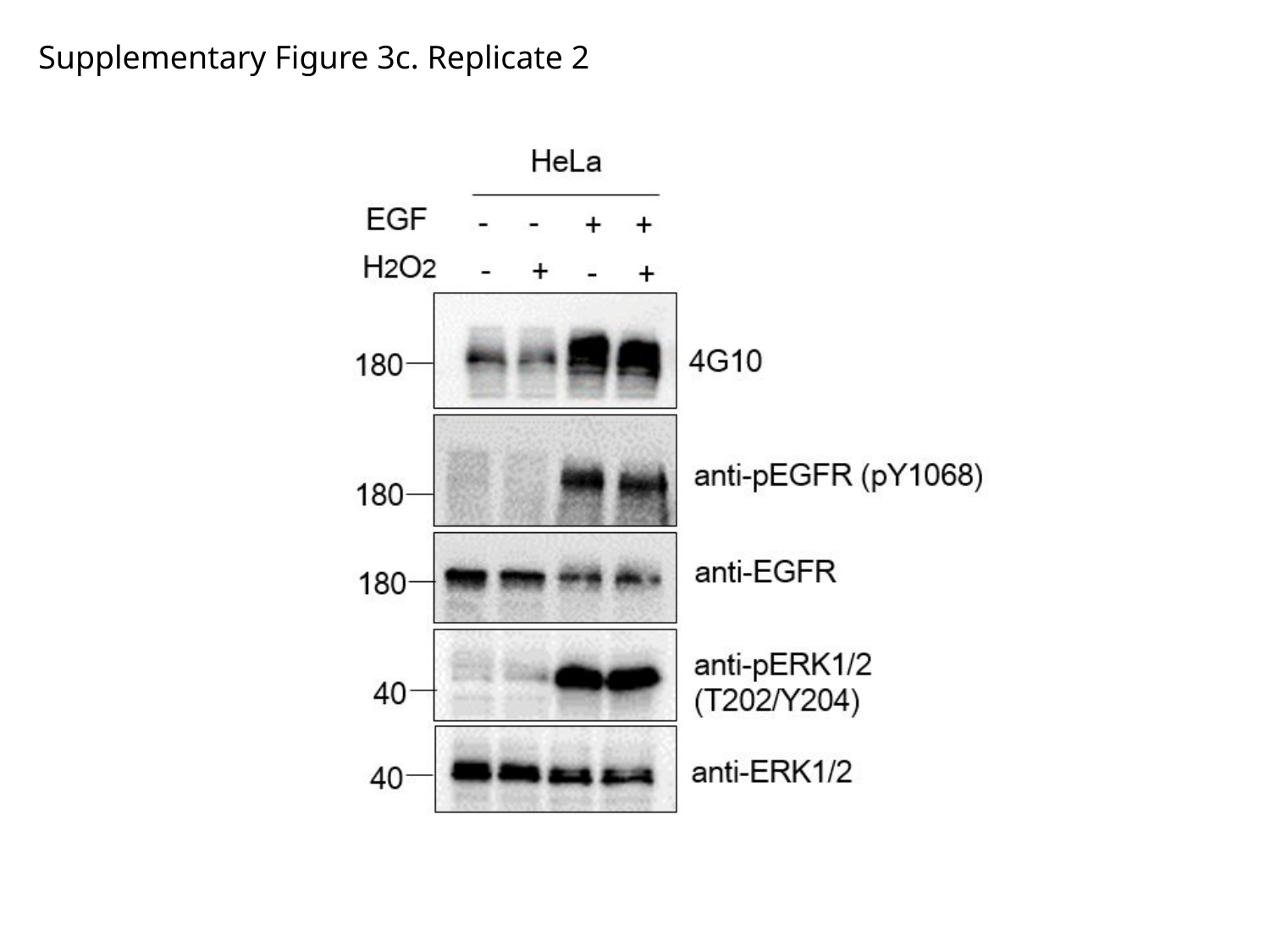

Supplementary Figure 3c. Replicate 2
HeLa
EGF
-
-
+
+
H2O2
-
+
-
+
4G10
180
anti-pEGFR (pY1068)
180
anti-EGFR
180
anti-pERK1/2
(T202/Y204)
40
anti-ERK1/2
40

## Slide 3
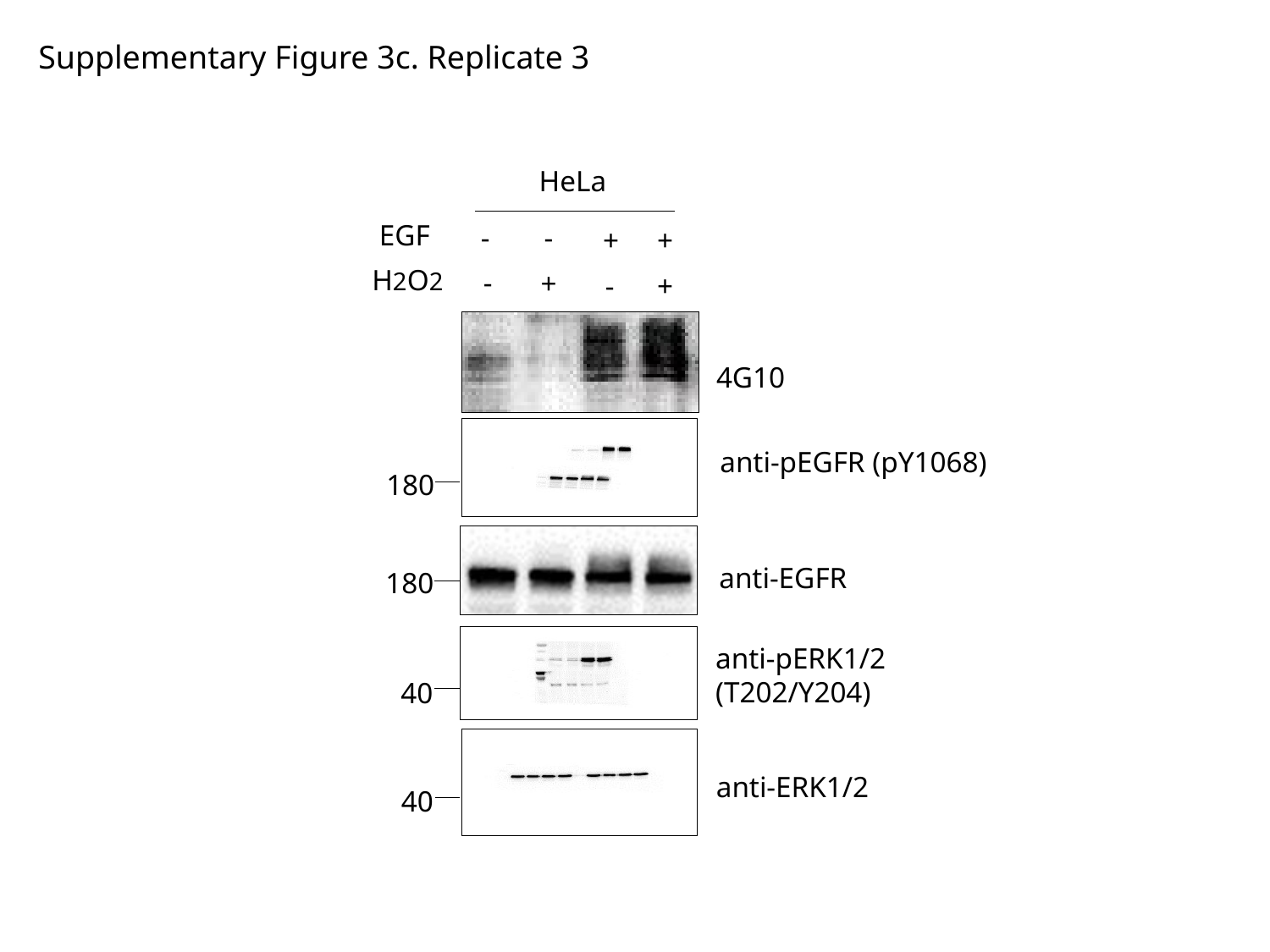

Supplementary Figure 3c. Replicate 3
HeLa
EGF
-
-
+
+
H2O2
-
+
-
+
4G10
anti-pEGFR (pY1068)
180
anti-EGFR
180
anti-pERK1/2
(T202/Y204)
40
anti-ERK1/2
40

## Slide 4
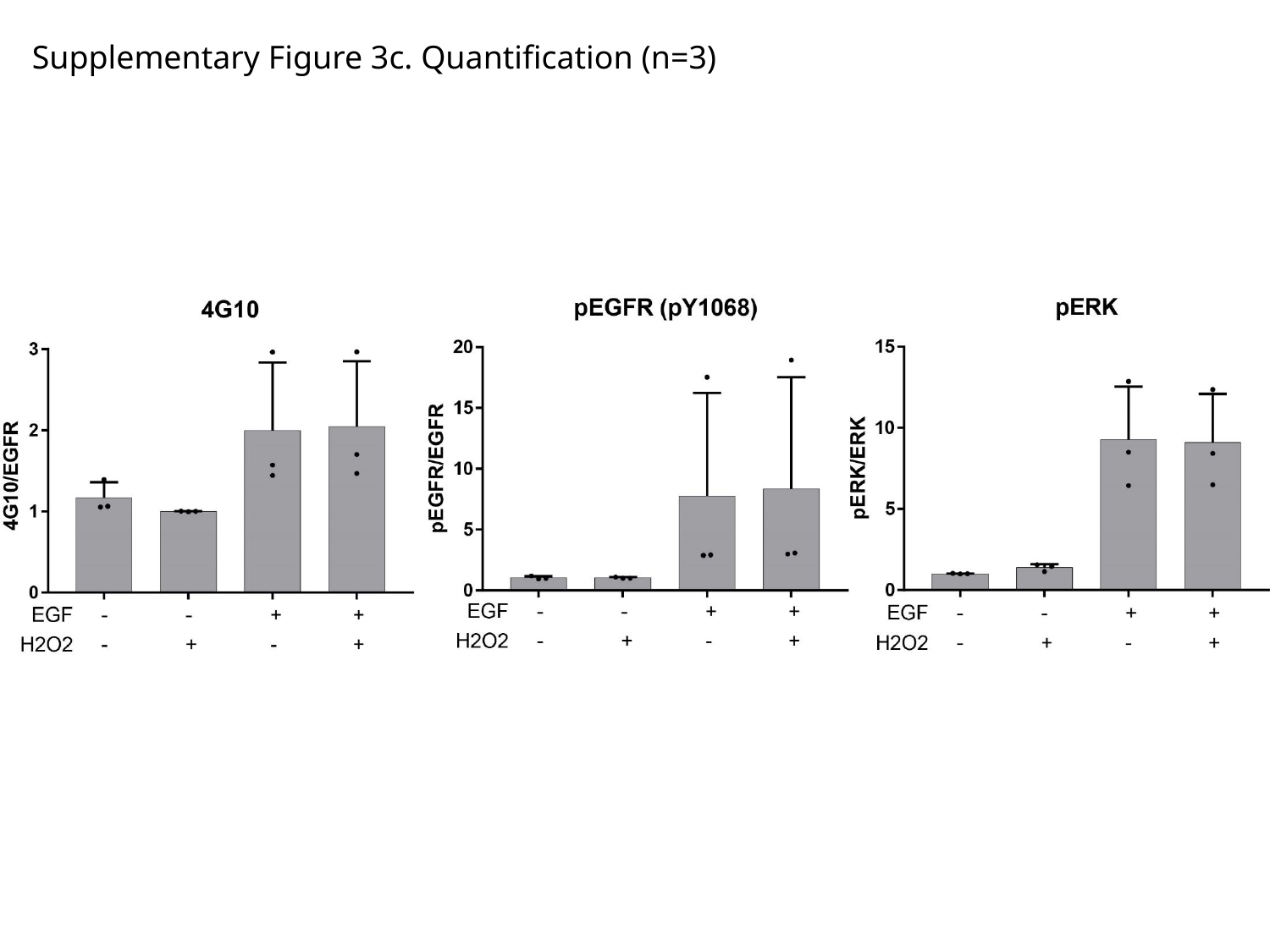

Supplementary Figure 3c. Quantification (n=3)
