# Supplementary material for: Spatiotemporal profiling of cytosolic signaling complexes in living cells by selective proximity proteomics
Source: Nat Commun. 2021 Jan 4;12:71. doi: 10.1038/s41467-020-20367-x (PMC7782698; doi:10.1038/s41467-020-20367-x)
Supplement: Supplementary file 16 — Source Data [file 41467_2020_20367_MOESM16_ESM.zip › NCOMMS-20-22505C_sd/WB and IF_Replicates and Quantification/Supplementary Figure 3g/Three replicates.pptx]

## Slide 1
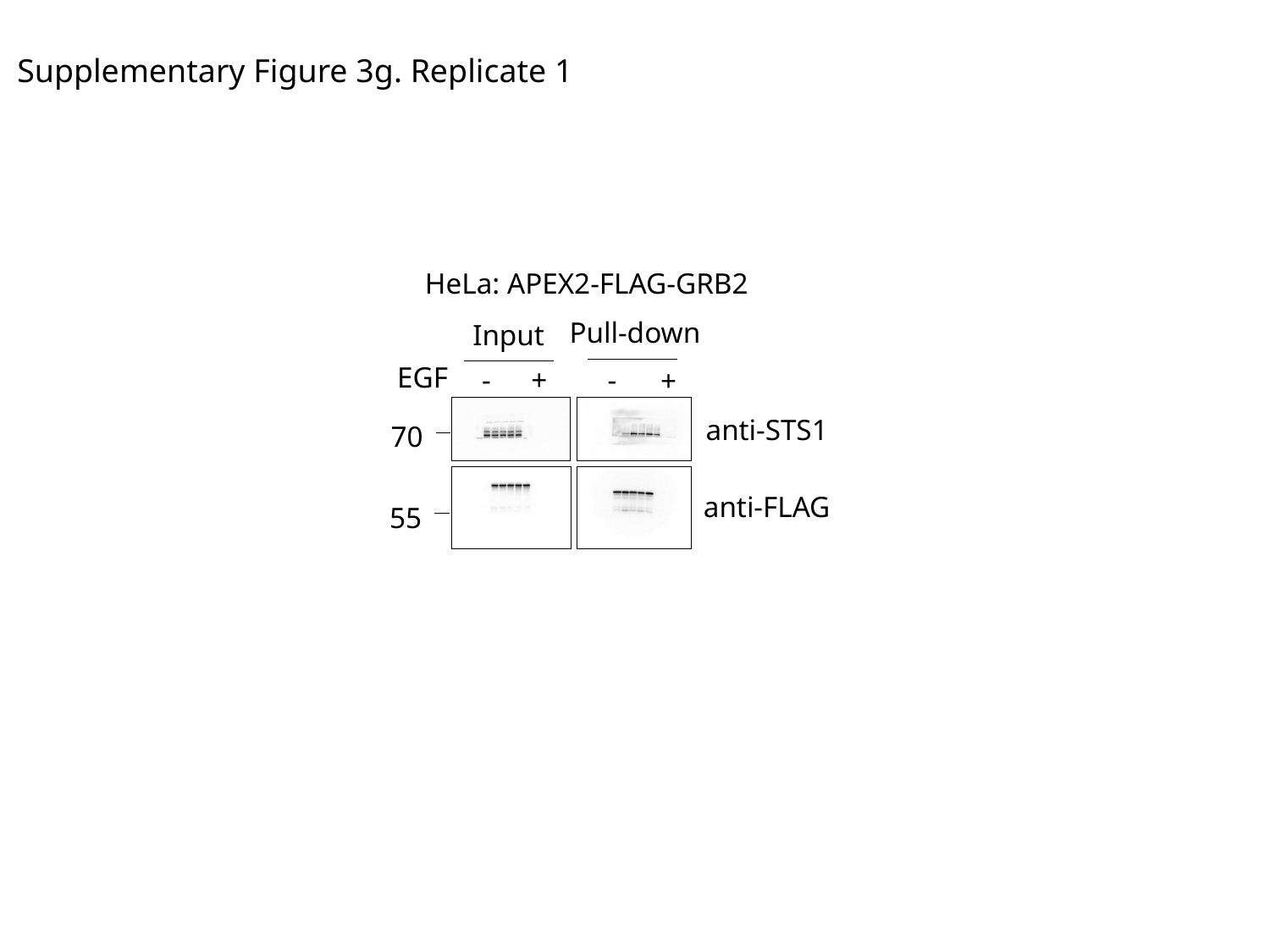

Supplementary Figure 3g. Replicate 1
HeLa: APEX2-FLAG-GRB2
Pull-down
Input
EGF
-
+
-
+
anti-STS1
70
anti-FLAG
55

## Slide 2
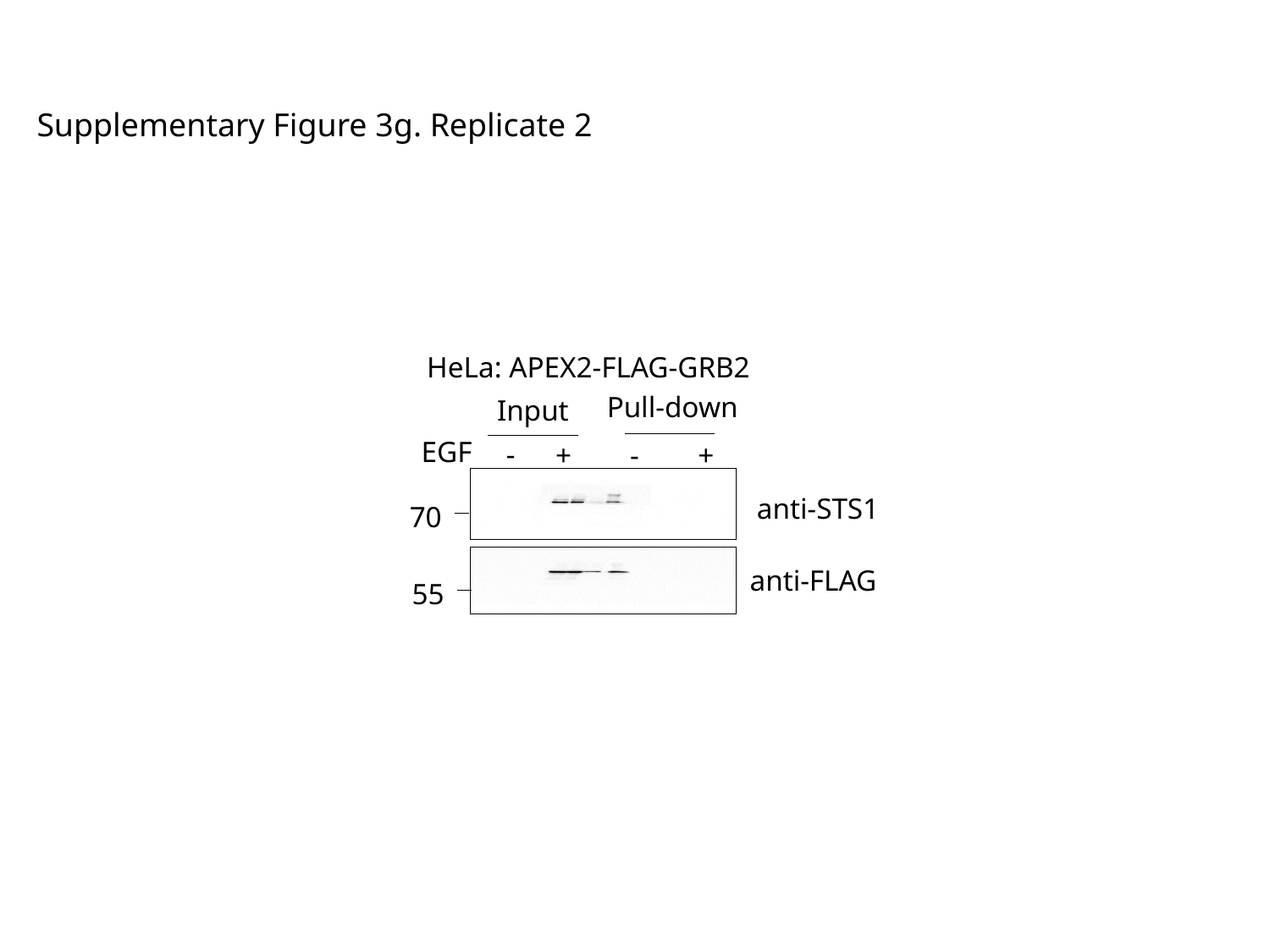

Supplementary Figure 3g. Replicate 2
HeLa: APEX2-FLAG-GRB2
Pull-down
Input
EGF
-
+
-
+
anti-STS1
70
anti-FLAG
55

## Slide 3
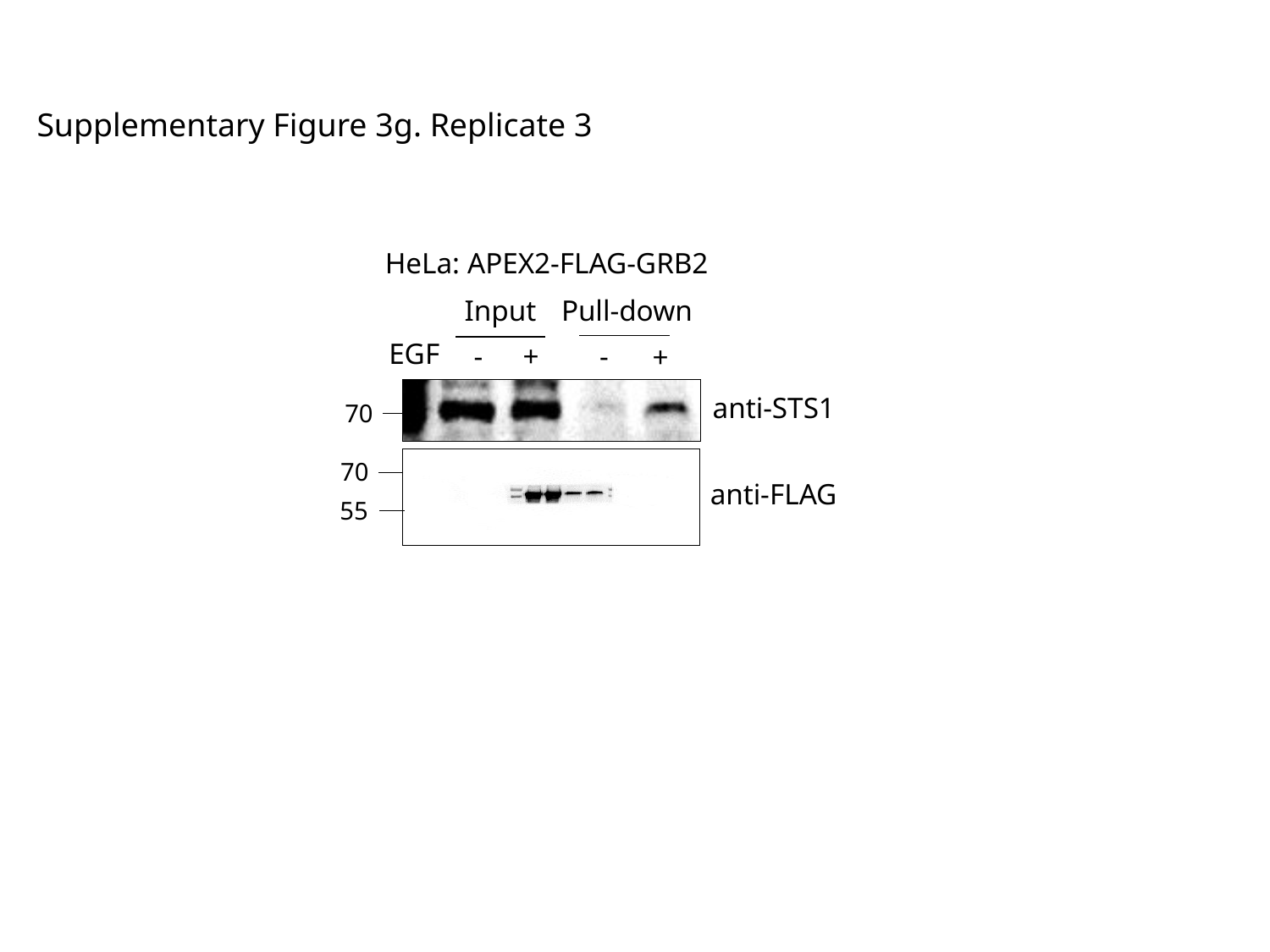

Supplementary Figure 3g. Replicate 3
HeLa: APEX2-FLAG-GRB2
Input
Pull-down
EGF
-
+
-
+
anti-STS1
70
70
anti-FLAG
55

## Slide 4
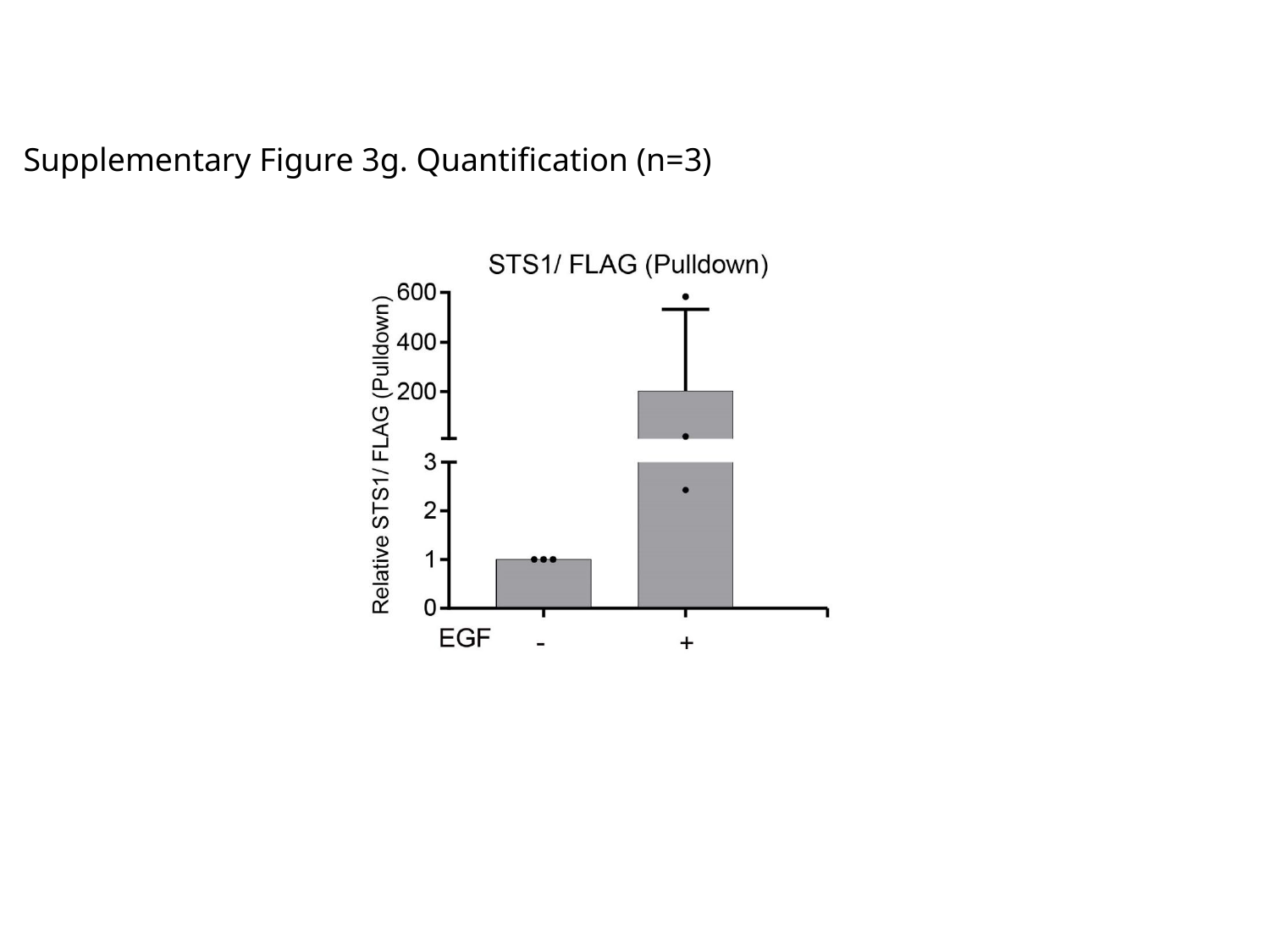

Supplementary Figure 3g. Quantification (n=3)
