# Supplementary material for: Spatiotemporal profiling of cytosolic signaling complexes in living cells by selective proximity proteomics
Source: Nat Commun. 2021 Jan 4;12:71. doi: 10.1038/s41467-020-20367-x (PMC7782698; doi:10.1038/s41467-020-20367-x)
Supplement: Supplementary file 16 — Source Data [file 41467_2020_20367_MOESM16_ESM.zip › NCOMMS-20-22505C_sd/WB and IF_Replicates and Quantification/Supplementary Figure 3h/Three replicates.pptx]

## Slide 1
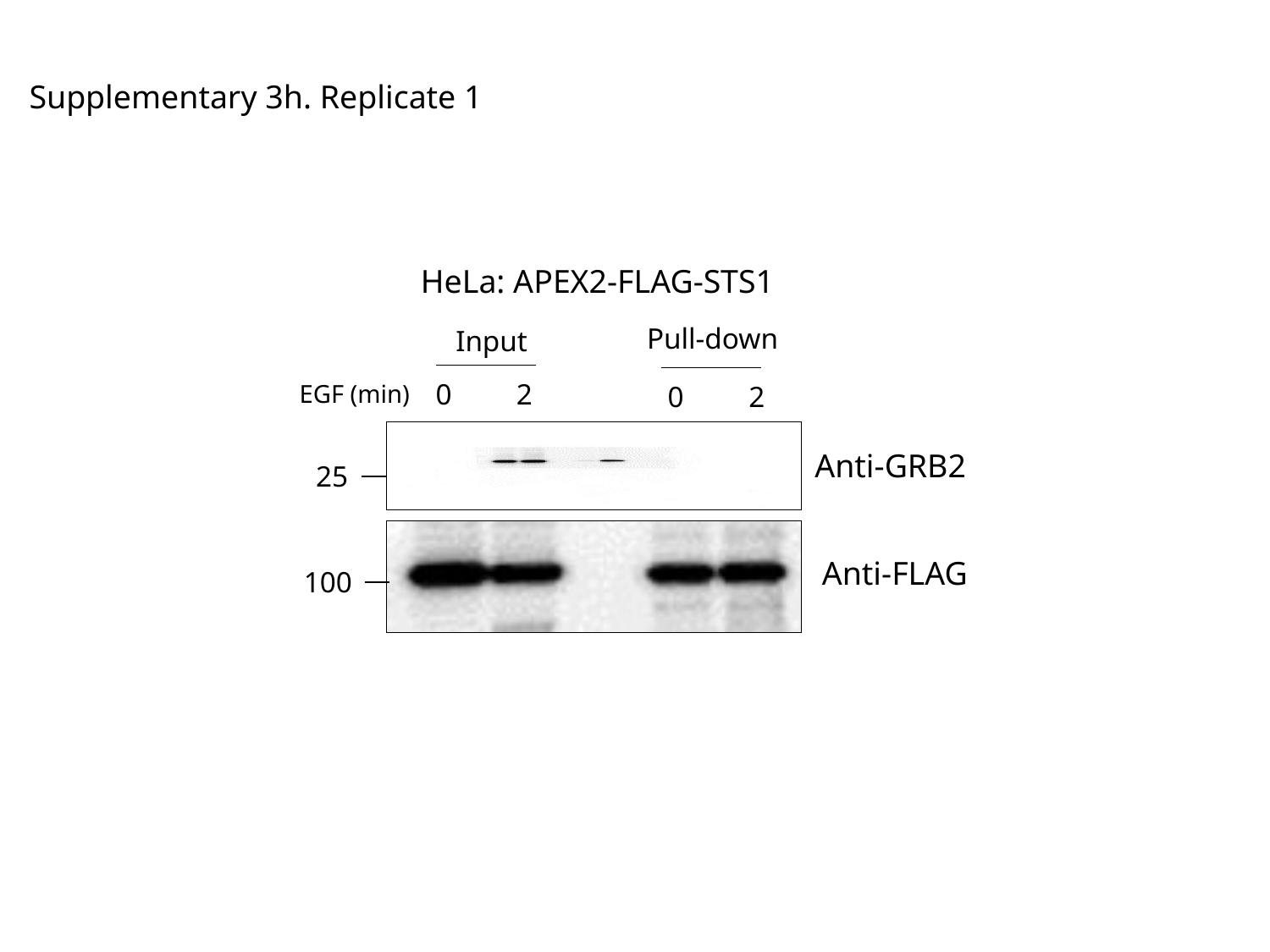

Supplementary 3h. Replicate 1
HeLa: APEX2-FLAG-STS1
Pull-down
Input
0
2
EGF (min)
0
2
Anti-GRB2
25
Anti-FLAG
100

## Slide 2
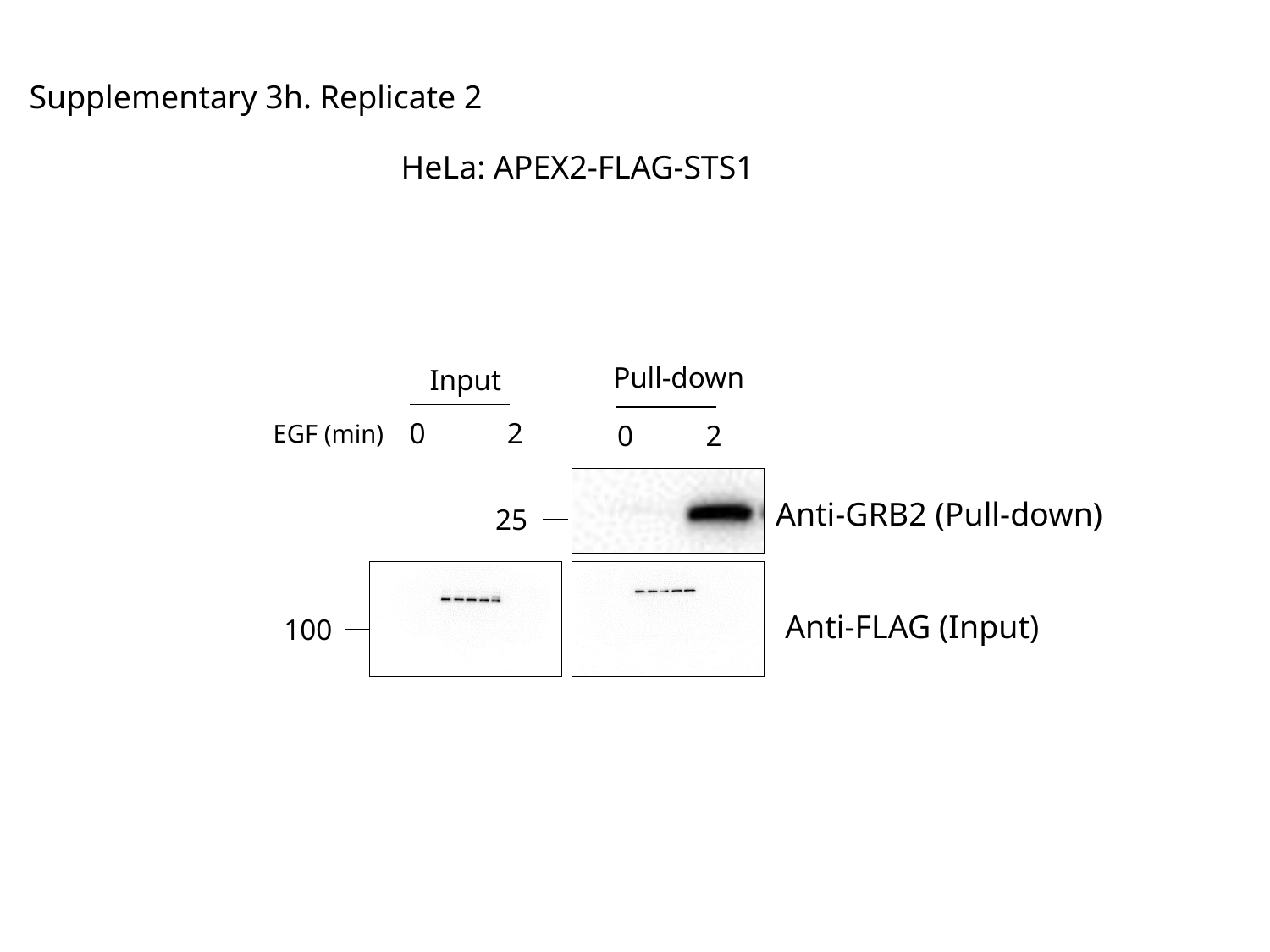

Supplementary 3h. Replicate 2
HeLa: APEX2-FLAG-STS1
Pull-down
Input
0
2
EGF (min)
0
2
Anti-GRB2 (Pull-down)
25
Anti-FLAG (Input)
100

## Slide 3
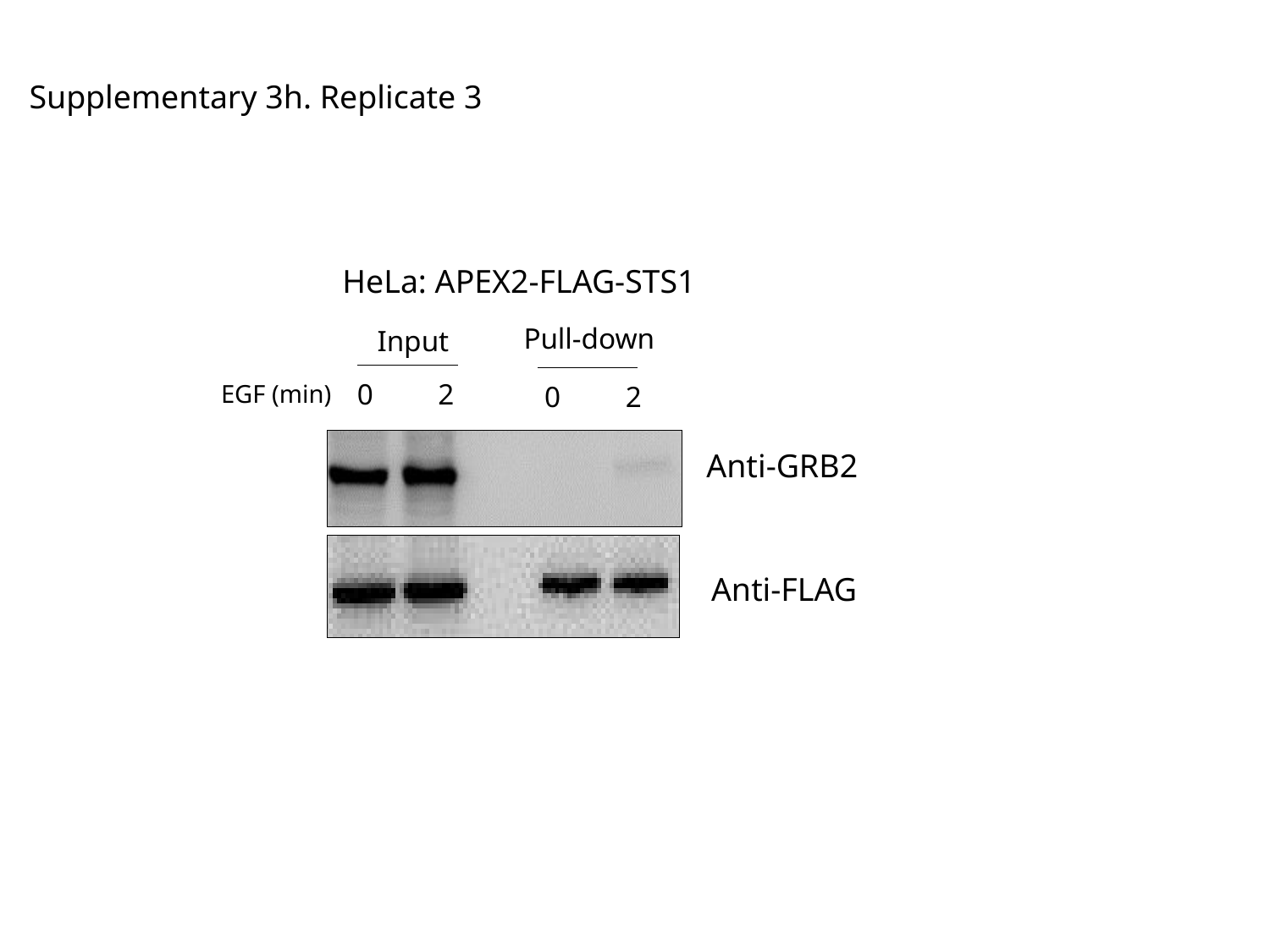

Supplementary 3h. Replicate 3
HeLa: APEX2-FLAG-STS1
Pull-down
Input
0
2
EGF (min)
0
2
Anti-GRB2
Anti-FLAG

## Slide 4
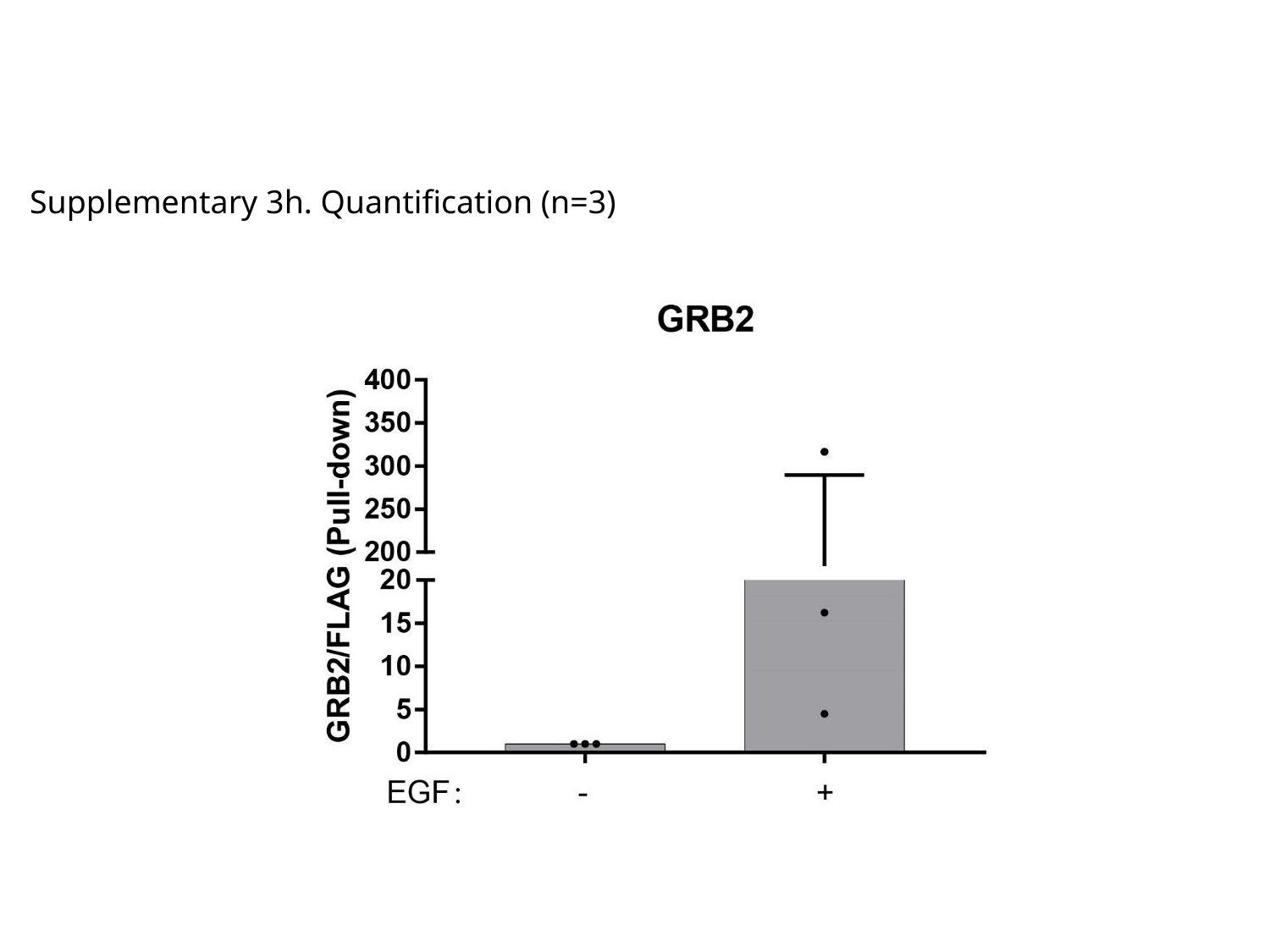

Supplementary 3h. Quantification (n=3)
