# Supplementary material for: Spatiotemporal profiling of cytosolic signaling complexes in living cells by selective proximity proteomics
Source: Nat Commun. 2021 Jan 4;12:71. doi: 10.1038/s41467-020-20367-x (PMC7782698; doi:10.1038/s41467-020-20367-x)
Supplement: Supplementary file 16 — Source Data [file 41467_2020_20367_MOESM16_ESM.zip › NCOMMS-20-22505C_sd/WB and IF_Replicates and Quantification/Supplementary Figure 7a/Three replicates.pptx]

## Slide 1
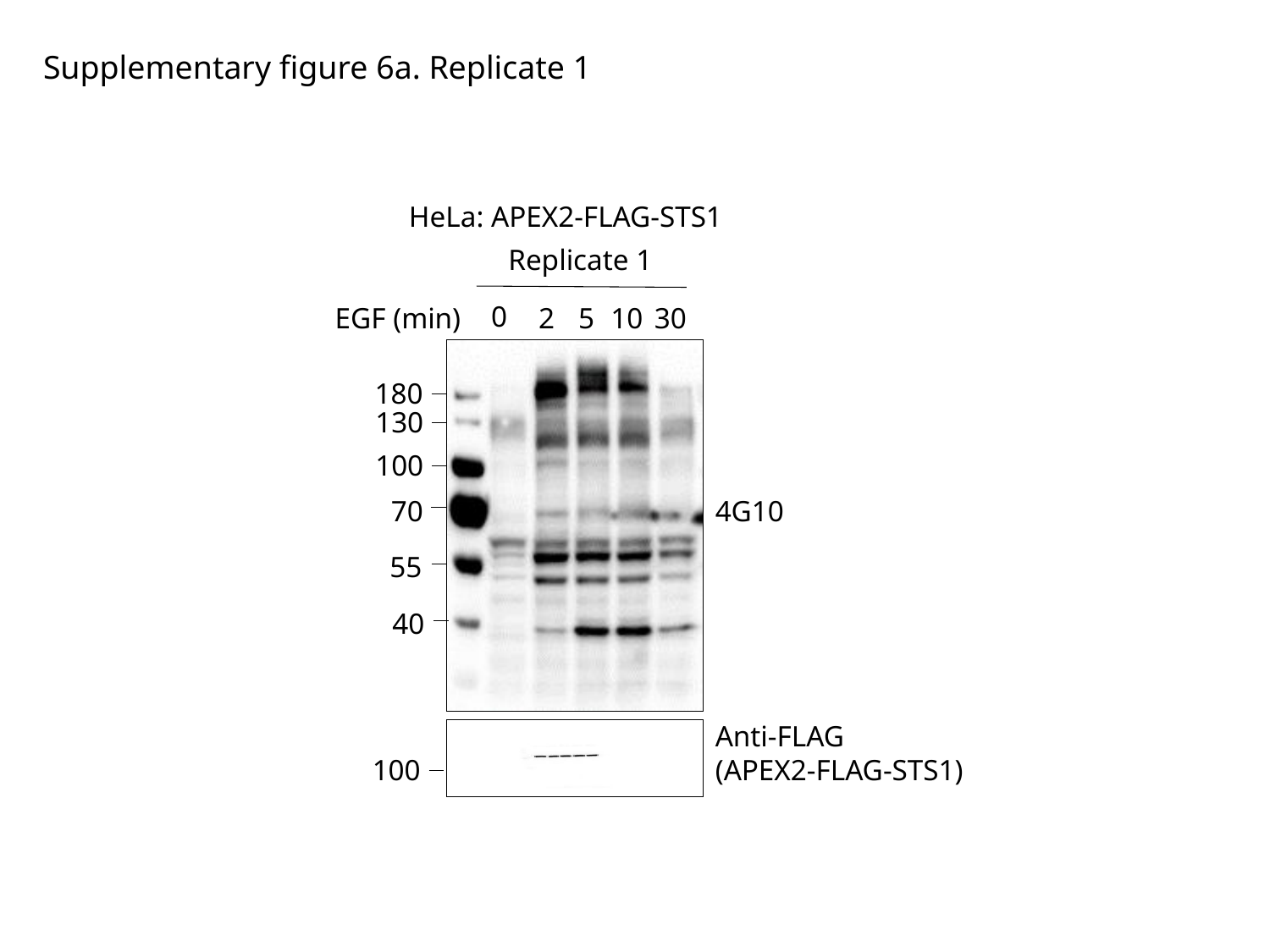

Supplementary figure 6a. Replicate 1
HeLa: APEX2-FLAG-STS1
Replicate 1
0
EGF (min)
2
5
10
30
180
130
100
70
4G10
55
40
Anti-FLAG
(APEX2-FLAG-STS1)
100

## Slide 2
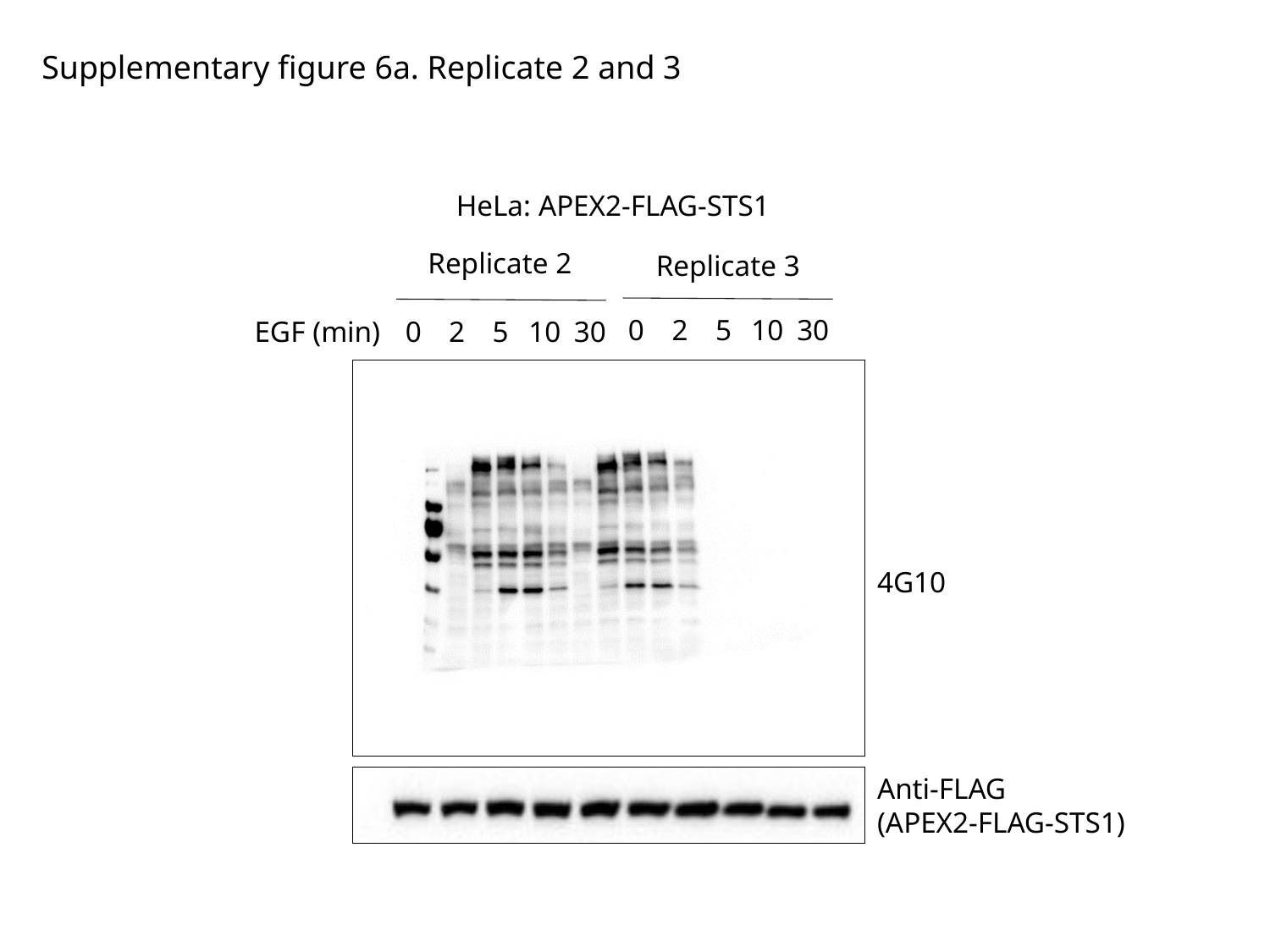

Supplementary figure 6a. Replicate 2 and 3
HeLa: APEX2-FLAG-STS1
Replicate 2
Replicate 3
0
2
5
10
30
EGF (min)
0
2
5
10
30
4G10
Anti-FLAG
(APEX2-FLAG-STS1)

## Slide 3
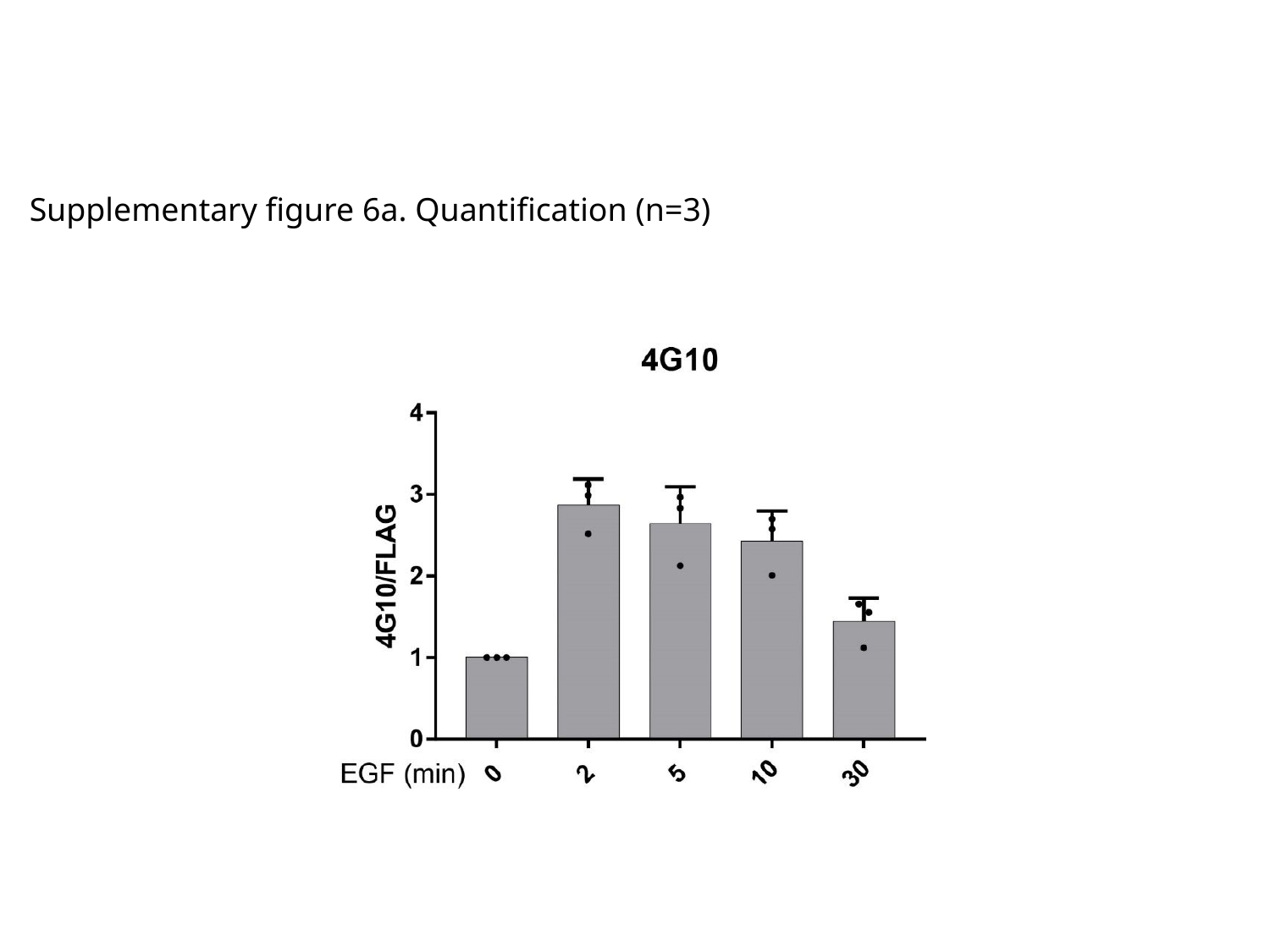

Supplementary figure 6a. Quantification (n=3)
