# Supplementary material for: Spatiotemporal profiling of cytosolic signaling complexes in living cells by selective proximity proteomics
Source: Nat Commun. 2021 Jan 4;12:71. doi: 10.1038/s41467-020-20367-x (PMC7782698; doi:10.1038/s41467-020-20367-x)
Supplement: Supplementary file 16 — Source Data [file 41467_2020_20367_MOESM16_ESM.zip › NCOMMS-20-22505C_sd/WB and IF_Replicates and Quantification/Supplementary Figure 9d/Three replicates.pptx]

## Slide 1
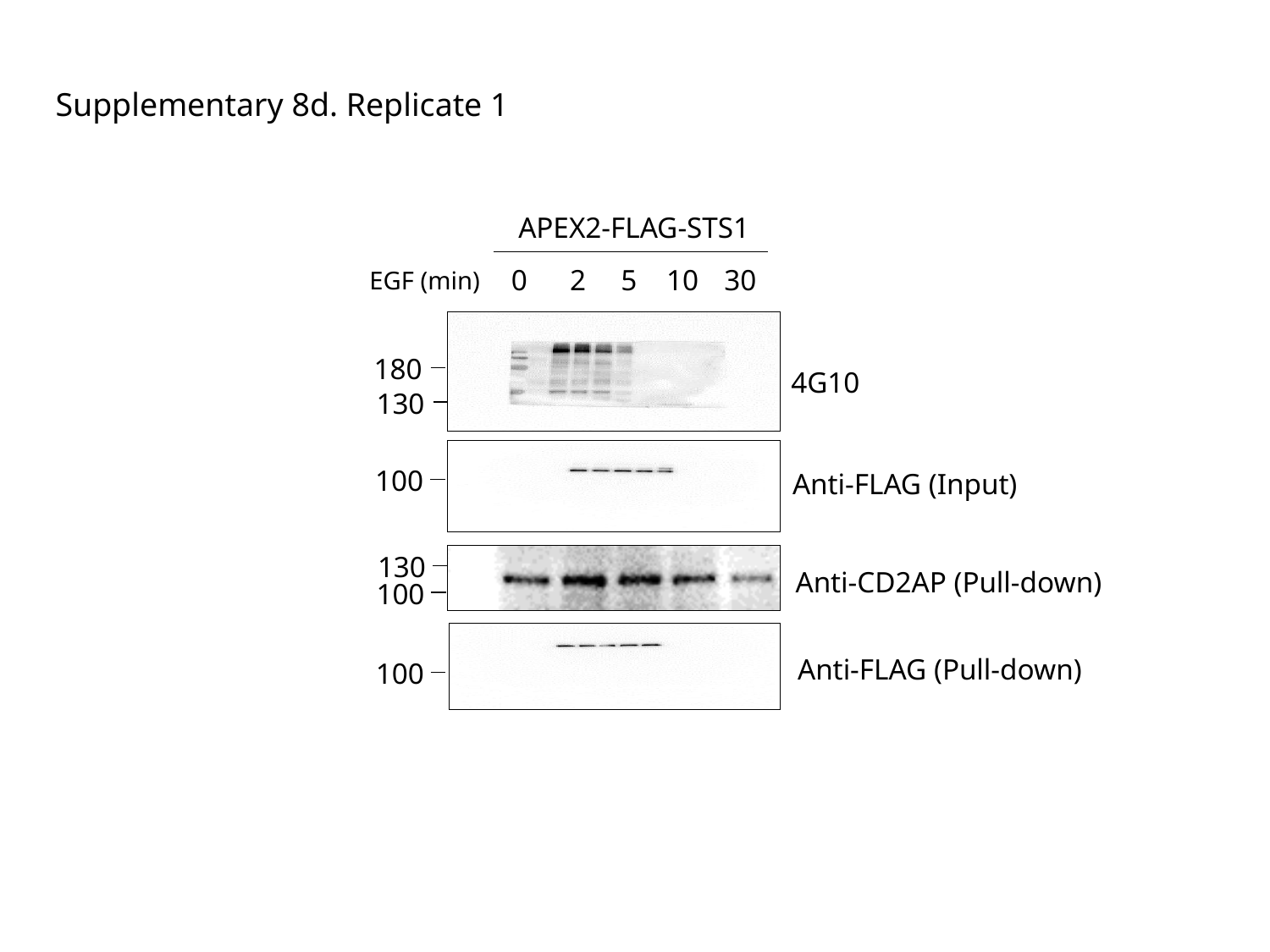

Supplementary 8d. Replicate 1
APEX2-FLAG-STS1
0
2
5
10
30
EGF (min)
180
4G10
130
100
Anti-FLAG (Input)
130
Anti-CD2AP (Pull-down)
100
Anti-FLAG (Pull-down)
100

## Slide 2
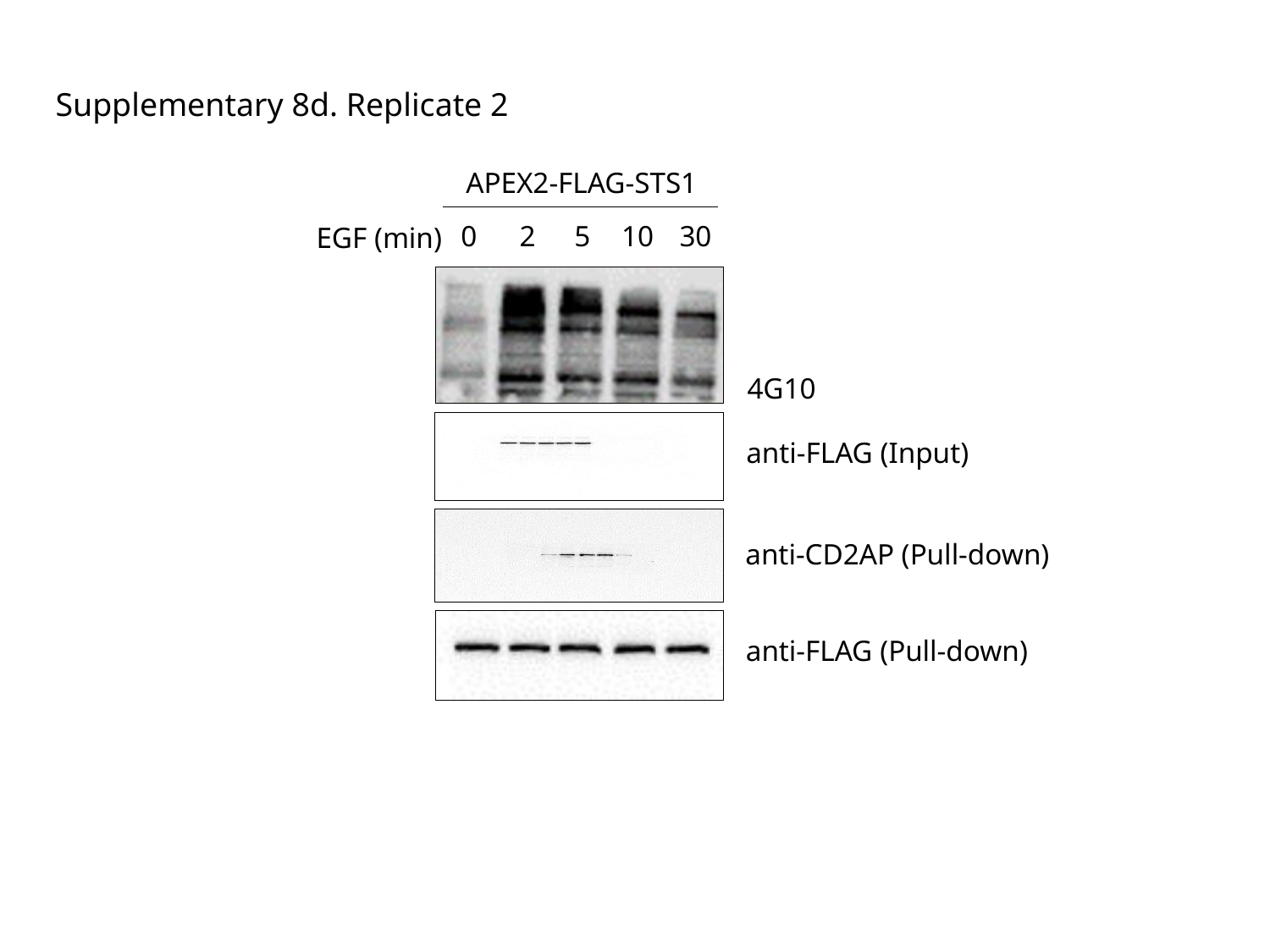

Supplementary 8d. Replicate 2
APEX2-FLAG-STS1
0
2
5
10
30
EGF (min)
4G10
anti-FLAG (Input)
anti-CD2AP (Pull-down)
anti-FLAG (Pull-down)

## Slide 3
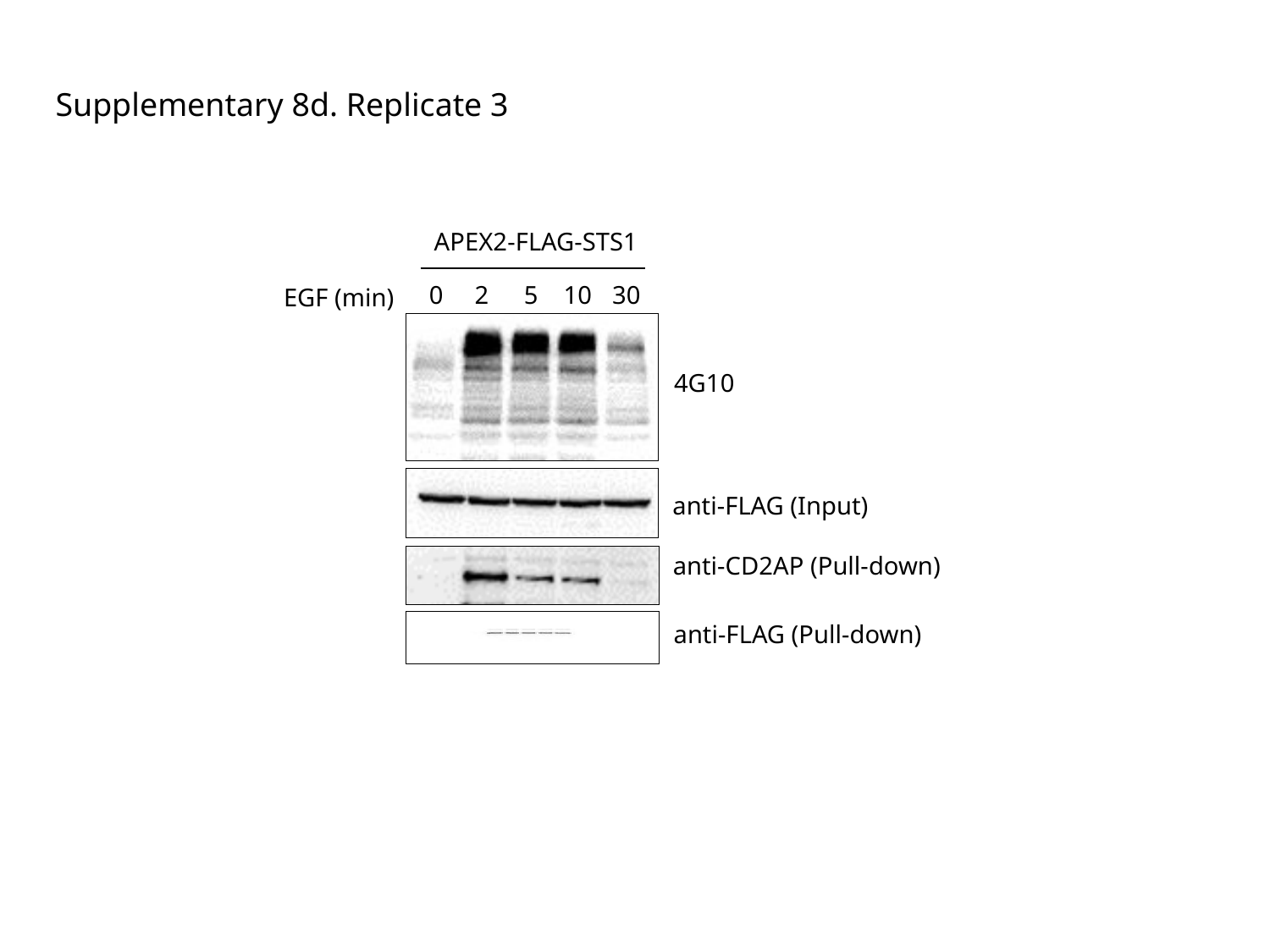

Supplementary 8d. Replicate 3
APEX2-FLAG-STS1
0
2
5
10
30
EGF (min)
4G10
anti-FLAG (Input)
anti-CD2AP (Pull-down)
anti-FLAG (Pull-down)

## Slide 4
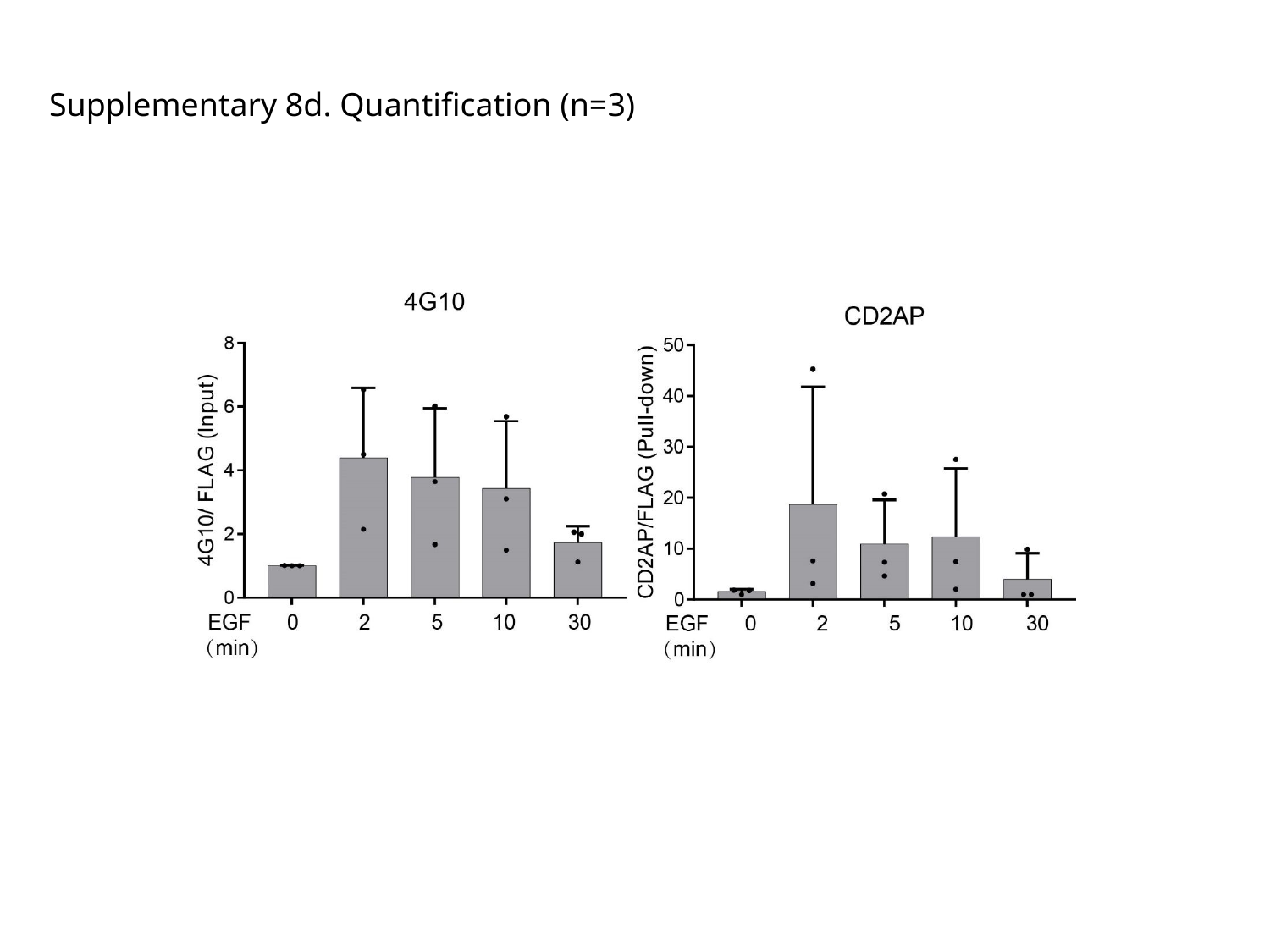

Supplementary 8d. Quantification (n=3)
