# Supplementary material for: Spatiotemporal profiling of cytosolic signaling complexes in living cells by selective proximity proteomics
Source: Nat Commun. 2021 Jan 4;12:71. doi: 10.1038/s41467-020-20367-x (PMC7782698; doi:10.1038/s41467-020-20367-x)
Supplement: Supplementary file 16 — Source Data [file 41467_2020_20367_MOESM16_ESM.zip › NCOMMS-20-22505C_sd/WB and IF_Replicates and Quantification/Supplementary Figure 9e/Three replicates.pptx]

## Slide 1
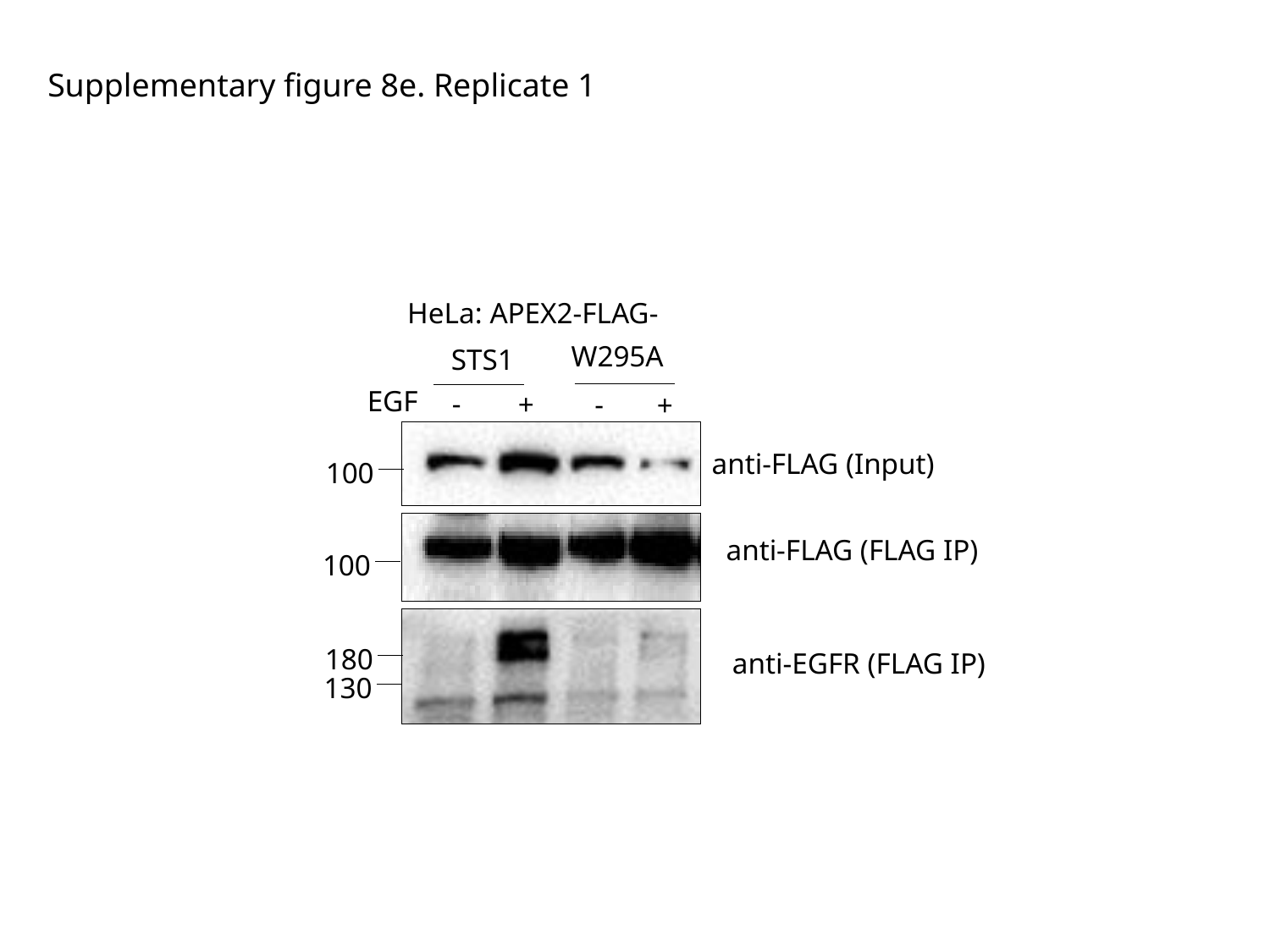

Supplementary figure 8e. Replicate 1
HeLa: APEX2-FLAG-
W295A
STS1
EGF
-
+
-
+
anti-FLAG (Input)
100
anti-FLAG (FLAG IP)
100
180
anti-EGFR (FLAG IP)
130

## Slide 2
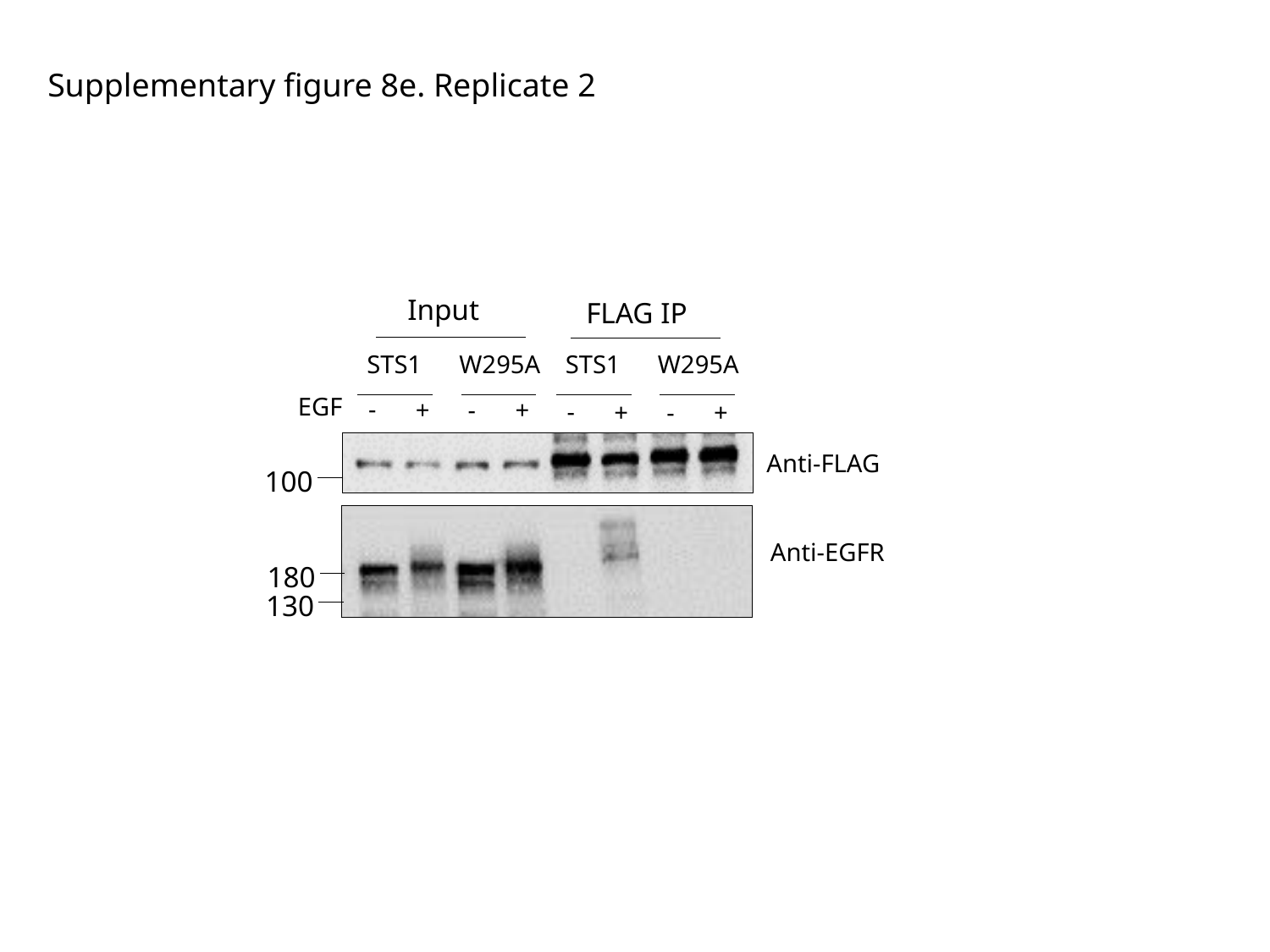

Supplementary figure 8e. Replicate 2
Input
FLAG IP
STS1
W295A
STS1
W295A
EGF
-
+
-
+
-
+
-
+
Anti-FLAG
100
Anti-EGFR
180
130

## Slide 3
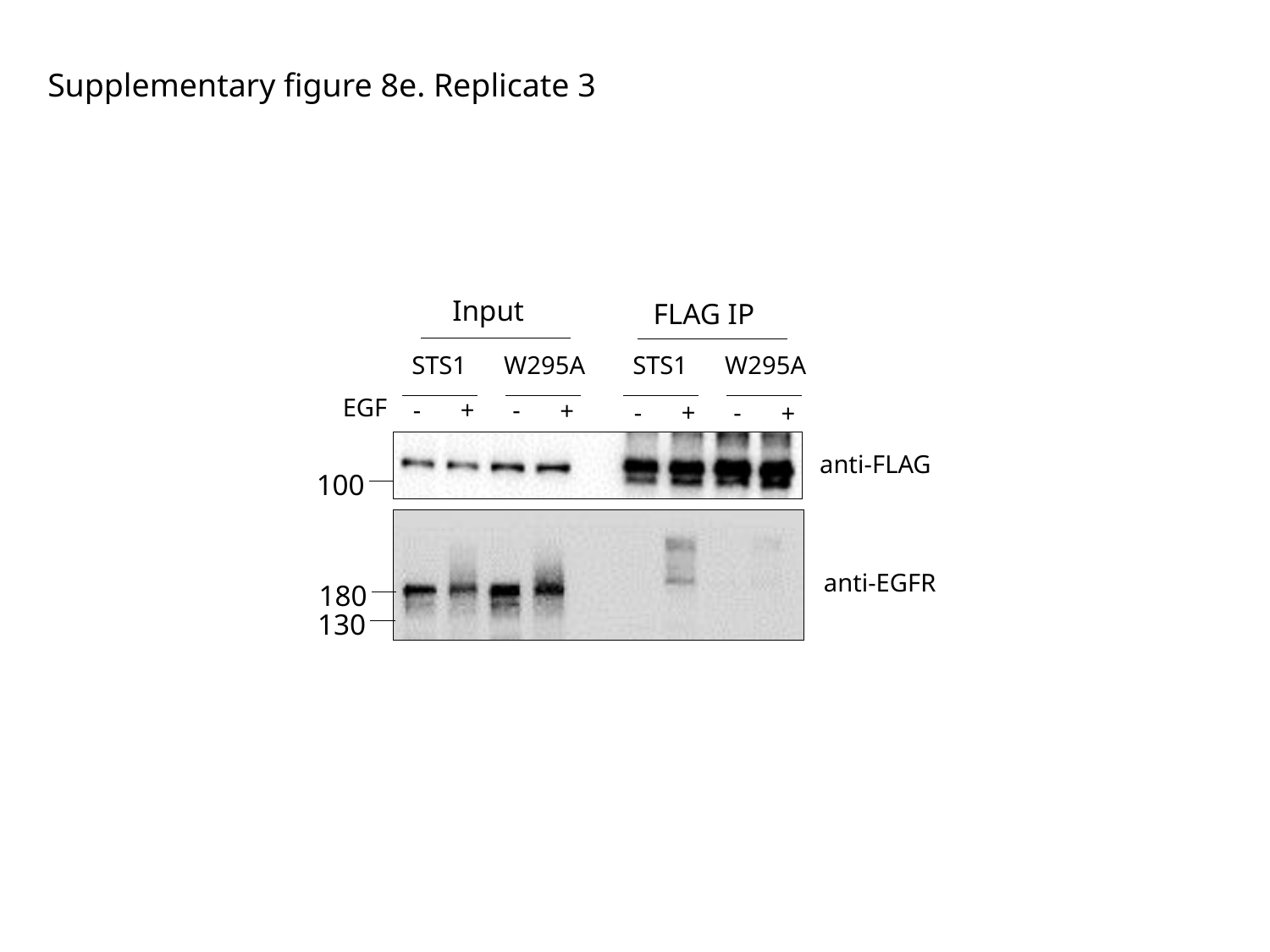

Supplementary figure 8e. Replicate 3
Input
FLAG IP
STS1
W295A
STS1
W295A
EGF
-
+
-
+
-
+
-
+
anti-FLAG
100
anti-EGFR
180
130

## Slide 4
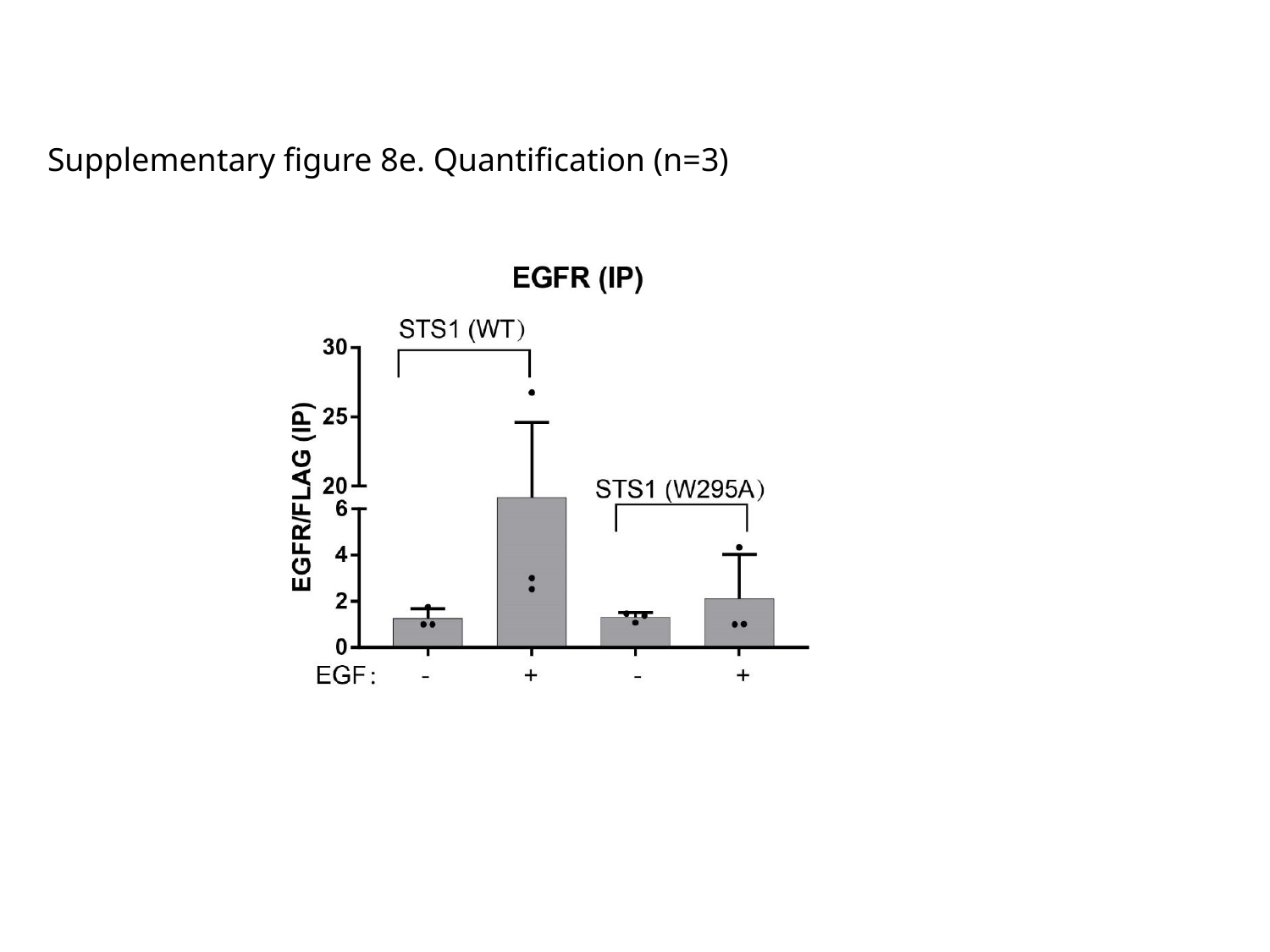

Supplementary figure 8e. Quantification (n=3)
