# Supplementary figures and images for: Spatiotemporal profiling of cytosolic signaling complexes in living cells by selective proximity proteomics (part 2 of 2)
Source: Nat Commun. 2021 Jan 4;12:71. doi: 10.1038/s41467-020-20367-x (PMC7782698; doi:10.1038/s41467-020-20367-x)

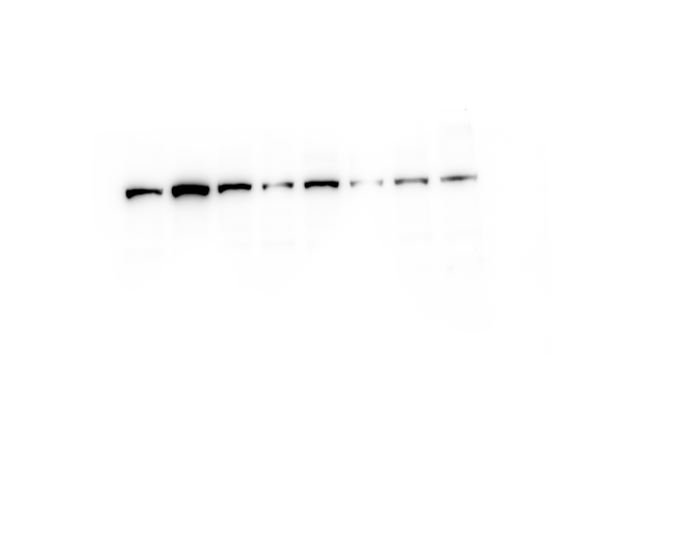

Supplement: Supplementary file 16 — Source Data [file 41467_2020_20367_MOESM16_ESM.zip › NCOMMS-20-22505C_sd/WB and IF_Replicates and Quantification/Supplementary Figure 9e/Replicate 1/anti-FLAG_Input.tif]

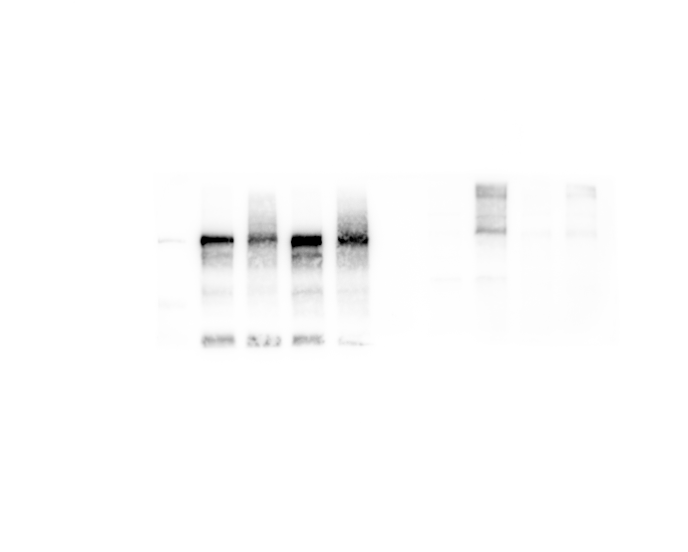

Supplement: Supplementary file 16 — Source Data [file 41467_2020_20367_MOESM16_ESM.zip › NCOMMS-20-22505C_sd/WB and IF_Replicates and Quantification/Supplementary Figure 9e/Replicate 2/anti-EGFR.tif]

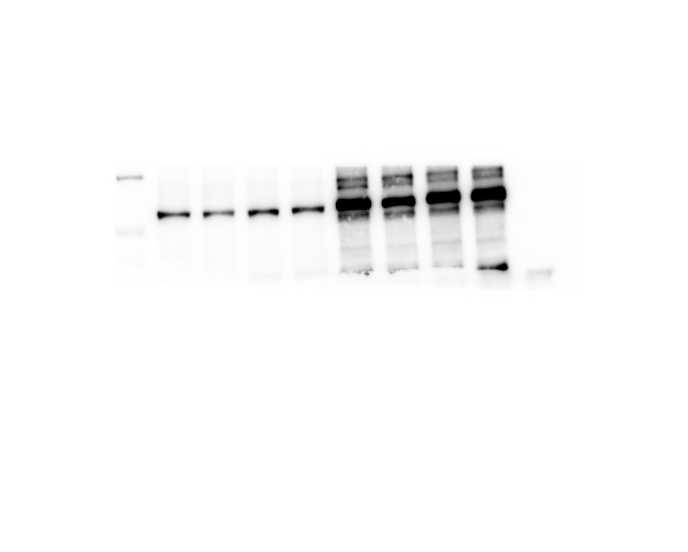

Supplement: Supplementary file 16 — Source Data [file 41467_2020_20367_MOESM16_ESM.zip › NCOMMS-20-22505C_sd/WB and IF_Replicates and Quantification/Supplementary Figure 9e/Replicate 2/anti-FLAG.tif]

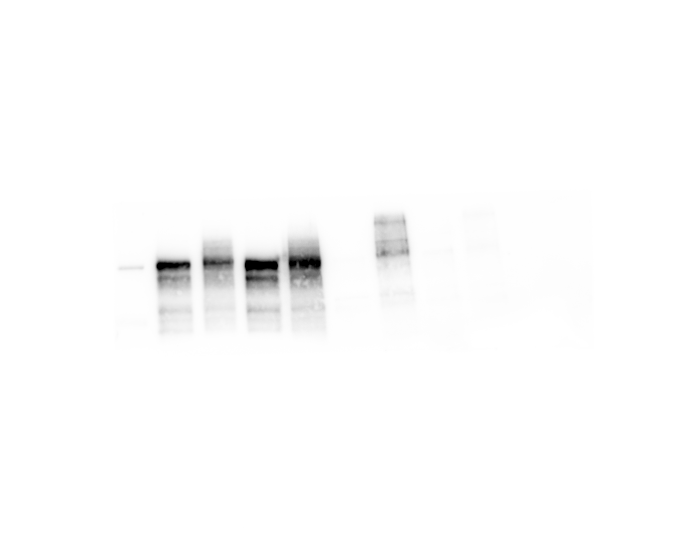

Supplement: Supplementary file 16 — Source Data [file 41467_2020_20367_MOESM16_ESM.zip › NCOMMS-20-22505C_sd/WB and IF_Replicates and Quantification/Supplementary Figure 9e/Replicate 3/anti-EGFR.tif]

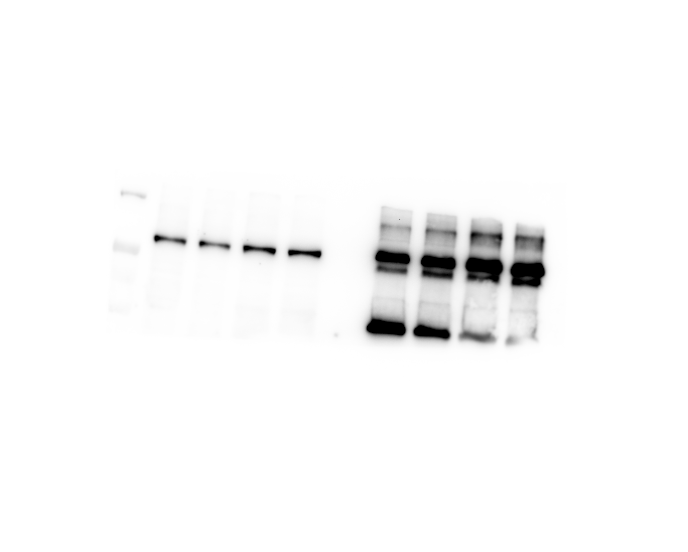

Supplement: Supplementary file 16 — Source Data [file 41467_2020_20367_MOESM16_ESM.zip › NCOMMS-20-22505C_sd/WB and IF_Replicates and Quantification/Supplementary Figure 9e/Replicate 3/anti-FLAG.tif]
